# Substitution of 2-oxoglutarate alters reaction outcomes of the *Pseudomonas savastanoi* ethylene-forming enzyme

**DOI:** 10.1016/j.jbc.2024.107546

**Published:** 2024-07-09

**Authors:** Siddhant Dhingra, Zhihong Zhang, Christopher T. Lohans, Lennart Brewitz, Christopher J. Schofield

**Affiliations:** Chemistry Research Laboratory, Department of Chemistry and the Ineos Oxford Institute for Antimicrobial Research, University of Oxford, Oxford, United Kingdom

**Keywords:** ethylene-forming enzyme, ethylene synthesis, 2-oxoglutarate / 2OG / α-ketoglutarate dependent dioxygenase, bifurcating reaction pathway, alternative substrates, 1-aminocyclopropane-1-carboxylic acid oxidase / ACCO, signaling mechanisms, biocatalysis, sustainability

## Abstract

In seeding plants, biosynthesis of the phytohormone ethylene, which regulates processes including fruit ripening and senescence, is catalyzed by 1-aminocyclopropane-1-carboxylic acid (ACC) oxidase. The plant pathogen *Pseudomonas savastanoi* (previously classified as: *Pseudomonas syringae*) employs a different type of ethylene-forming enzyme (psEFE), though from the same structural superfamily as ACC oxidase, to catalyze ethylene formation from 2-oxoglutarate (2OG) in an arginine dependent manner. psEFE also catalyzes the more typical oxidation of arginine to give L-Δ^1^-pyrroline-5-carboxylate (P5C), a reaction coupled to oxidative decarboxylation of 2OG giving succinate and CO_2_. We report on the effects of C3 and/or C4 substituted 2OG derivatives on the reaction modes of psEFE. ^1^H NMR assays, including using the pure shift method, reveal that, within our limits of detection, none of the tested 2OG derivatives is converted to an alkene; some are converted to the corresponding β-hydroxypropionate or succinate derivatives, with only the latter being coupled to arginine oxidation. The NMR results reveal that the nature of 2OG derivatization can affect the outcome of the bifurcating reaction, with some 2OG derivatives exclusively favoring the arginine oxidation pathway. Given that some of the tested 2OG derivatives are natural products, the results are of potential biological relevance. There are also opportunities for therapeutic or biocatalytic regulation of the outcomes of reactions catalyzed by 2OG-dependent oxygenases by the use of 2OG derivatives.

2-Oxoglutarate (2OG, **1**) and non-heme Fe(II) dependent oxygenases are widely distributed in aerobic biology; catalysis by them typically involves coupling the oxidative decarboxylation of 2OG to give succinate and CO_2_ with the two electron oxidation of their prime substrates (1). There is a wealth of biochemical data showing the efficiency of 2OG as a cosubstrate for the 2OG oxygenases (1). It is, however, feasible that 2OG oxygenases employ other (co)substrates instead of, or as well as, 2OG in cells; indeed, 2-oxoacids other than 2OG are natural products and many are abundant in cells (2, 3, 4, 5). This proposal is supported by the flexibility of 2OG oxygenases with respect to the type of their prime substrate oxidations, including hydroxylation-, dealkylation-, halogenation-, desaturation-, ring formation-, and ring expansion-reactions (1). The flexibility of 2OG oxygenase-type active sites is further highlighted by the existence of enzymes belonging to the same structural cupin fold subfamily as the 2OG oxygenases, but which catalyze oxidations without a need for 2OG as a cosubstrate. Two prominent examples are isopenicillin N synthase (IPNS) (6, 7) and 1-aminocyclopropane-1-carboxylic acid (ACC) oxidase (ACCO), the latter of which catalyzes the biosynthesis of the plant hormone ethylene (Fig. 1*A*) (8, 9), which regulates processes including fruit ripening and senescence (9, 10, 11).

**Figure 1:**
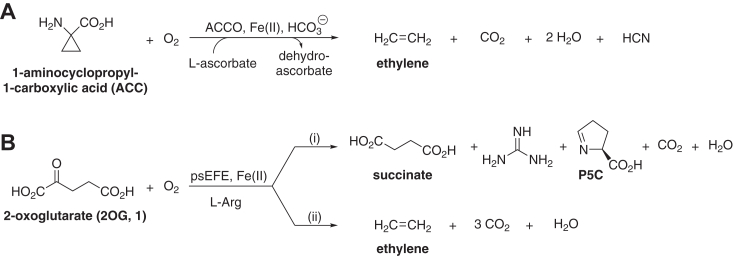
**The bifurcating reaction of 2OG during psEFE catalysis.** (*A*) plant ACCOs catalyze ethylene formation from 1-aminocyclopropane-1-carboxylic acid (ACC) (8, 9). The role(s) of L-ascorbate and bicarbonate in ACCO catalysis in cells is unclear. (*B*) psEFE catalyzes: (i) the oxidative decarboxylation of 2OG (**1**) coupled to C5 oxidation of L-arginine to give 5-hydroxyarginine which fragments to guanidine and L-Δ^1^-pyrroline-5-carboxylate (P5C), and (ii) fragmentation of 2OG (**1**) to give ethylene and CO_2_, a reaction outcome in which Arg is not modified (11, 37, 39, 40, 41).

The human lysine- and tryptophan-catabolite 2-oxoadipate (2OA, **2**) (12, 13), the carbon scaffold of which is elongated by one methylene compared to 2OG, can sustain catalysis *in vitro* by some human and bacterial 2OG oxygenases (14, 15, 16, 17, 18, 19, 20, 21). We and others have shown that the cosubstrate scope of some, but not all, human 2OG oxygenases extends to C3 and/or C4 substituted 2OG derivatives, some of which are natural products (22, 23, 24, 25, 26). Recently reported mass spectrometry (MS)-based assays with isolated recombinant human aspartate/asparagine-β-hydroxylase (AspH) and factor inhibiting hypoxia-inducible factor HIF-α (FIH), which both catalyze the 2OG-dependent post-translational hydroxylation of aspartate and asparagine residues in proteins (27, 28, 29, 30, 31, 32, 33, 34), have revealed that AspH and FIH prefer structurally distinct sets of C3 and/or C4 substituted 2OG derivatives as cosubstrates (23). Kinetic studies implied that some of the tested 2OG derivatives react as efficiently as 2OG with AspH, FIH, and human Jumonji-C (JmjC) domain-containing protein 5 (JMJD5) (22, 23, 24). By contrast, the human JmjC histone lysine-specific *N*^ε^-demethylases 4A and 4E (KDM4A and KDM4E) have not been observed to react efficiently with any of the tested 2OG derivatives other than 2OG (24, 25). The combined evidence indicates the potential of specific 2OG oxygenases to selectively react with and, in some cases, be inhibited by, 2OG derivatives (23).

We have proposed that 2OG derivatives may react differently with the same enzyme to give different products/product ratios to those observed with 2OG (23). To investigate this possibility, we envisioned that the 2OG-dependent ethylene/succinate-forming enzyme from *Pseudomonas savastanoi* (previously classified as: *Pseudomonas syringae*) pv. phaseolicola (psEFE; EC:1.13.12.19) (35, 36) would be a particularly useful model, because with 2OG it catalyzes a bifurcating reaction (37), *i.e.*, (i) the oxidative decarboxylation of 2OG coupled to the oxidation of L-arginine (Arg) (38) to give succinate, CO_2_, and 5-hydroxy arginine that fragments giving guanidine and L-Δ^1^-pyrroline-5-carboxylate (P5C), and (ii) a Grob-type fragmentation of 2OG to give ethylene and three molecules of CO_2_ (Fig. 1*B*) (11, 37, 39, 40, 41). psEFE thus has potential for the biocatalytic production of ethylene and other industrially important alkenes (35, 36, 42, 43).

Limited studies with psEFE and 2OG derivatives have been reported (39, 44, 45). psEFE can catalyze the hydroxylation of Arg using 2OA (**2**) as a cosubstrate, but it apparently does not catalyze the conversion of **2** into propene (39). The reactivity of psEFE with five C4 substituted 2OG derivatives was recently reported using infrared (IR) and LCMS assays, the results of which indicated that wild-type (wt) psEFE does not catalyze the fragmentation of C4-alkyl- and/or C4-hydroxyl-substituted 2OG derivatives to give alkenes, though it did catalyze fragmentation of 4-fluoro-2OG into fluoroethylene (44). All of the five tested C4 substituted 2OG derivatives sustained the psEFE-catalyzed hydroxylation of Arg to give the corresponding succinate derivatives; additionally, psEFE catalyzed the decarboxylative β-oxidation of all of the five tested C4 substituted 2OG derivatives to give the corresponding β-hydroxypropionate products and CO_2_ (44).

Here we report ^1^H NMR studies investigating the reactivity of psEFE with a set of 18 C3 and/or C4 substituted 2OG derivatives; the reaction of C3 substituted 2OG derivatives with psEFE has not been previously tested. Oxidative decarboxylation of 2OG derivatives to give diacid (succinate) derivatives coupled to Arg oxidation was observed in some cases. Although no evidence for alkene formation was accrued, some of the 2OG derivatives reacted to give alcohol (β-hydroxypropionate) derivatives. Notably, the results provide clear evidence that the ratio of bifurcating reaction outcomes can be affected by the nature of the 2OG derivatization, supporting the proposal that the outcome of 2OG oxygenase catalysis can be altered in a 2-oxoacid (co)substrate dependent manner.

## Results

### NMR assays for monitoring psEFE catalysis

^1^H NMR assays were employed to investigate the reactivity of C3 and/or C4 substituted 2OG derivatives with psEFE, because they can simultaneously: (i) monitor substrate depletion and product formation, (ii) enable quantification of substrate turnover by comparison with an internal standard, and (iii) enable the structural characterization of reaction products. Prior to investigating the reactivity of C3 and/or C4 substituted 2OG derivatives with psEFE, we optimized the reported ^1^H NMR assay conditions using 2OG (**1**) as a cosubstrate (40). The optimized standard conditions were: *P. savastanoi* (previously: *P. syringae*) pv. phaseolicola ethylene/succinate-forming enzyme (psEFE; UNIPROT ID: P32021; EC:1.13.12.19) (40) (10 or 30 μM), L-arginine (Arg; 500 μM), L-ascorbate (LAA; 500 μM), 2OG/2OG derivative (400 μM), and Fe(II) (50 μM) in buffer (50 mM sodium phosphate, 10%_v/v_ D_2_O, pH 7.4) at 25 °C (Fig. S1). Exchanging the previously reported Tris-*d*_*11*_ buffer (40) for phosphate buffer improved detection of P5C by ^1^H NMR (singlet, δ ∼ 7.70 ppm). 3-(Trimethylsilyl)-2,2,3,3-tetradeuteropropionic acid (46) (TMSP-*d*_*4*_; 800 μM) was used as an inert internal standard to enable quantification of turnover.

No reaction products other than succinate (singlet, δ ∼ 2.32 ppm), ethylene (singlet, δ ∼ 5.35 ppm), and P5C (5-H: singlet, δ ∼7.70 ppm) were observed by ^1^H NMR (600 MHz) analysis of the crude reaction mixture under the optimized conditions when using 2OG as a cosubstrate (Fig. 1); note that all peaks are assigned in the psEFE-catalyzed conversion of 2OG (Fig. S2). This result was confirmed by pure shift ^1^H NMR (47) (700 MHz) analysis of the reaction mixture (Fig. S3). The pure shift method increases the sensitivity for detection of reaction products by broadband homonuclear decoupling that collapses proton signals with multiplet patterns into singlets at near the respective chemical shift (48, 49, 50). Arg depletion could not be quantified by ^1^H NMR analysis, because the Arg signals overlapped with those of the P5C product. Signals corresponding to dehydroascorbate, arising from oxidation of LAA, were observed at low levels (Fig. S4).

**Figure 2:**
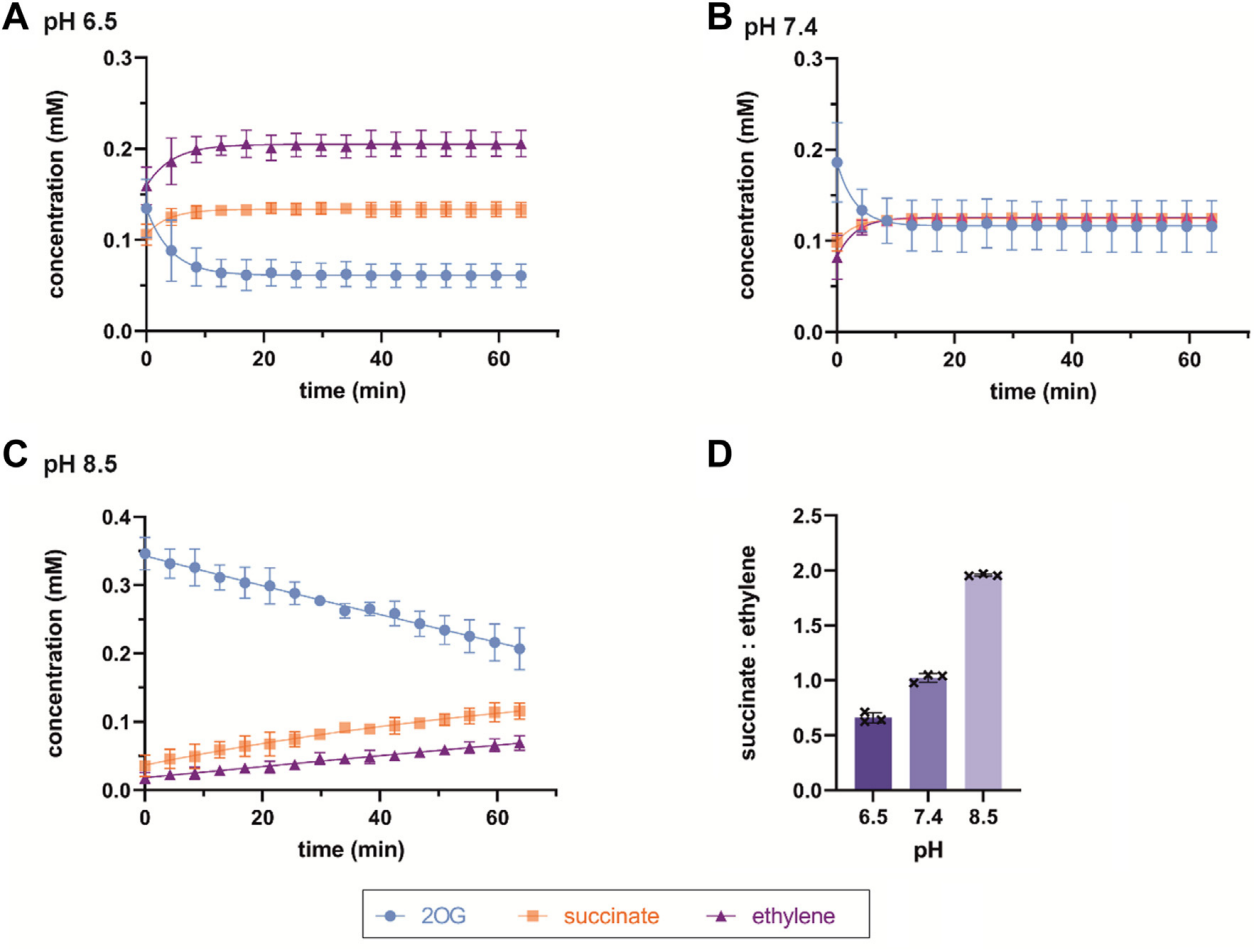
**Reaction conditions affect the product ratio of psEFE catalysis when using 2OG(1)as a (co)substrate.** The ethylene:succinate ratios estimated by ^1^H NMR (600 MHz) analysis of the crude reaction mixtures vary with pH: (*A*) 1.5 ± 0.1:1.0 at pH 6.5, (*B*) 1.0 ± 0.1:1.0 at pH 7.4, and (*C*) 0.5 ± 0.1:1.0 at pH 8.5. (*D*) succinate:ethylene ratios. Ethylene formation was indirectly estimated by subtracting the normalized integral of succinate (obtained *via* comparison with TMSP-*d*_*4*_) from that of 2OG (**1**). The time scales were calibrated to the end of the acquisition of the first ^1^H NMR experiment following psEFE addition to the reaction mixture (t = 0 min), by which time low levels of conversion were manifest. Conditions: 400 μM 2OG (**1**), 500 μM L-arginine, 500 μM L-ascorbate, 50 μM Fe(II), 800 μM TMSP-*d*_*4*_, and 2 μM psEFE in buffer (50 mM sodium phosphate, 10%_v/v_ D_2_O) at 25 °C; note that full conversion was observed after ∼12 h or when increasing psEFE concentrations. Reaction profiles are a mean of three independent runs (n = 3; mean ± SD).

The results of integrating the relevant peaks of ^1^H NMR assays of up to >1 h duration after psEFE addition imply that the formation of succinate and P5C are coupled, *i.e.*, they are produced in a ∼1:1 ratio, at least in early time points (Fig. 2). The P5C signal was, however, observed to decrease at later time points, likely due to relatively slow non-enzymatic reaction(s). The ^1^H NMR-based data for ethylene production obtained by integration of the ethylene peak should be regarded as approximate, because reactions were performed in open 3 mm NMR tubes to enable sufficient O_2_ supply. Ethylene formation was thus indirectly estimated by ^1^H NMR analysis of the crude reaction mixtures by subtracting the normalized integral for succinate (obtained *via* quantitative comparison with that for TMSP-*d*_*4*_; T_1_ : 2 s) from that for 2OG. Analysis using this method showed that succinate production correlates stoichiometrically with that of Arg hydroxylation to give P5C (Fig. S5). This method was found to be more reliable than direct integration of the ethylene signal; note that quantitative comparison of the 2OG integrals with those of TMSP-*d*_*4*_ confirms the 1:2 stoichiometry of 2OG and TMSP-*d*_*4*_ in the initial reaction mixture. Using this method of analysis, the ratio of ethylene:succinate formation was estimated to be 1.0:1.0 ± 0.1 (n = 3; mean ± standard deviation, SD), which differs from reported ethylene:succinate ratios obtained by scintillation or gas chromatography (GC) methods, *i.e.*, ∼2:1 (37, 39, 41). The observed difference in the ethylene:succinate ratios may be the result of the different assay employed and/or the different reaction conditions, as recently proposed (11).

The possibility that the ratio of ethylene:succinate formation varies with the reaction conditions was tested by monitoring psEFE catalysis at different pH values, using 2OG as a cosubstrate. The results reveal that the estimated ethylene:succinate ratios vary with the pH, *i.e.*, the ethylene:succinate ratio apparently decreases from ∼1.5:1.0 at pH 6.5 to ∼1:1 at pH 7.4, then to ∼0.5:1.0 at pH 8.5 (Fig. 2). Note that the product ratios appeared to be constant over the time course of analyses at a given pH. Thus, it appears that more acidic conditions (pH 6.5) may favor ethylene over succinate formation, although other factors than the pH may affect the ethylene:succinate ratios considering that they are reported to be constant ∼2:1 between pH 6.0 and 8.5 when using different conditions and assay methods (39). Increased levels of turnover were also observed under more acidic conditions (pH 6.5). Although the underlying reason(s) for the observed changes in the ethylene:succinate ratios at different pHs are presently unclear, these observations may be of interest from a biocatalytic perspective for optimizing conversion of 2OG to ethylene rather than succinate.

### psEFE catalyzes the conversion of 2-oxoadipate to γ-hydroxybutyrate

**Figure 3:**
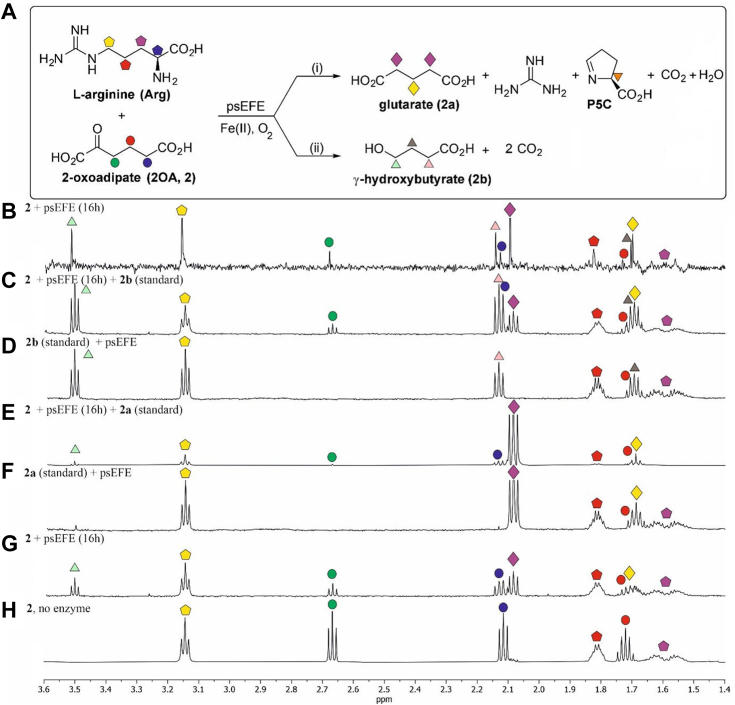
**psEFE catalyzes the bifurcating reaction of 2-oxoadipate to give glutarate and γ-hydroxybutyrate.** (*A*) psEFE catalyzes the bifurcating decarboxylative oxidation of 2-oxoadipate (2OA, **2**) to give either (i) glutarate (**2a**) or (ii) γ-hydroxybutyrate (**2b**); propene was not observed. (*B*) analysis (1.4–3.6 ppm) of the crude reaction mixture by pure shift ^1^H NMR (700 MHz) (47, 48, 49, 50) 16 h post incubation with psEFE indicates that psEFE does not catalyze propene formation from **2**. Note that for the pure shift ^1^H NMR analysis, the observed peak may not necessarily be at the center of the peak obtained when using standard NMR analysis (47, 48, 49, 50); (*C*–*H*) ^1^H NMR (600 MHz) analysis (1.4–3.7 ppm) of (*C*) a reaction mixture of psEFE and **2** spiked with an authentic sample of **2b** (standard) 16 h post addition of psEFE, (*D*) **2b** (standard) in the absence of **2** under standard conditions; (*E*) a reaction mixture of psEFE and **2** spiked with authentic **2a** (standard) 16 h post addition of psEFE, (*F*) **2a** (standard) in the absence of **2** under standard conditions; (*G*) a reaction mixture of psEFE and **2** 16 h post addition of psEFE; (*H*) **2** under standard conditions in the absence of psEFE. Conditions: 400 μM 2OA (**2**), 500 μM L-arginine, 500 μM L-ascorbate, 50 μM Fe(II), 800 μM TMSP-*d*_*4*_, 10 μM psEFE, and, if appropriate, 2 mM of an appropriate authentic standard in buffer (50 mM phosphate, pH 7.4, 10%_v/v_ D_2_O). Representative ^1^H NMR spectra from three independent repeats are shown (n = 3).

**Figure 4:**
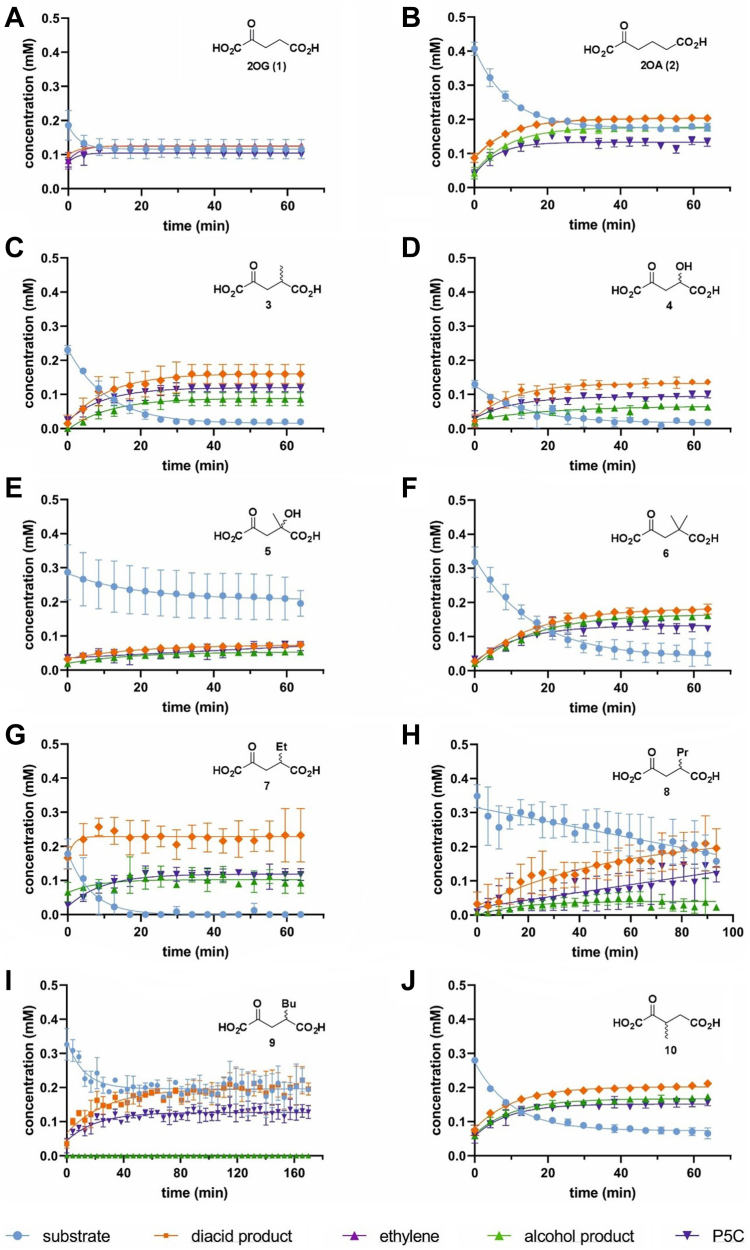
^**1**^**H NMR assays enable measurement of product ratios of psEFE-catalyzed reactions with 2OG derivatives.** Reaction profiles of the psEFE-catalyzed oxidation of (*A*) 2OG (**1**), (*B*) 2OA (**2**), (*C*) 4-methyl-2OG (**3**), (*D*) 4-hydroxy-2OG (**4**), (*E*) 4-hydroxy-4-methyl-2OG (**5**), (*F*) 4,4-dimethyl-2OG (**6**), (*G*) 4-ethyl-2OG (**7**), (*H*) 4-propyl-2OG (**8**), (*I*) 4-butyl-2OG (**9**), and (*J*) 3-methyl-2OG (**10**). Conditions: 400 μM 2-oxoacid, 500 μM L-arginine, 500 μM L-ascorbate, 50 μM Fe(II), 800 μM TMSP-*d*_*4*_, 2 μM psEFE (for 2OG, **1**) or 10 to 30 μM psEFE (for **2** and 2OG derivatives) in buffer (50 mM sodium phosphate, pH 7.4, 10%_v/v_ D_2_O) at 25 °C. 2OG derivatives were employed as racemic mixtures. Time scales were calibrated to the end of the first ^1^H NMR analysis following psEFE addition to the reaction mixture (t = 0 min), by which time low levels of conversion were manifest. Results are the mean of independent triplicates (n = 3; mean ± SD); graphs were plotted using GraphPad Prism 9.3.0 using non-linear regression analysis and a single-phase decay model. Representative ^1^H NMR spectra are shown in Figs. S9–S42.

We then reinvestigated the reaction of psEFE with 2-oxoadipate (2OA, **2**), which binds in a similar manner as 2OG (**1**) (PDB ID: 5V2Z (51)) and which is a reported psEFE cosubstrate (39). Comparison of reaction profiles over >1 h reveals ∼60% consumption of **2** under the standard conditions (Figs. 3 and 4*B*; S6–S8). Neither increasing the psEFE concentration (from 10 to 30 μM), nor increasing the reaction time to ∼20 h improved overall levels of 2OA consumption, suggesting possible product inhibition, though assays in the presence of added glutarate (**2a**), γ-hydroxybutyrate (**2b**) or guanidine (400 μM) suggested that these do not affect psEFE catalysis. As reported, our results reveal production of glutarate from **2** (39), the formation of which, as for 2OG (38), is tightly coupled to that of P5C (Fig. 4*B*). No evidence for propene formation was observed, including by pure shift ^1^H NMR analysis (47, 48, 49, 50) (Fig. 3*B*), even after prolonged reaction (>20 h), in accord with the literature (39). Note that for the pure shift ^1^H NMR analysis, the observed peak may not necessarily be at the center of the peak obtained when using standard NMR analysis (47, 48, 49, 50). Time-dependent formation of **2b** was observed (Figs. 3 and 4*B*), in support of a report appearing during the course of our investigations (44). Formation of β-hydroxypropionate, the corresponding putative product arising from decarboxylative β-oxidation of 2OG, was not observed by ^1^H NMR with 2OG as a cosubstrate (Figs. S2 and S3), in accord with previous results (39, 44).

The results with 2OA (**2**) show that the γ-hydroxybutyrate (**2b**):glutarate (**2a**, produced from **2**) and ethylene (**1b**):succinate (**1a**, produced from 2OG) ratios differ. The ratio of γ-hydroxybutyrate:glutarate formation from **2** (*i.e.*, 1.0:2.1 ± 0.1; n = 3 ± SD) differs from that estimated for ethylene:succinate formation from 2OG (*i.e.*, 1:0:1.0 ± 0.1). Note that this relationship is not affected by loss of ethylene from solutions and that the γ-hydroxybutyrate (**2b**):glutarate (**2a**) ratio with **2** measured by ^1^H NMR differs from that reported using an LCMS-based assay (*i.e.*, ∼1:1) (44), possibly reflecting different assay conditions, as previously observed when using 2OG as a (co)substrate (Fig. 2). The observations with **2** encouraged us to carry out studies on the reactivity of psEFE with synthetic 2OG derivatives, with the objective of exploring the possibility of alterations in product ratios.

### The 2OG substitution pattern affects the reaction outcomes of psEFE catalysis

**Figure 5:**
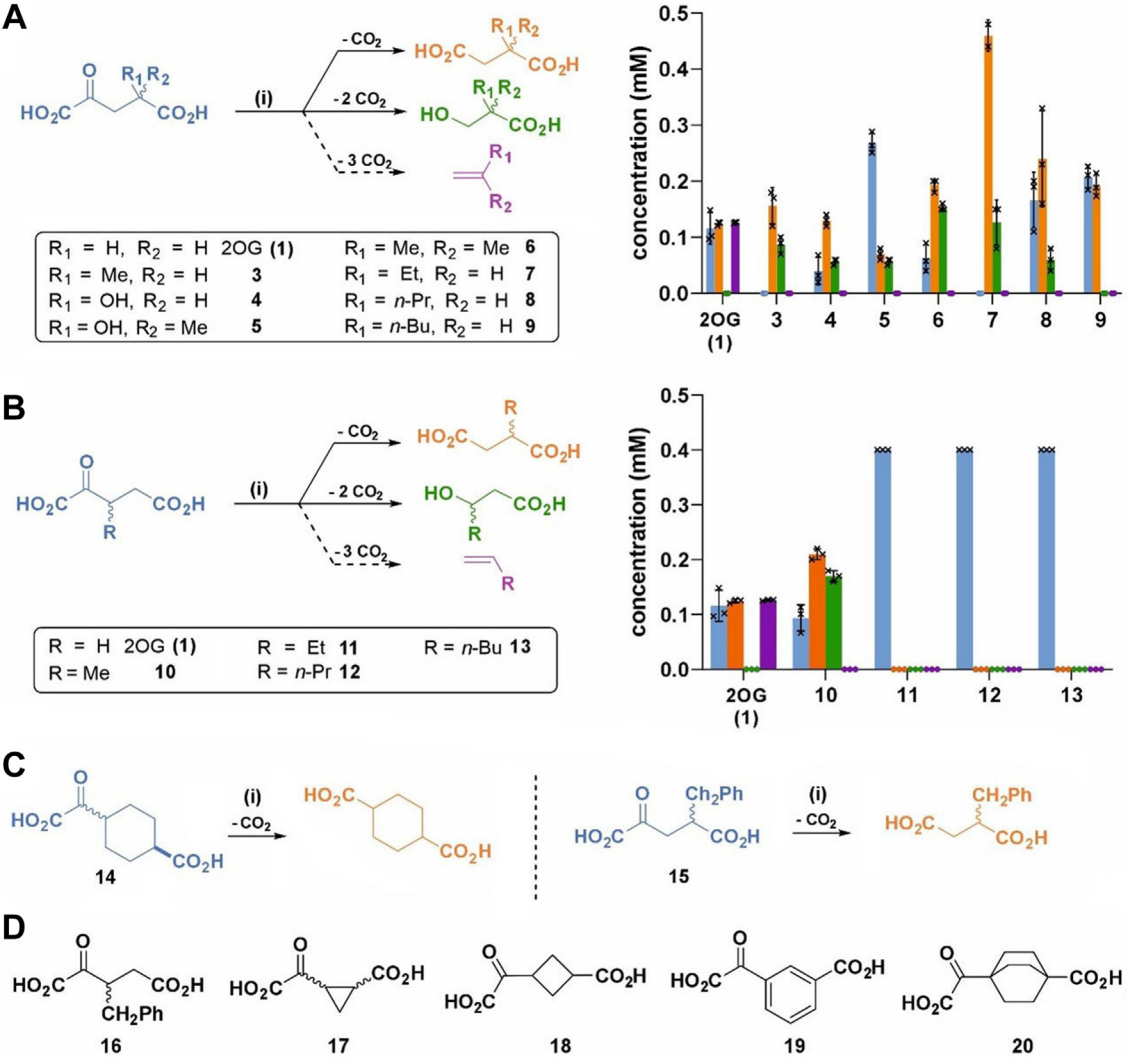
**The substitution pattern of C3 and/or C4 substituted 2OG derivatives affects reaction outcomes of psEFE catalysis.** (*A*) reaction outcomes of psEFE (10 μM; for 2OG (**1**): 2 μM; for **9**: 30 μM) catalysis with C4 substituted 2OG derivatives (400 μM). (*B*) reaction outcomes of psEFE (10 μM; for 2OG (**1**): 2 μM) catalysis with C3 substituted 2OG derivatives (400 μM). (*C*) 2OG derivatives which sustain psEFE-catalyzed Arg hydroxylation as cosubstrates, but which do not give alcohol products. In each case, the identity of the succinate derivative product was confirmed by spiking the reaction mixture with an authentic synthetic sample. However, levels of conversion could not be accurately determined using ^1^H NMR analyses (600 MHz) due to overlapping signals of starting material and product. Spiking with the potential corresponding alcohol products revealed that they are not formed, within limits of detection (Figs. S43–S48). Evidence for the corresponding alcohol product was also not observed by pure shift ^1^H NMR (Fig. S48). (*D*) 2OG derivatives which do not react with psEFE (30 μM) under the tested conditions (Figs. S49–S56). 2OG derivatives were typically prepared from cyanosulfur ylids as racemic mixtures as reported (22); **14** was used as a mixture of diastereomers, dr (*trans*:*cis*) ∼ 5:1. Note it is possible that stereoselective conversion(s) may occur in some cases. Results are the mean of independent triplicates (n = 3; mean ± SD).

**Figure 6:**
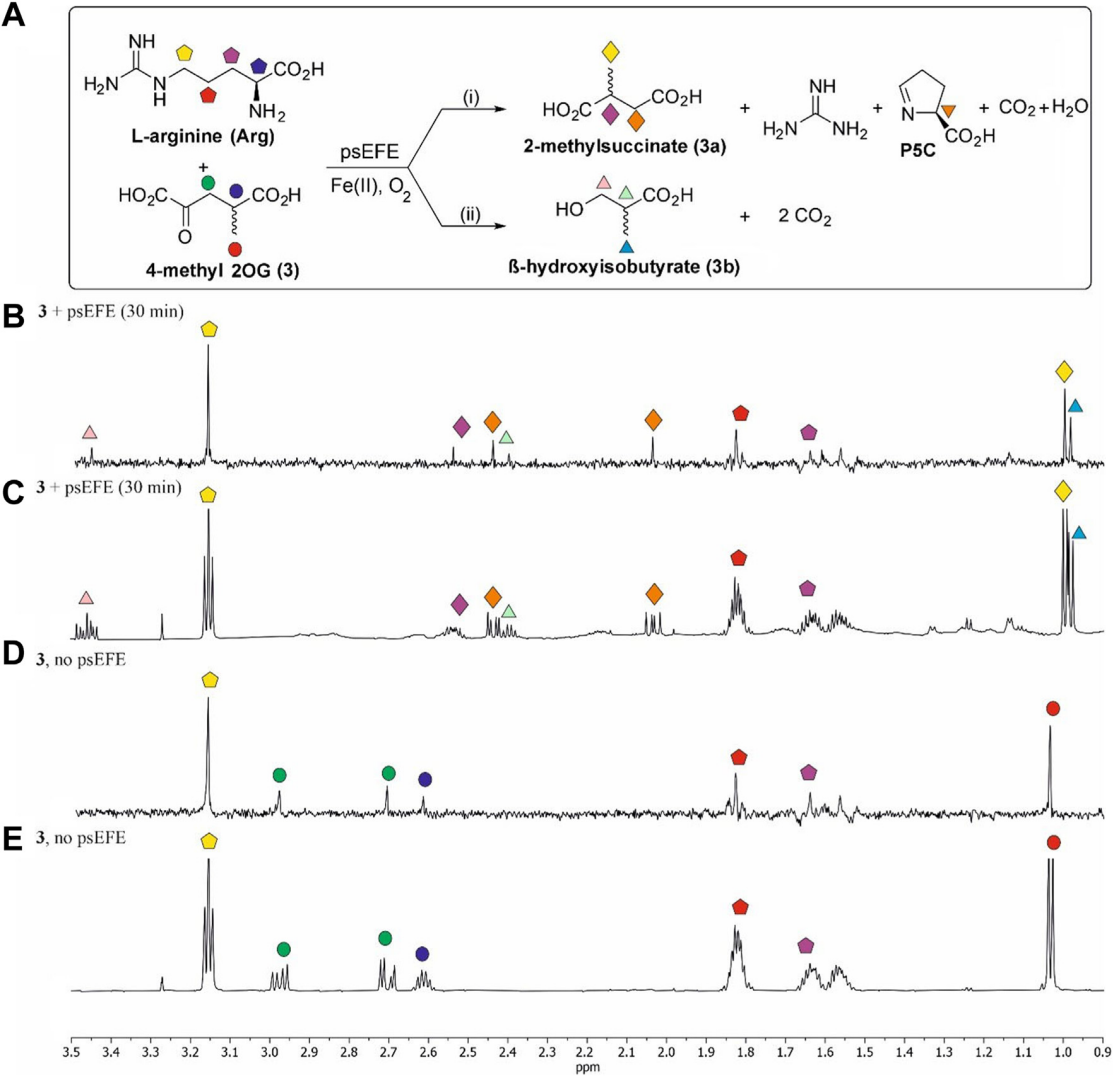
**Analysis of the reaction outcome of psEFE-catalyzed conversion of 4-methyl-2OG(3)using pure shift**^**1**^**H NMR.** (*A*) psEFE catalyzes the oxidation of 4-methyl-2OG (**3**) to give either (i) 2-methylsuccinate (**3a**) or (ii) β-hydroxyisobutyrate (**3b**); propene was not observed. Analysis (0.9–3.5 ppm) of the reaction of psEFE with **3** 30 min post addition of psEFE using (*B*) pure shift ^1^H NMR (700 MHz) (47, 48, 49, 50) and (*C*) ^1^H NMR (600 MHz) indicates that reaction products other than **3a**, **3b**, and P5C are not formed in substantial amounts under the tested conditions (Figs. S9–S12). (*D*, *E*) Analysis of the reaction mixture with **3** in the absence of psEFE using (*D*) pure shift ^1^H NMR (700 MHz) (47, 48, 49, 50) and (*E*) ^1^H NMR (600 MHz). **3** was used as a racemic mixture; it is possible that stereoselective conversion may occur. Note that the signals observed between ∼1.1 to ∼1.3 ppm in (*C*) originate from the psEFE sample. Note that for the pure shift ^1^H NMR analysis, the observed peak may not necessarily be at the center of the peak obtained when using standard NMR analysis (47, 48, 49, 50). Conditions: 400 μM **3**, 500 μM *L*-arginine, 500 μM *L*-ascorbate, 50 μM Fe (II), 800 μM TMSP-*d*_*4*_, and 10 μM psEFE in buffer (50 mM sodium phosphate, pH 7.4, 10%_v/v_ D_2_O).

The effects of 18 C3 and/or C4 substituted 2OG derivatives (22) on psEFE catalysis were investigated in the absence of 2OG; note that the chiral 2OG derivatives were used as racemates. Analysis of the reaction mixtures by ^1^H NMR reveals that psEFE does not catalyze the conversion of any of the tested 2OG derivatives to alkenes, at least within our limits of detection using ^1^H NMR (600 MHz) (Figs. 5; S9–S42). This observation is consistent with results reported during the course of our work, showing that, although psEFE employed 4-fluoro-2OG as a substrate to give fluoroethylene, it did not catalyze alkene formation from 2OG derivatives bearing alkyl or hydroxy substituents at the C4 position; note that alkene/diacid and alcohol/diacid product ratios were not reported, except for 4-hydroxy-2OG (**4**) (44). However, using IR spectroscopy-based assays, it was recently reported that psEFE catalyzes the formation of low levels of propylene from 4-methyl-2OG (**3**) (45). We did not accrue evidence for propylene formation from **3** or **10** by NMR under the tested conditions, even when using pure shift ^1^H NMR (48, 49, 50) (Figs. 6 and S42), which increases sensitivity for propene detection (47). The difference is likely a consequence of the different reaction conditions employed and/or the intrinsic alkene detection limitations of our ^1^H NMR-based psEFE assays.

Although psEFE did not catalyze the formation of alkenes from the tested C3 and/or C4 substituted 2OG derivatives, ^1^H NMR analyses of the reaction profiles >1 h post addition of psEFE reveal that 10 of the 18 tested 2OG derivatives were (co)substrates of psEFE, *i.e.*, 4-methyl-2OG (**3**), 4-hydroxy-2OG (**4**), 4-hydroxy-4-methyl-2OG (**5**), 4,4-dimethyl-2OG (**6**), 4-ethyl-2OG (**7**), 4-propyl-2OG (**8**), 4-butyl-2OG (**9**), 4-benzyl-2OG (**15**), 3-methyl-2OG (**10**), and the carbocycle **14** (Figs. 5; S9–S42). Interestingly, whilst psEFE catalyzed diacid formation from the 2OG derivatives **3** to **10**, **14**, and **15** in a manner apparently coupled to Arg (substrate) oxidation, as evidenced by P5C formation and by comparison with authentic samples of the corresponding diacid products, it only catalyzed alcohol formation from a subset of the identified substrates, *i.e.*, **3**-**10**, which thus mimic the bifurcating reactivity of 2OA (**2**) with psEFE (Figs. 4 and 5; S9–S42).

**Figure 7:**
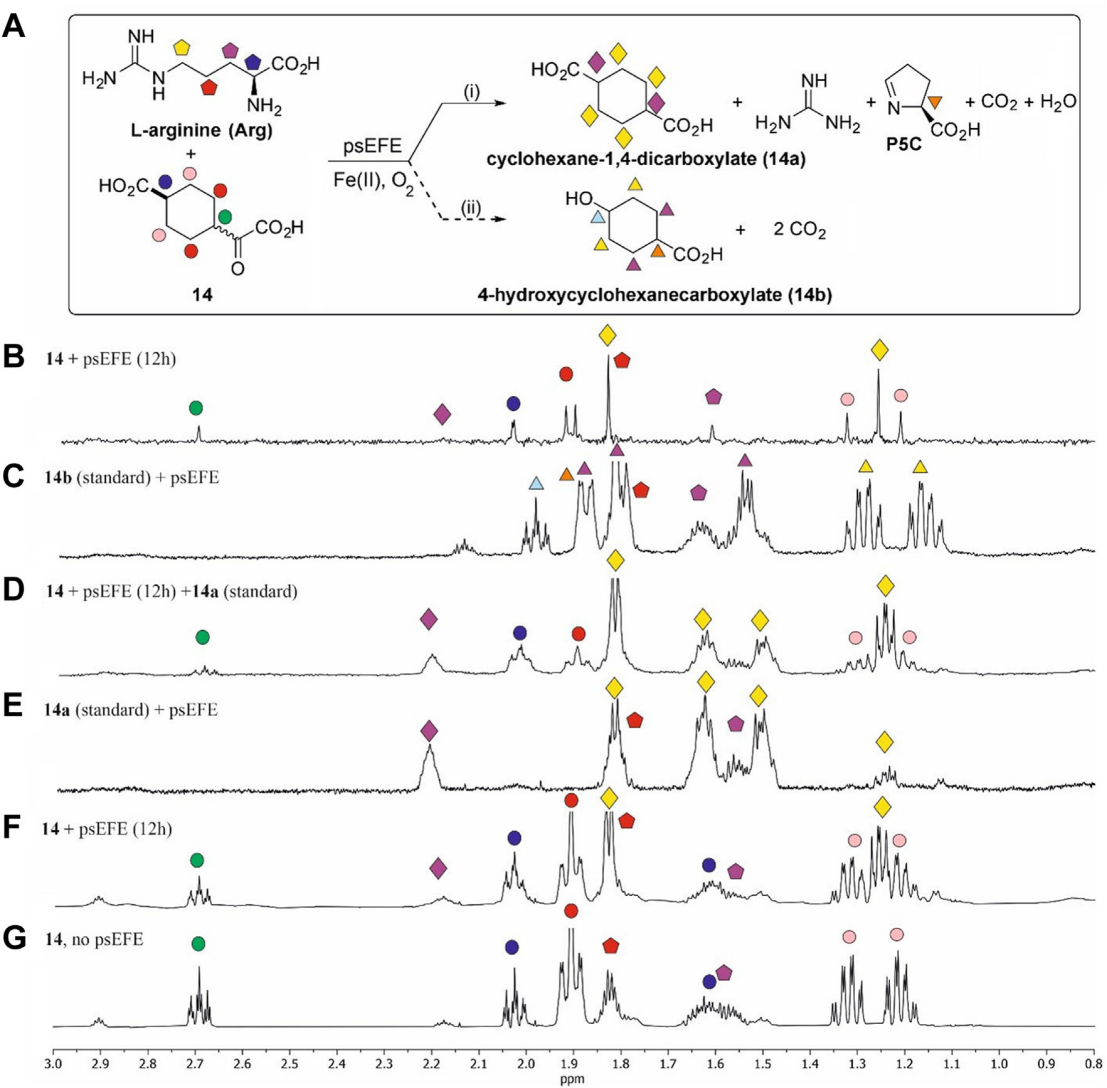
**psEFE employs carbocycle 14 as a cosubstrate to sustain Arg oxidation.** (*A*) psEFE catalyzes Arg oxidation using carbocycle **14** as a cosubstrate, but does not catalyze the competing decarboxylative β-oxidation of **14** to give **14b**. (*B*) analysis (0.8–3.0 ppm) of the crude reaction mixture by pure shift ^1^H NMR (700 MHz) (47, 48, 49, 50) 12 h post incubation with psEFE indicates that psEFE does not catalyze alcohol (**14b**) formation from **14**; (*C*–*G*) ^1^H NMR (600 MHz) analysis (0.8–3.0 ppm) of (*C*) 4-hydroxycyclohexanecarboxylate (**14b**, standard) in buffer, (*D*) a reaction mixture of psEFE and **14** spiked with cyclohexane-1,4-dicarboxylate (**14a**, standard) 12 h post addition of psEFE, (*E*) **14a** (standard) under standard conditions in the presence of psEFE; (*F*) a reaction mixture of psEFE and **14** 12 h post addition of psEFE; (*G*) **14** under standard conditions in the absence of psEFE. Conditions: 400 μM **14**, 500 μM L-arginine, 500 μM L-ascorbate, 50 μM Fe(II), 800 μM TMSP-*d*_*4*_, 30 μM psEFE, and, if appropriate, 2 mM of an appropriate authentic standard (50 mM sodium phosphate, pH 7.4, 10%_v/v_ D_2_O). **14** was used as a mixture of diastereomers, dr (*trans*:*cis*) ∼ 5:1; note it is possible that stereoselective conversion may occur. Note that for the pure shift ^1^H NMR analysis, the observed peak may not necessarily be at the center of the peak obtained when using standard NMR analysis (47, 48, 49, 50). A representative set of ^1^H NMR spectra from three independent repeats is shown (n = 3).

Although evidence for the psEFE-catalyzed formation of alcohol products from 4-butyl-2OG (**9**), 4-benzyl-2OG (**15**), and from the carbocycle **14** was not accrued under the tested conditions (Figs. 5 and 7), the levels of their psEFE-catalyzed conversion to the corresponding diacid products were relatively low and could not be quantified for **14** and **15**, because of signals overlapping in the ^1^H NMR which hampered accurate integration; diacid formation was supported by analysis of P5C formation and by the addition of the corresponding authentic synthetic diacid sample to the reaction mixture (Figs. 7; S42–S49). Formation of an apparent single diacid product for **9**, **14**, and **15** was supported using pure shift ^1^H NMR (700 MHz) analyses, which also revealed no evidence for formation of alcohol products (Figs. 7; S37 and S48).

All the chiral 2OG derivatives tested were racemic; it is possible that the absolute/relative configurations of the chiral derivatives, including the carbocycle **14**, may affect the efficiencies and outcomes of their reactions with psEFE. In this regard, it is of interest that psEFE catalyzed conversion of **14**, but not of those (bi)cyclic 2OG derivatives which have a more restricted conformational flexibility, *i.e.*, the 3- and 4-membered carbocycles **17** and **18**, as well as **19** and **20** which bear an aromatic or bicyclic carbon scaffold, respectively (Fig. 5). Likely, 2-oxoacids **17**−**20** cannot occupy a conformation which results in productive psEFE catalysis when bound to the active site, or they do not bind to the active site. The combined observations indicate that the substitution patterns of 2OG derivatives can alter the outcomes of their bifurcating reactions with psEFE.

To investigate the effect of the steric bulk of the 2OG C4 substituent on the outcome of the bifurcating psEFE reaction with 2OG derivatives, we analyzed the product ratios of diacid *versus* alcohol formation for the C4 alkyl-substituted 2OG derivatives (Fig. 5*A*; Table S1). The results reveal that substituting the methyl group of 4-methyl-2OG (**3**) for an ethyl group results in a ∼2-fold increase in the psEFE-catalyzed formation of the corresponding succinate derivative over the β-hydroxypropionate derivative from ∼1.8:1.0 to ∼3.2:1.0. Substituting the methyl group of **3** for a propyl group results in a ∼3-fold increase of the corresponding succinate derivative (∼4.1:1.0), while its substitution for bulkier butyl or benzyl groups completely suppresses formation of the potential alcohol product (Fig. 5*A*).

The comparison of the product ratio for 4-methyl-2OG (**3**) with those for 4,4-dimethyl-2OG (**6**) and 3-methyl-2OG (**10**) indicates that both the addition of a second methyl substituent at the C4 position and moving the methyl substituent to the C3 position favor formation of the alcohol products (Fig. 5; Table S1). 3-Ethyl-2OG (**11**), 3-propyl-2OG (**12**), 3-butyl-2OG (**13**), and 3-benzyl-2OG (**16**) do not react with psEFE under the tested conditions (even after prolonged incubation times, ∼20 h), by contrast with their C4 regioisomers **7**−**9** and **15** (Fig. 5). These results show that both the steric bulk and the position of the 2OG substituents affects the product ratio in psEFE catalysis. Note that, by contrast with the observed pH dependence of the product ratio when using 2OG as a (co)substrate (Fig. 2), altering the pH from 6.5 to 8.5 did not affect the product ratio of psEFE catalysis using 2OG derivatives **3** and **10** (Figs. S57 and S58).

In addition to analyzing the product ratios, we analyzed the efficiencies of product formation and the overall levels of conversion at ∼1.5 h post-addition of psEFE for the psEFE-catalyzed reaction of 4-methyl-2OG (**3**), 4-ethyl-2OG (**7**), 4-propyl-2OG (**8**), and 4-butyl-2OG (**9**) (Fig. 4, Table S1). The results reveal that the specific activities of the 2OG derivatives with psEFE (*i.e.*, the efficiency of catalysis) decreases with increasing steric bulk of the C4 substituent: substrate conversion was >95% for 2OG, **3**, and **7**, but decreased to ∼70% for **8**, and to ∼50% for **9**. The reduced efficiency of conversion correlates with the observed trend in the product ratios, which favors diacid formation coupled to Arg oxidation for sterically bulky C4 substituents (Fig. 5, Table S1). The levels of conversion of **8** and **9** did not increase after >20 h incubation, an observation which may indicate (at least for **9**) that the absolute configuration of the C4 stereocenter affects the efficiency and/or the outcome of the reaction of 2OG derivatives with psEFE, because the substrates used were racemic.

The combined evidence implies that the steric bulk of the 2OG substituents not only affects the product ratio, but also the overall psEFE efficiency, potentially due to less efficient binding at the active site and/or their reduced conformational flexibility. This observation implies that active site mutagenesis may alter the reactivity of psEFE active site variants with 2OG derivatives, as precedented by studies on the reactivity of C4 substituted 2OG derivatives with active site variants of the JmjC lysine-specific *N*^ε^-demethylases KDM4A (25); this proposal is validated by a recent proof-of-concept study on the reactivity of the L206V psEFE active site variant which catalyzed propene formation from (4*R*)-4-methyl-2OG more efficiently than wt psEFE (45).

We next compared the diacid:alcohol ratios obtained for 4-methyl-2OG (**3**), 4-hydroxy-2OG (**4**), 4-hydroxy-4-methyl-2OG (**5**), and 4,4-dimethyl-2OG (**6**) to investigate whether, in addition to the steric bulk of the 2OG C4 substituent, the nature of the substituent affects product ratios. The results imply that substituting the C4 methyl group of **3** with a hydroxy group does not substantially affect either the diacid:alcohol ratio or the extent of conversion after 1 h incubations (Figs. 4 and 5). Note that the observed diacid:alcohol ratio of **4** was ∼2:1 in our ^1^H NMR assay (Table S1), whereas it was ∼3:1 in reported LC-MS assays (44). Similarly, substituting one of the two methyl groups of **6** for a hydroxy group did not affect the product ratio, within the limits of detection (Fig. 4), but reduced the overall levels of conversion from ∼80% to ∼30% over the analyzed time period (Fig. 4, Table S1).

## Discussion

The *P. savastanoi* (previously: *P. syringae*) pv. phaseolicola ethylene-forming enzyme (psEFE) (35, 36) is of interest from both biocatalytic and mechanistic perspectives. When using 2OG (**1**) as a (co)substrate, its reaction bifurcates resulting in two types of oxidative decarboxylation reactions, *i.e.* (i) a Grob-type fragmentation of 2OG to give ethylene and three molecules of CO_2_, and (ii) the oxidation of 2OG to give succinate (and CO_2_) coupled to Arg oxidation giving guanidine and P5C (Fig. 1) (37, 38, 40, 41). It is also reported that psEFE can catalyze the oxidation of some C4 substituted 2OG derivatives to give the corresponding β-hydroxypropionate derivatives (Fig. 5) (44). It is desirable to understand the factors which govern the product ratio for the (co)substrates of psEFE, *inter alia*: (i) to improve psEFE catalysis for biocatalytic applications, including ethylene production from renewable resources, (ii) to predict the reactivity of other 2OG oxygenases with 2OG derivatives, and (iii) to inform on the, likely context-dependent, biological roles of psEFE.

The biological functions of ethylene in plants are, at least in part, understood, whereas the reasons why plant pathogens, including *P. savastanoi*, synthesize ethylene are less well characterized (52). Bacteria may have evolved to produce ethylene to interact with plant ethylene receptors in an ACC-independent manner (53, 54), *e.g.*, to perturb the plant metabolism and/or immune response. *P. savastanoi* employs 2OG rather than ACC as an ethylene precursor; although psEFE and ACCO are structurally related, their mechanisms are different (Fig. 1) (11). To our knowledge, no 2OG oxygenase other than psEFE (and analogous EFEs in related bacteria) is reported to produce ethylene from 2OG, including the bacterial 2OG oxygenases VioC and OrfP which also catalyze hydroxylation of isolated arginine, however, unlike psEFE, at the arginine C3 position (55, 56).

PsEFE catalysis has potential to directly and indirectly regulate cellular levels of 2-oxoacids and associated metabolites, including α-amino acids. The psEFE-catalyzed C5 hydroxylation of Arg to give P5C provides a further link to 2-oxoacid/amino acid metabolism, because P5C, which is in equilibrium with glutamate-5-semialdehyde, is metabolically linked to glutamate, proline, and ornithine (57). P5C is a substrate of various bacterial and plant enzymes, though above threshold levels is reported to be cytotoxic (58, 59, 60, 61, 62). The physiological roles of guanidine formation following arginine C5 hydroxylation are unclear, but recent evidence suggests that guanidine is a plant metabolite suggesting that psEFE catalysis may perturb plant guanidine levels (63). It has also been shown that specific 2OG-dependent oxygenases in plants and algae catalyze C6 hydroxylation of homoarginine, resulting in spontaneous guanidine formation; however, these enzymes do not catalyze ethylene formation. Interestingly, despite the homoarginine hydroxylase and psEFE catalyzing closely related reactions, their sequences show only ∼25% similarity (63).

Our NMR studies on the (co)substrate scope of psEFE validate prior studies using different methods showing that the lysine metabolite 2OA (**2**) is an efficient substrate of psEFE in the absence of 2OG (39, 44). Pseudomonal 2OG oxygenases other than psEFE are also reported to employ **2** as a cosubstrate, however, with variable efficiencies compared to 2OG. For example, catalysis of the *Pseudomonas putida* S-313 oxygenative alkylsulfatase with **2** as a cosubstrate was ∼20% less efficient than with 2OG (17), catalysis of the *Pseudomonas stutzeri* WM88 hypophosphite:2-oxoglutarate dioxygenase (HtxA) with **2** was ∼70% less efficient than with 2OG (18), whereas catalysis of *Pseudomonas aeruginosa* γ-butyrobetaine hydroxylase (BBOX) with **2** did not proceed (64). The combined observations thus suggest that increased **2** levels may affect catalysis of Pseudomonal 2OG oxygenases in an, at least, partly selective manner. It is possible that **2** or, by implication, other 2OG derivatives may be the preferred (co)substrate of some 2OG oxygenases or that competition between 2OG and **2** regulates catalysis in cells (22, 23).

**Figure 8:**
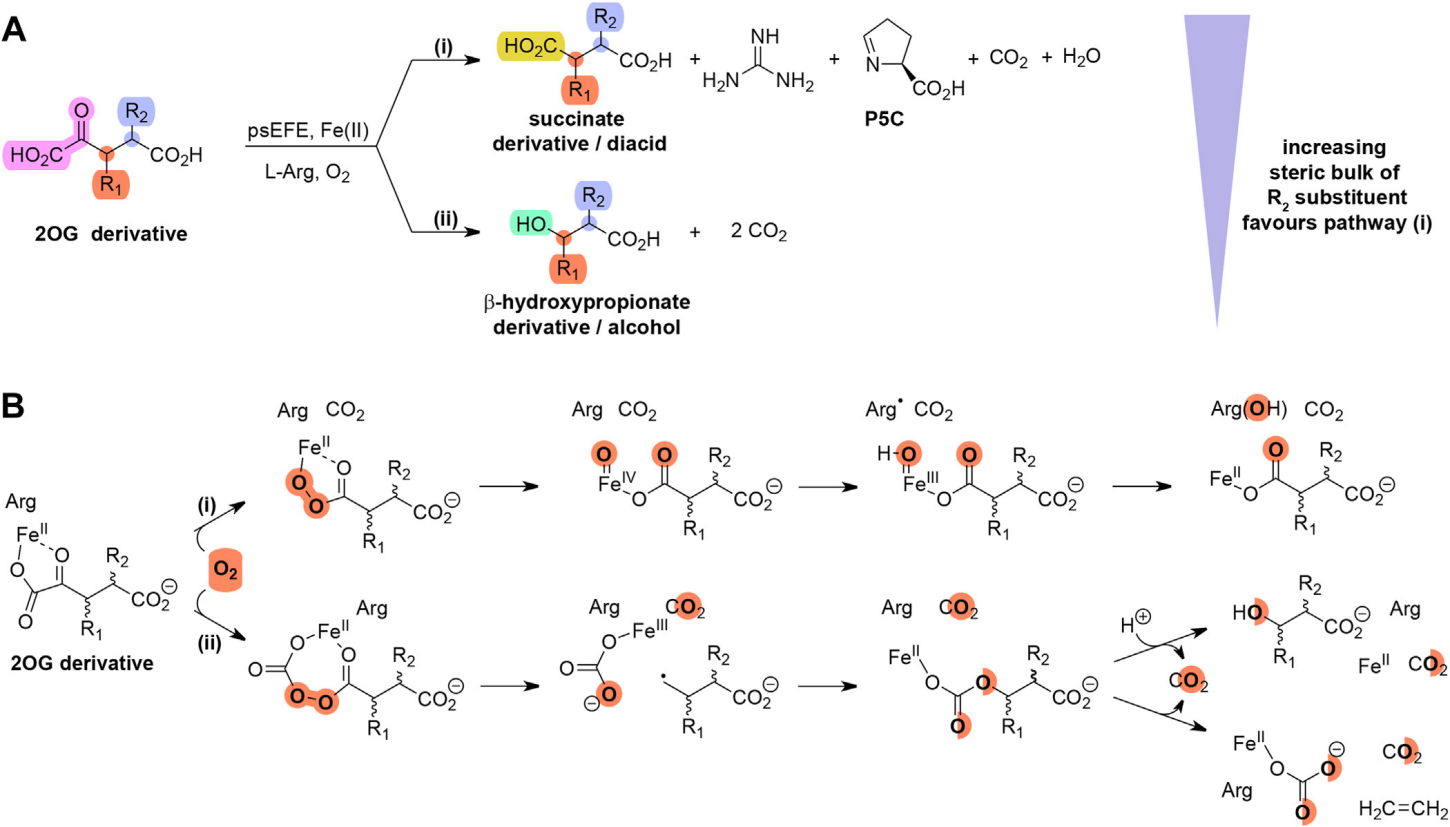
**2OG derivatization influences the outcome of psEFE catalysis.** (*A*) C3 and/or C4 substituted 2OG derivatives can be (co)substrates of psEFE in a bifurcating reaction: (i) to catalyze Arg hydroxylation resulting in formation of P5C and the corresponding succinate derivative and/or (ii) to give a β-hydroxypropionate derivative/alcohol. None of the tested 2OG derivatives manifested alkene formation, but differences in the ratios of P5C/succinate derivative formation *versus* β-hydroxypropionate derivative formation were observed. Increasing the steric bulk of the C3 substituent (R_1_) beyond a methyl group apparently ablates psEFE catalysis. Increasing the steric bulk of the C4 substituent (R_2_) shifts the product ratio to favor P5C/succinate derivative formation over β-hydroxypropionate derivative formation, and reduces overall levels of catalysis. Cyclic 2OG derivatives (*i.e.*, linking R_1_ and R_2_) either were inactive (**17**–**20**) or only catalyzed P5C/diacid formation (**14**); (*B*) proposed outline mechanism of psEFE (11, 44).

Our observations that psEFE accepts C3 and/or C4 substituted 2OG derivatives as (co)substrates in addition to 2OA, complements recently reported studies on psEFE substrate scope using five C4 substituted 2OG derivatives (Fig. 5) (44). Notably, both increasing the steric bulk of the 2OG C4 substituent and using conformationally rigid carbocyclic 2OG derivatives apparently shifts the product ratio of psEFE catalysis in favor of or completely to Arg hydroxylation/diacid formation over β-hydroxypropionate derivative/alcohol formation (Figs. 5 and 8).

The bifurcating reaction of psEFE with 2OG derivatives as prime substrates to give variable product ratios is precedented by studies on the mechanistically distinct iron-dependent cytochrome P450 peroxidase OleT_JE_ (CYP152L1) that catalyzes formation of both alcohol and terminal alkene products *via* oxidation of fatty acids (65, 66). Notably, as proposed for psEFE (44), the bifurcating reactivity of OleT_JE_ is reported to be a result of substrate-centered radical intermediates (67, 68, 69, 70), the reaction fate of which can differ between reaction with an iron-bound/carbonate oxygen atom to give an alcohol and β-decarboxylation to give an alkene. The observed variations in product ratios may thus reflect different stabilities of radical intermediates and/or restricted conformations of intermediates favoring particular reaction outcomes.

It is of interest that several of the alternative psEFE substrates identified here are natural products, *i.e.*, 4-methyl-2OG (**3**) (71, 72, 73, 74), 4-hydroxy-2OG (**4**) (75, 76), 4-hydroxy-4-methyl-2OG (**5**) (77, 78, 79), and 3-methyl-2OG (**10**) (80), some of which have been detected in *Pseudomonas* species (81, 82), implying that the primary or sole biological role of psEFE catalysis may not be ethylene production. psEFE catalyzes the conversion of the natural product 3-methyl-2OG (**10**) (80) to give β-hydroxybutyrate (**10b**), which is a reported metabolite of *Pseudomonas* species (83) and which is a building block in the biosynthesis of the biodegradable polyester poly(3-hydroxybutyrate) (84). It is possible that the reaction of psEFE with 2-oxoacids other than 2OG could help *P. savastanoi* sense and/or adapt to internal and/or external stimuli, possibly in a context-dependent manner based on (co)substrate availability. There is precedent for roles of 2OG oxygenases and ACCO to act in a sensing capacity, in particular in terms of regulating the response to hypoxia in animals and plants, respectively (1, 10, 85).

The apparent (co)substrate promiscuity of psEFE is precedented, *e.g.*, by work on (i) the cosubstrate scope of human 2OG oxygenases which revealed that C3 and/or C4 substituted 2OG derivatives can selectively sustain and/or inhibit catalysis by the protein hydroxylases AspH, FIH, and JMJD5 (22, 23, 24), (ii) plant ACCOs, which employ a range of ACC derivatives to give products other than ethylene (86, 87, 88), and (iii) transaminases and oxidoreductases, which are mechanistically and structurally distinct from 2OG oxygenases but some of which employ 2OG as a substrate (89, 90, 91). The apparent ability of psEFE to accept 2OG derivatives with a range of C3 and/or C4 substituents as (co)substrates (Fig. 5), contrasts with its reported relatively narrow scope of accepting ‘prime’ substrates other than Arg (39, 40, 92). These observations indicate that the fragmentation of Arg itself may be of biological importance (see above). In apparent contrast with psEFE, both human AspH and FIH not only accept a diverse set of 2OG derivatives as cosubstrate (22, 23), but also catalyze oxidation of multiple protein substrates and catalyze different types of oxidation (28, 30, 34, 93, 94).

The biocatalytic production of ethylene from renewable resources is desirable from a sustainability perspective (42, 43). However, the potential of psEFE for biocatalytic ethylene production on an industrial scale is in part currently compromised by the bifurcating reaction of 2OG, as ethylene formation competes with succinate formation, a process coupled with arginine hydroxylation (Fig. 1) (95). Our results showing that the ratio of ethylene *versus* succinate formation is affected by the reaction conditions are thus of interest, in particular the observation that more acidic conditions (*i.e.*, pH 6.5) favor ethylene over succinate production (Fig. 2). The reasons for the observed dependance of the ethylene:succinate ratio on the pH are currently unclear and may relate to different protonation states of residues proximate the 2OG binding site. Refining the residues involved in 2OG and/or Arg binding may help in research aimed at engineering psEFE in a manner to enhance ethylene production (51).

The observation that psEFE catalyzes formation of alcohol products from some of the tested C3 and, in particular, C4 substituted 2OG derivatives highlights the potential of psEFE biocatalytic applications beyond ethylene formation. For example, psEFE catalyzes the formation of β-hydroxybutyrate from the natural product 3-methyl-2OG (**10**) (80), affording a useful building block for the industrial synthesis of biodegradable polymers (84). The psEFE-catalyzed decarboxylative β-oxidation of 2OG derivatives to give alcohol products complements prior work on the utility of 2OG derivatives for the biocatalytic synthesis of alcohols, which revealed that oxidoreductases can catalyze the reduction of 2OG derivatives to give the corresponding hydroxyglutarate derivatives (91).

## Experimental procedures

### Chemicals

2-Oxoglutarate (2OG), DL-4-hydroxy-2-oxoglutarate (4-OH-2OG, **4**), and 2-oxoadipate (2OA, **2**) were commercially-sourced (Sigma Aldrich Inc), all the other tested C3 and/or C4-substituted 2OG derivatives were synthesized as racemic mixtures as reported (22). All other chemicals used for this work were commercially-sourced (Sigma Aldrich Inc, Fluorochem Ltd) and used without further purification.

### psEFE production and purification

Isolated recombinant psEFE was produced and purified as reported (40). A pETite N-His SUMO Kan plasmid encoding for *P. savastanoi* (previously: *P. syringae*) pv. phaseolicola ethylene/succinate-forming enzyme (psEFE; EC:1.13.12.19; UNIPROT ID: P32021) (40), codon optimized for expression in *Escherichia coli*, was transformed into competent T7 Express LysY competent *E. coli* cells (New England Biolabs). Cells were grown in 2TY media supplemented with kanamycin (30 μg/ml) at 37 °C with agitation (180 rpm), until psEFE expression was induced at an OD_600_ ∼0.8 by addition of isopropyl-β-D-thiogalactopyranoside (final concentration: 0.5 mM). Cells were incubated at 18 °C for 16 h, before being harvested by centrifugation (11,395*g*, 15 min, 4 °C); the resultant cell pellet was stored at −80 °C.

The frozen cells were resuspended in ice-cold buffer (50 mM Tris, pH 7.8, 500 mM NaCl, 20 mM imidazole) supplemented with DNase I (bovine pancreas, Sigma-Aldrich) and lysed at 4 °C using a high-pressure cell disrupter (Continuous Flow Cell Disruptor CF1; Constant Systems Ltd) followed by centrifugation (58,545*g*, 30 min, 4 °C). The supernatant was purified at 4 °C using Ni(II)-affinity chromatography (HisTrap HP column, GE Healthcare) using an ÄKTA Pure chromatography system (GE Healthcare) employing an elution gradient with increasing buffer (50 mM Tris, pH 7.8, 500 mM NaCl) imidazole concentration (from 20 mM to 500 mM imidazole). Fractions containing psEFE were combined, concentrated using Amicon Ultra centrifugal filters (3082*g*, 4 °C), and buffer exchanged using a PD-10 desalting column (Cytiva) to remove imidazole (25 mM Tris, pH 7.8, 500 mM NaCl, 10%_v/v_ glycerol). The N-terminal His_6_-SUMO tag was cleaved overnight using sentrin-specific protease 2 (SenP2; Fig. S59) at 4 °C; and the resultant mixture was purified using Ni(II)-affinity chromatography (50 mM Tris, pH 7.8, 500 mM NaCl, 20 mM imidazole). The flow-through was collected, concentrated, and purified using size-exclusion chromatography (300 ml HiLoad 26/60 Superdex 75 pg column; ÄKTA Pure chromatography system; 25 mM Tris, pH 7.8, 100 mM NaCl; 1 ml/min). Eluted fractions containing >95% pure psEFE (determined by SDS-PAGE analysis) were pooled, then concentrated using Amicon Ultra centrifugal filters (3082*g*, 4 °C) and aliquoted; psEFE purity was also confirmed by LC-MS and had the anticipated mass, *i.e.*, 39,369.5 Da (calculated mass: 39,370.5 Da). The aliquots were flash frozen in liquid N_2_ and stored at −80 °C. The psEFE concentration was determined using a NanoDrop One microvolume UV-Vis spectrophotometer (Thermo Scientific), assuming that the concentration of active psEFE equals the total psEFE concentration (note that potent covalent or tight-binding psEFE inhibitors suitable for active site titrations are not yet reported); fresh aliquots of psEFE were used for all biochemical experiments.

### ^1^H NMR assays

Aqueous stock solutions of L-arginine (Arg; 50 mM), 2OG (**1**) or a 2OG derivatives (40 mM), L-ascorbate (LAA; 50 mM), and ferrous ammonium sulfate (FAS; 100 mM) were freshly prepared the day the experiment was performed. These stock solutions, together with a solution (100 mM) of (trimethylsilyl)propionic-2,2,3,3-*d*_*4*_ acid sodium salt (TMSP-*d*_*4*_) (46), which was used as an internal standard (−0.11 ppm), were diluted in buffer (50 mM sodium phosphate, 10%_v/v_ D_2_O, pH 7.4) to give Arg (5 mM), 2OG/2OG derivative (4 mM), LAA (5 mM), FAS (0.5 mM), and TMSP-*d*_*4*_ (8 mM) solutions for use in the NMR assays. ^1^H NMR (600 MHz) assays were performed using a Bruker AVIII 600 instrument with a Prodigy nitrogen broadband cryoprobe. Pure shift ^1^H NMR (700 MHz) analyses were performed as reported (47), using a Bruker AVIII 700 MHz NMR spectrometer with a 5-mm inverse triple-resonance-inverse cryoprobe.

Assays were performed in a reaction volume of 180 μL with the following final concentrations: 500 μM L-arginine, 400 μM L-ascorbate, 400 μM co-substrate, 50 μM Fe(II), and 2, 10 or 30 μM psEFE. The indicated stock solutions were strictly added in the following order into a 1.5 mL Eppendorf tube: aqueous buffer (50 mM sodium phosphate, 10%_v/v_ D_2_O, pH 7.4; variable volume), then Arg (5 mM; 18 μl), then LAA (5 mM; 18 μL), then 2OG/2OG derivative (4 mM; 18 μL), then FAS (0.5 mM; 18 μL), then TMSP-*d*_*4*_ (8 mM; 18 μL), then psEFE (variable volume). The assay mixture was carefully mixed with a glass Pasteur pipette, transferred to a 3 mm Bruker NMR tube, and hand centrifuged.

The NMR tubes were transferred as rapidly as possible to the spectrometer sample exchanger and inserted into the probe of a Bruker AVIII 600 instrument. The probe was locked to D_2_O (10%_v/v_ in water), followed by auto-tuning (‘atma’ command in Bruker TopSpin 4.3.1). The sample was then shimmed (tshim), receiver gain was adjusted (rga), and consecutive acquisitions were started. The variable delay was set to zero and the number of scans per acquisition was set to 64 scans per acquisition to ensure a sufficient signal-to-noise ratio; the relaxation delay was minimized to reduce the time-gap between acquisitions. For ^1^H experiments, water suppression was achieved using perfect echo-modified WATERGATE experiments (96). Digital resolution of the free induction delay (FID) was adjusted to 0.3 Hz for accuracy in integration. Peak integrals were normalized *via* comparison with that of TMSP-*d*_*4*_. Assigned products were validated by spiking the reactions 12 to 24 h post psEFE addition with 5 μL of a freshly prepared 40 mM stock solution (in: 50 mM sodium phosphate, 10%_v/v_ D_2_O, pH 7.4) of the relevant commercially-sourced authentic standards.

Spectra were phased and corrected automatically for peak symmetry and baseline correction. Integral precision was attenuated by zero filling which adjusted the size of the spectra to the number of data points used. Spectra were integrated using MestReNova x86_64 (Mestrelab research) and integrals were calibrated and scaled to the internal standard TMSP-*d*_*4*_. Data was plotted using GraphPad Prism 9. All time course data was obtained in independent triplicates.

## Data availability

All data is contained within the manuscript and Supporting Information.

## Supporting information

This article contains supporting information (22, 46, 47, 48, 49, 50, 96, 97).

## Conflict of interest

The authors declare that they have no conflicts of interest with the contents of this article.
