# Supplementary material for: Substitution of 2-oxoglutarate alters reaction outcomes of the Pseudomonas savastanoi ethylene-forming enzyme
Source: J Biol Chem. 2024 Jul 9;300(8):107546. doi: 10.1016/j.jbc.2024.107546 (PMC11345546; doi:10.1016/j.jbc.2024.107546)
Supplement: Supporting Information [file mmc1.pdf]

Substitution of 2-oxoglutarate alters reaction outcomes of the *Pseudomonas savastanoi* ethylene-forming enzyme

Siddhant Dhingra[1], Zhihong Zhang[1], Christopher T. Lohans[1,2], Lennart Brewitz[1,*], and Christopher J. Schofield[1,*]

[1]Chemistry Research Laboratory, Department of Chemistry and the Ineos Oxford Institute for Antimicrobial Research, University of Oxford, 12 Mansfield Road, OX1 3TA, Oxford, United Kingdom.

[2]Current address: Department of Biomedical and Molecular Sciences, Queen's University, Kingston, Ontario, K7L 3N6, Canada.

*Email: christopher.schofield@chem.ox.ac.uk or lennart.brewitz@chem.ox.ac.uk

Table of contents

1. General Information S2
2. Supporting Figures S3-S61
3. Supporting Tables S62-S63
4. References S63

1. General Information

^{1}H NMR assays (600 MHz) were performed using a Bruker AVIII 600 instrument with a Prodigy nitrogen broadband cryoprobe, Pure shift ^{1}H NMR (700 MHz) analysis of reaction mixtures was performed as described (1) using a Bruker AVIII 700 MHz NMR spectrometer equipped with a 5-mm inverse triple-resonance-inverse cryoprobe. 4-Hydroxy-2OG (**4**) was obtained as a racemate from Merck UK Ltd; all other chiral 2OG derivatives were prepared as racemates as reported (2).

For ^{1}H NMR experiments, water suppression at ~4.7 ppm was achieved using the perfect echo-modified WATERGATE method (3). 3-(Trimethylsilyl)-2,2,3,3-tetradeuteropropionic acid (4) (TMSP-d_4) was used as an internal standard (–0.11 ppm) to enable product quantification via comparison of peak integrals with that of TMSP-d_4. The stereochemistry of P5C was not assigned, note that it may epimerize under the assay conditions.

Symbols are used to annotate signals in the ^{1}H NMR spectra as shown below:

**Annotation**	**Molecule(s)**
squares	L-ascorbate (LAA)
pentagons	L-arginine (Arg)
inverted triangle	L-Δ^1-pyrroline-5-carboxylate (P5C)
circles	2OG/2OG derivatives
diamonds/triangles	psEFE reaction products

Colors are used to distinguish between chemically distinct protons, as shown below:

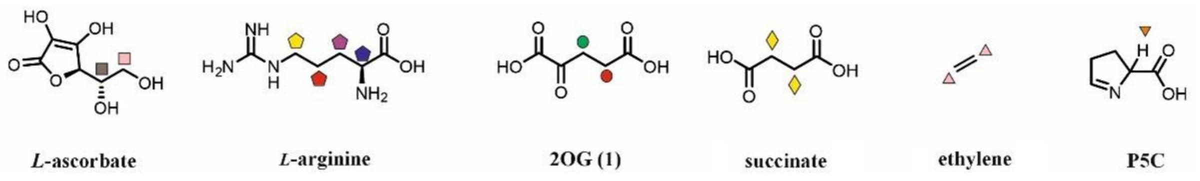

2. Supporting Figures

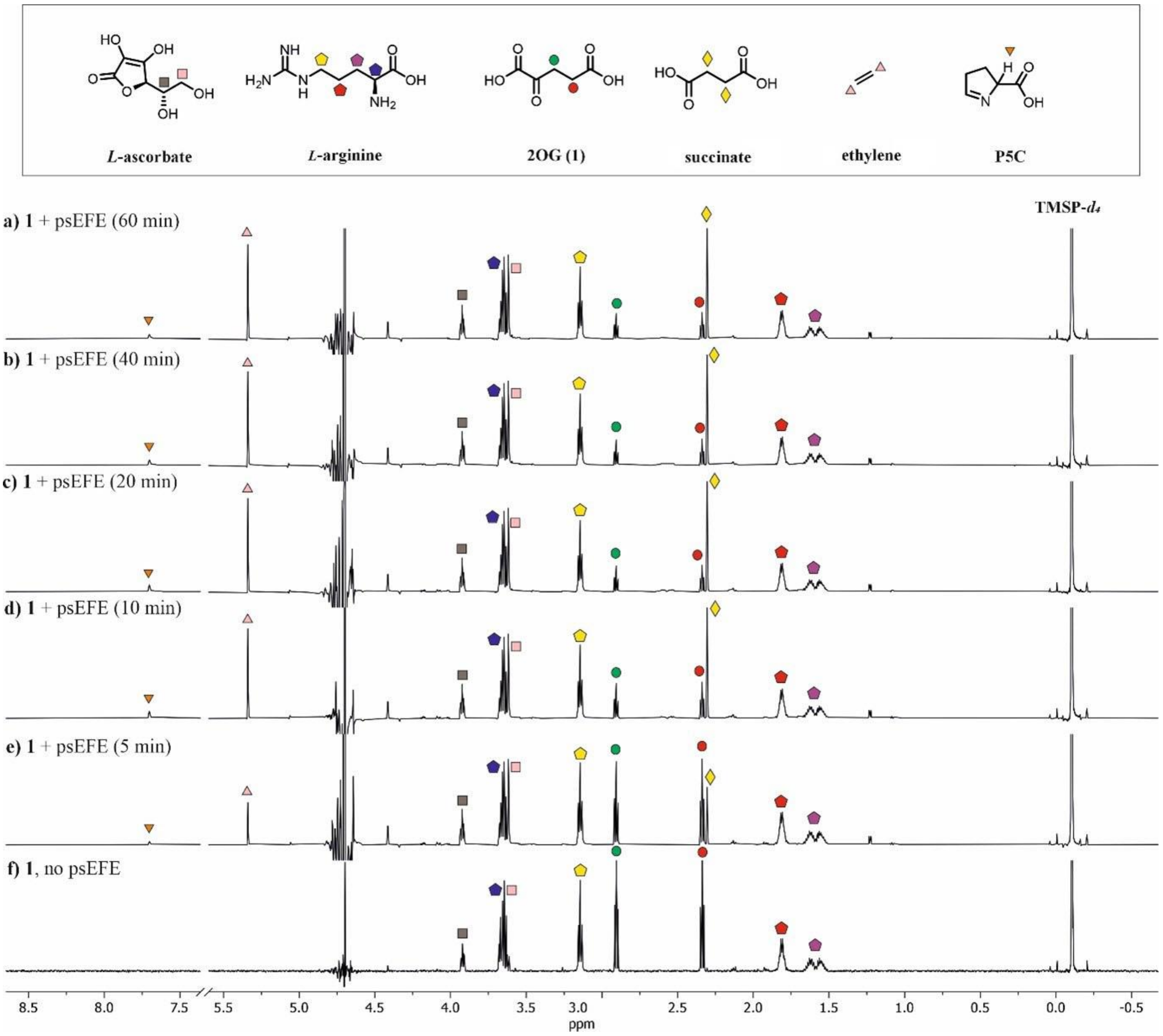

Supporting Figure S1. ^{1}H NMR analysis of psEFE catalysis using 2-oxoglutarate (2OG) as a cosubstrate under optimized assay conditions. a-e) ^{1}H NMR analysis of the reaction of psEFE with 2OG (**1**) after: **a**) 60 min, **b**) 40 min, **c**) 20 min, **d**) 10 min, and **e**) 5 min. **f**) ^{1}H NMR analysis of the reaction mixture in the absence of psEFE. Conditions: 400 µM 2OG (**1**), 500 µM *L*-arginine, 500 µM *L*-ascorbate, 50 µM Fe(II), 800 µM TMSP-d_4 (4), and 2 µM psEFE in buffer (50 mM sodium phosphate, pH 7.4, 10%$_{v/v}$ D_2O). Both the doublet observed at ~1.2 ppm and the singlet observed at ~4.4 ppm originate from the enzyme sample; both signals were observed in the enzyme-only control.

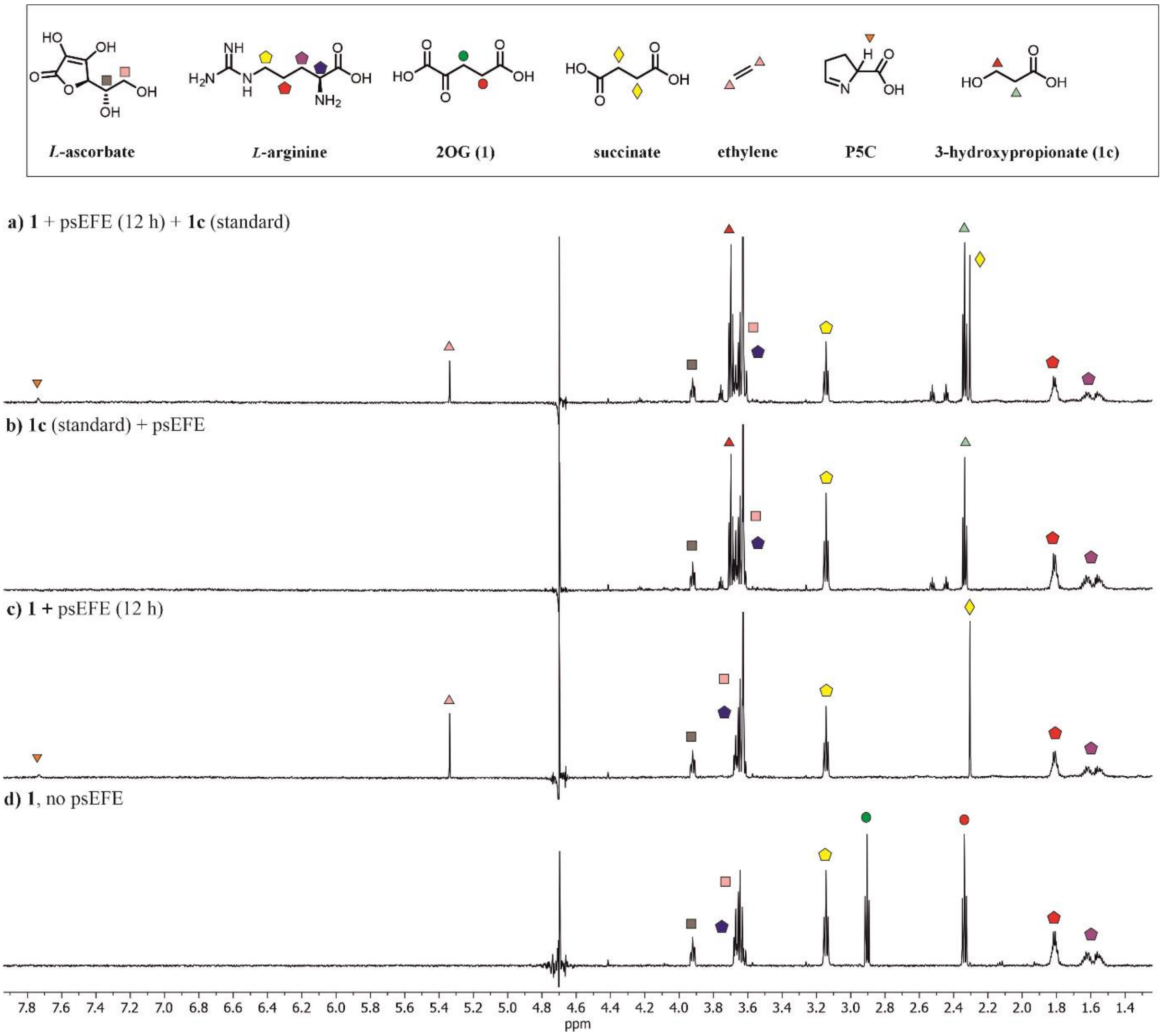

Supporting Figure S2. ^{1}H NMR analysis implies that 3-hydroxypropionate (1c) is not a product of psEFE catalysis using 2OG (1) and L-Arg as substrates. a) ^{1}H NMR analysis of the reaction of psEFE with 2OG (**1**) and L-Arg spiked with a synthetic standard of 3-hydroxypropionate (**1c**) indicates that ethylene (5.35 ppm), succinate (2.32 ppm), and P5C (7.70 ppm) are products, no evidence for 3-hydroxypropionate (**1c**) was observed; **b**) ^{1}H NMR spectrum of synthetic 3-hydroxypropionate (**1c**) under standard conditions in the absence of psEFE; **c**) ^{1}H NMR analysis of the reaction of psEFE with 2OG (**1**) 12 h post addition of psEFE; **d**) ^{1}H NMR spectrum of 2OG (**1**) under standard conditions in the absence of psEFE. Conditions: 400 µM 2OG (**1**), 500 µM *L*-arginine, 500 µM *L*-ascorbate, 50 µM Fe(II), 800 µM TMSP-d_4, 2 µM psEFE, and, if appropriate, 500 µM of the appropriate authentic standard in buffer (50 mM sodium phosphate, pH 7.4, 10%$_{v/v}$ D_2O). The singlet observed at ~4.4 ppm originates from the enzyme sample; it was observed in the enzyme-only control. The two low level triplets at ~ 2.4 and ~ 2.5 ppm originate from the commercially obtained sample of **1c**.

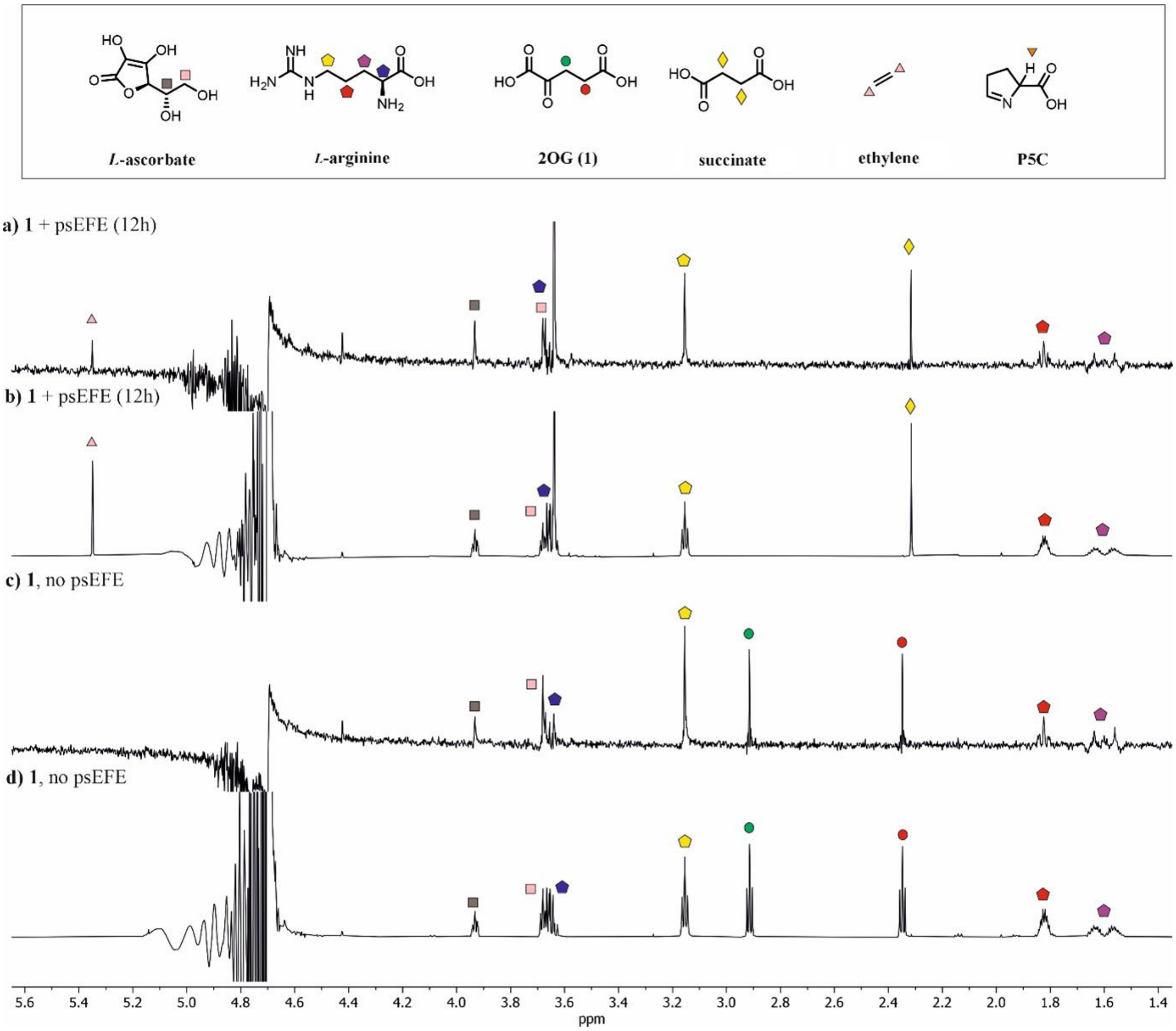

Supporting Figure S3. Analysis of the outcome of the reaction of psEFE with 2OG (1) using pure shift ^{1}H NMR. Analysis of the reaction of psEFE with 2OG (**1**) 1 h post addition of psEFE using: **a**) pure shift ^{1}H NMR (1, 5-7) and **b**) ^{1}H NMR. The results indicate that substantial amounts of reaction products other than ethylene, succinate, and P5C (Supporting Figures S1 and S2) are not formed under the tested conditions. Analysis of the reaction mixture with 2OG (**1**) in the absence of psEFE using: **c**) pure shift ^{1}H NMR (1, 5-7) and **d**) ^{1}H NMR. Conditions: 400 µM 2OG (**1**), 500 µM *L*-arginine, 500 µM *L*-ascorbate, 50 µM Fe(II), 800 µM TMSP-d_4, and 2 µM psEFE in buffer (50 mM sodium phosphate, pH 7.4, 10%$_{v/v}$ D_2O). Note that for the pure shift ^{1}H NMR analysis, the observed peak may not necessarily be at the center of the peak obtained when using standard NMR analysis (1, 5-7). The singlet observed at ~4.4 ppm originates from the enzyme sample; it was observed in the enzyme-only control.

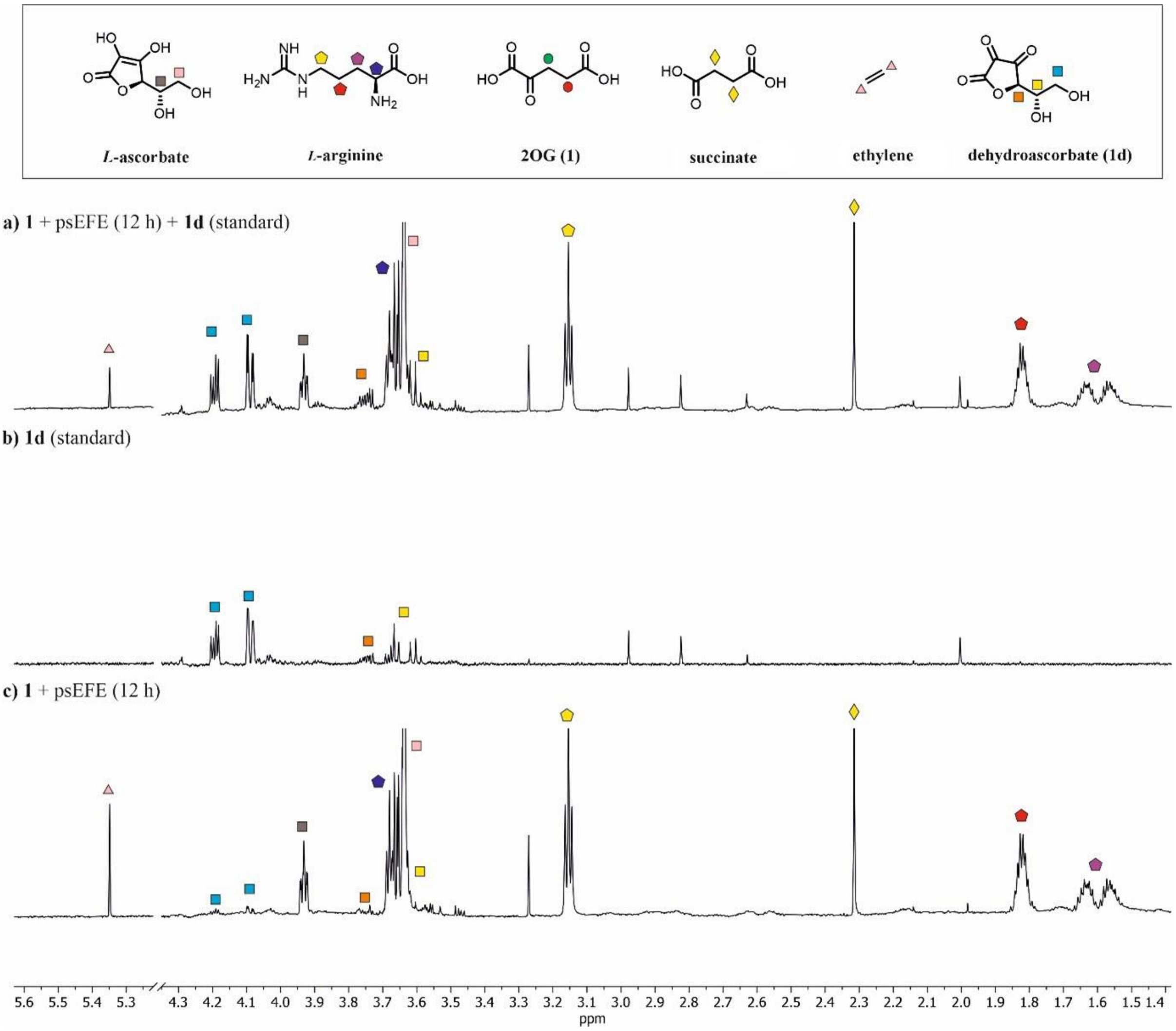

Supporting Figure S4. Low levels of dehydroascorbate (1d) are formed by the oxidation of L-ascorbate during psEFE catalysis. a) ^{1}H NMR analysis of the reaction of psEFE with 2OG (**1**) spiked with synthetic dehydroascorbate indicates that low levels of dehydroascorbate (**1d**) are present in the reaction mixture; **b**) ^{1}H NMR spectrum of **1d** in buffer; **c**) ^{1}H NMR analysis of the reaction of psEFE with 2OG 12 h post addition of psEFE. Conditions: 400 µM 2OG, 500 µM *L*-arginine, 500 µM *L*-ascorbate, 50 µM Fe(II), 800 µM TMSP-d_4, and 2 µM psEFE in buffer (50 mM sodium phosphate, pH 7.4, 10%$_{v/v}$ D_2O). The unassigned singlets observed in the authentic synthetic sample of dehydroascorbate at ~2.6, ~2.8, and ~2.95 ppm are also present in the reported ^{1}H NMR spectrum of **1d** (8).

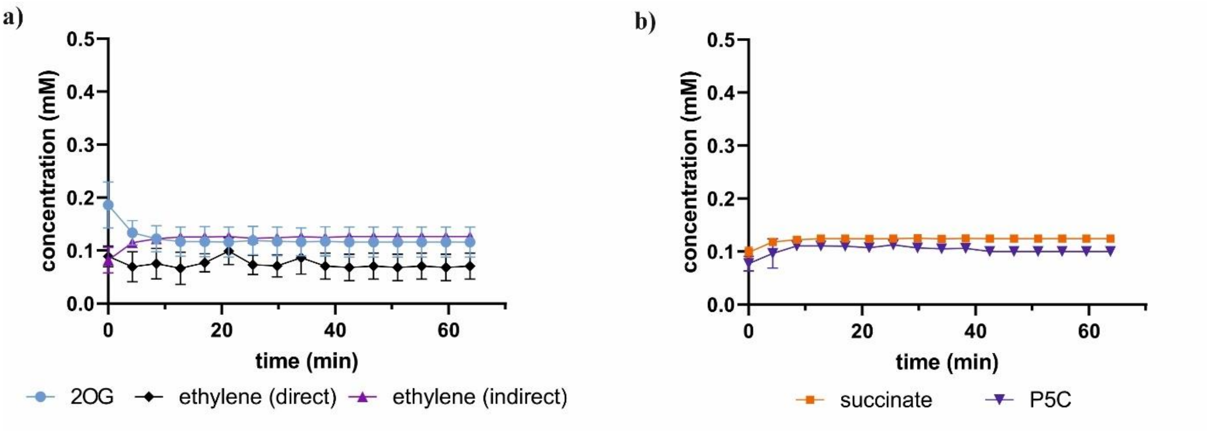

Supporting Figure S5. Quantification of ethylene formation using ^{1}H NMR. a) The indirect quantification of ethylene formation by subtracting the normalized integral of succinate (obtained via comparison with TMSP-*d*$_4$) from that of 2OG (**1**) was more reliable than the direct integration of the ethylene signal (~5.35 ppm), possibly because ethylene evaporates from the reaction mixture; **b**) ^{1}H NMR analysis of the crude reaction mixture implies that psEFE-catalyzed succinate formation correlates stoichiometrically with Arg hydroxylation to give P5C, suggesting that subtracting the normalized integral of succinate (obtained via comparison with TMSP-*d*$_4$) from that of 2OG is a valid method to estimate ethylene formation; note that substantial levels of products other than succinate and ethylene were not detected from 2OG (**1**) during psEFE catalysis (Supporting Figures S1 and S2). Conditions: 400 µM 2OG (**1**), 500 µM *L*-arginine, 500 µM *L*-ascorbate, 50 µM Fe(II), 800 µM TMSP-*d*$_4$, and 2 µM psEFE in buffer (50 mM sodium phosphate, pH 7.4, 10%$_{v/v}$ D_2O).

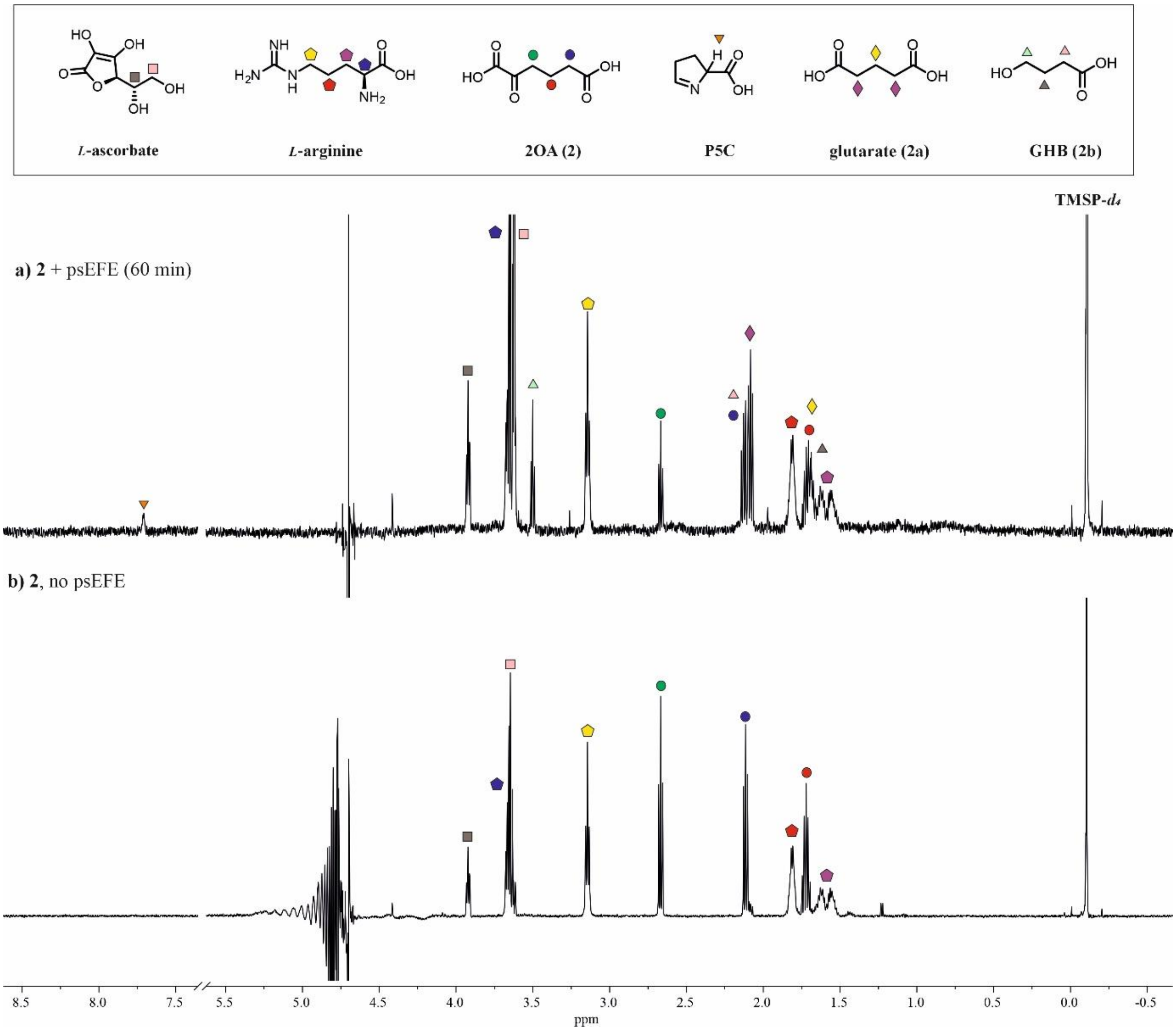

Supporting Figure S6. psEFE catalyses the conversion of *L*-arginine to P5C using 2OA (2) as a cosubstrate. **a**) ^{1}H NMR analysis of the reaction of psEFE with 2OA (**2**) as a cosubstrate reveals formation of P5C; **b**) ^{1}H NMR spectrum of **2** under standard conditions in the absence of psEFE. Conditions: 400 µM **2**, 500 µM *L*-arginine, 500 µM *L*-ascorbate, 50 µM Fe(II), 800 µM TMSP-d_4, and 10 µM psEFE in buffer (50 mM sodium phosphate, pH 7.4, 10%$_{v/v}$ D_2O). Both the doublet observed at ~1.2 ppm and the singlet observed at ~4.4 ppm originate from the enzyme sample; both signals were observed in the enzyme-only control.

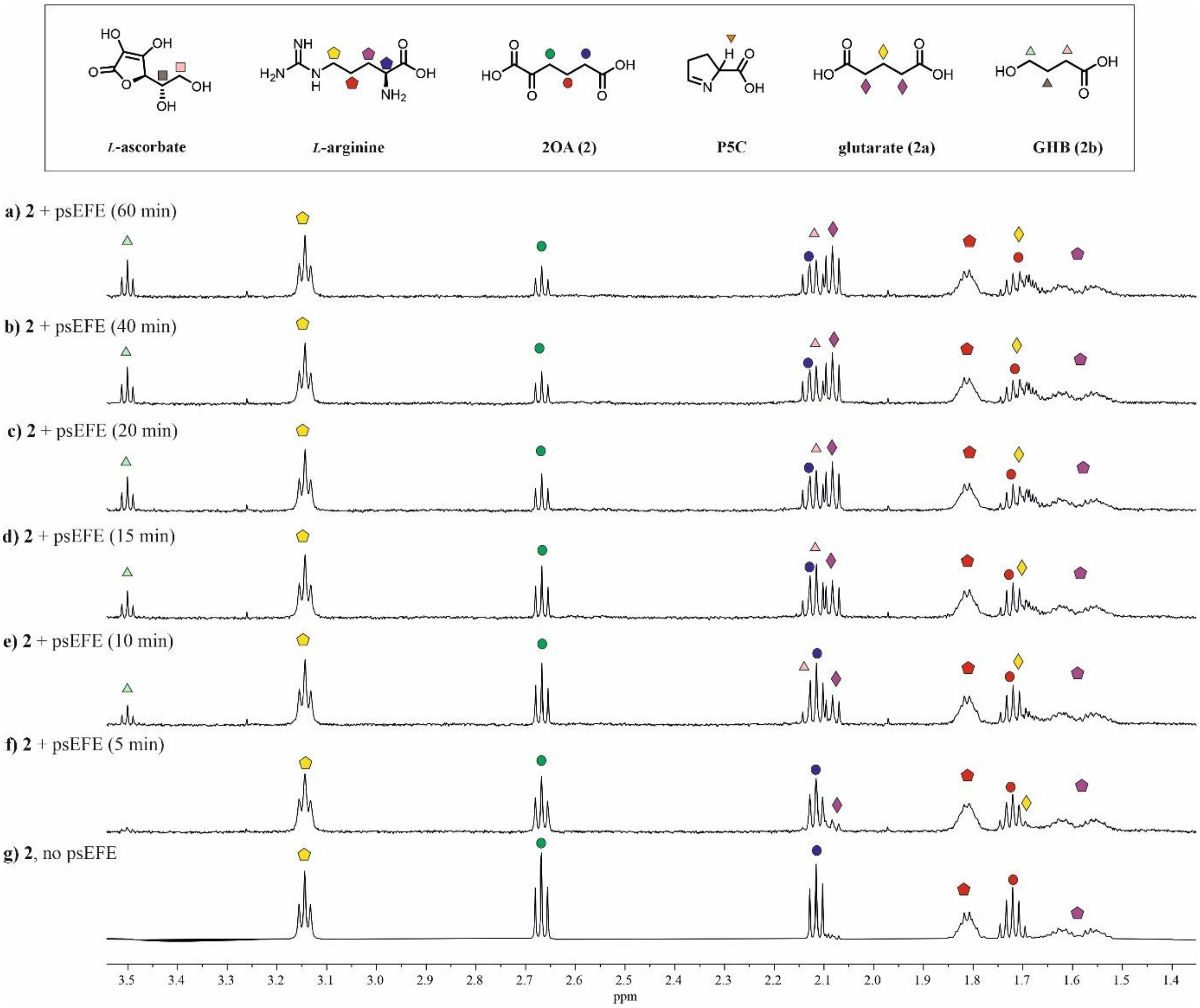

Supporting Figure S7. Time-dependent psEFE-catalyzed conversion of 2OA (2). a-f) Representative ^{1}H NMR spectra (~1.4 to ~3.6 ppm) monitoring the reaction of psEFE with 2OA (**2**) after: **a**) 60 min, **b**) 40 min, **c**) 20 min, **d**) 15 min, **e**) 10 min, and **f**) 5 min. **g**) ^{1}H NMR analysis of the reaction mixture in the absence of psEFE. Conditions: 400 µM **2**, 500 µM *L*-arginine, 500 µM *L*-ascorbate, 50 µM Fe(II), 800 µM TMSP-d_4, and 10 µM psEFE in buffer (50 mM sodium phosphate, pH 7.4, 10%$_{v/v}$ D_2O).

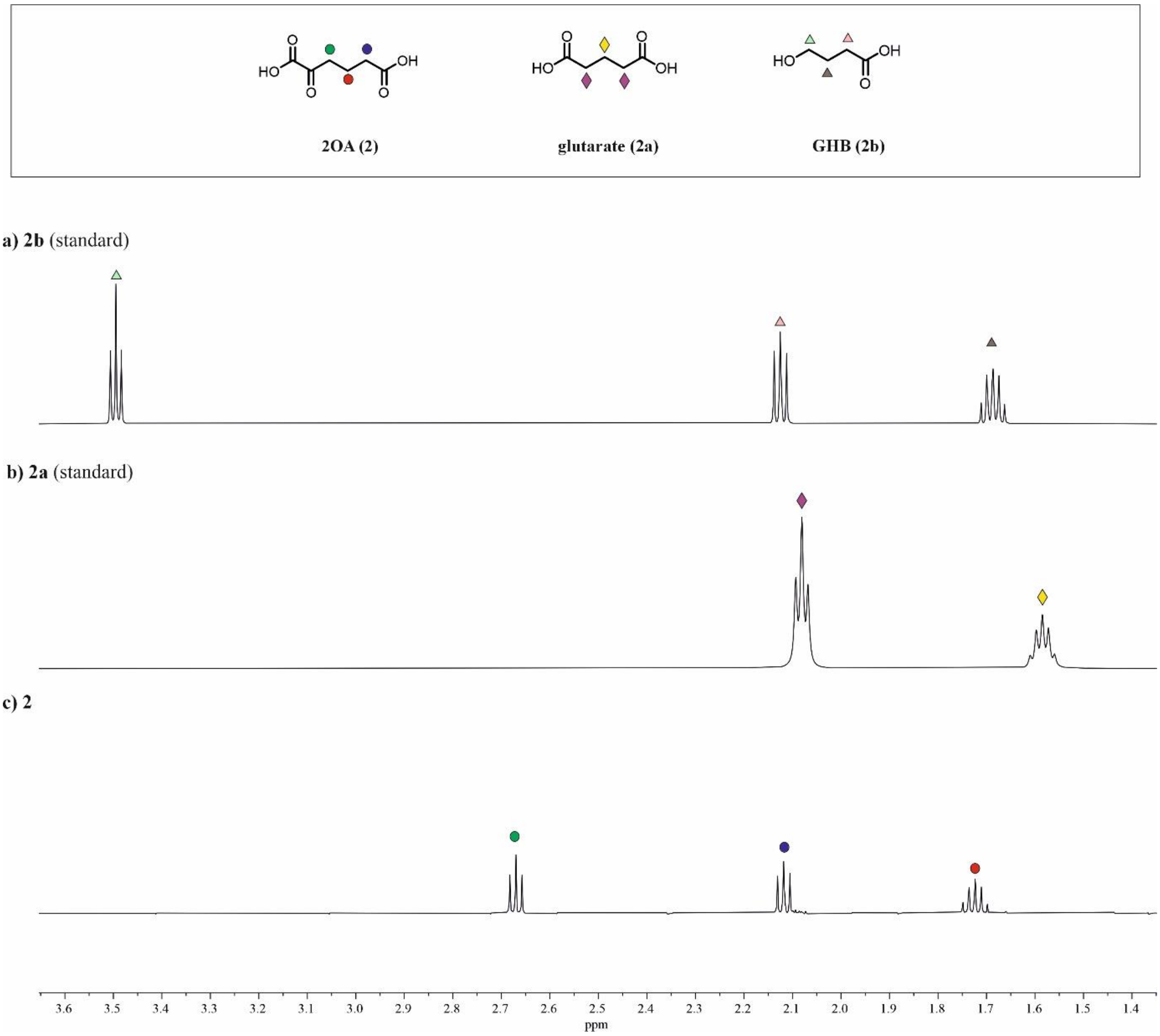

Supporting Figure S8. ^{1}H NMR analysis of 2OA (2) and authentic samples of its products formed during psEFE catalysis. a-c) ^{1}H NMR spectra (~1.4 to ~3.6 ppm) of: **a**) γ-hydroxybutyrate (GHB, **2b**), **b**) glutarate (**2a**), and **c**) 2OA (**2**) in buffer (50 mM sodium phosphate, pH 7.4, 10%$_{v/v}$ D_2O).

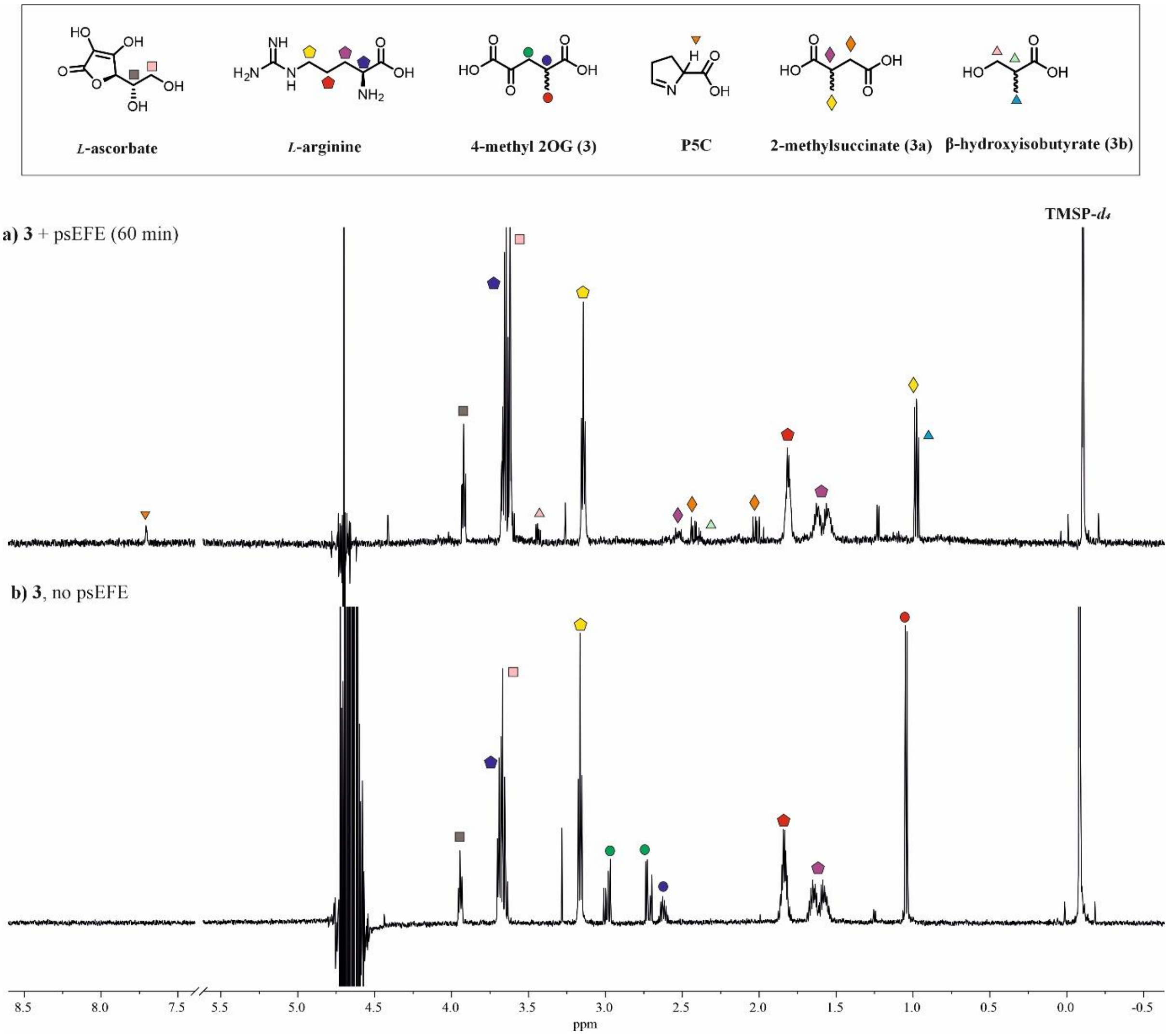

Supporting Figure S9. psEFE catalyzes the conversion of *L*-arginine to P5C using racemic 4-methyl-2OG (3) as a cosubstrate. a) ^{1}H NMR analysis of the reaction of psEFE with 4-methyl-2OG (**3**) reveals formation of P5C; **b**) ^{1}H NMR spectrum of **3** under standard conditions in the absence of psEFE. Conditions: 400 µM **3**, 500 µM *L*-arginine, 500 µM *L*-ascorbate, 50 µM Fe(II), 800 µM TMSP-d_4, and 10 µM psEFE in buffer (50 mM sodium phosphate, pH 7.4, 10%$_{v/v}$ D_2O). Both the doublet observed at ~1.2 ppm and the singlet observed at ~4.4 ppm originate from the enzyme sample; both signals were observed in the enzyme-only control. The stereochemistry of P5C was not assigned, as it may epimerize under the assay conditions.

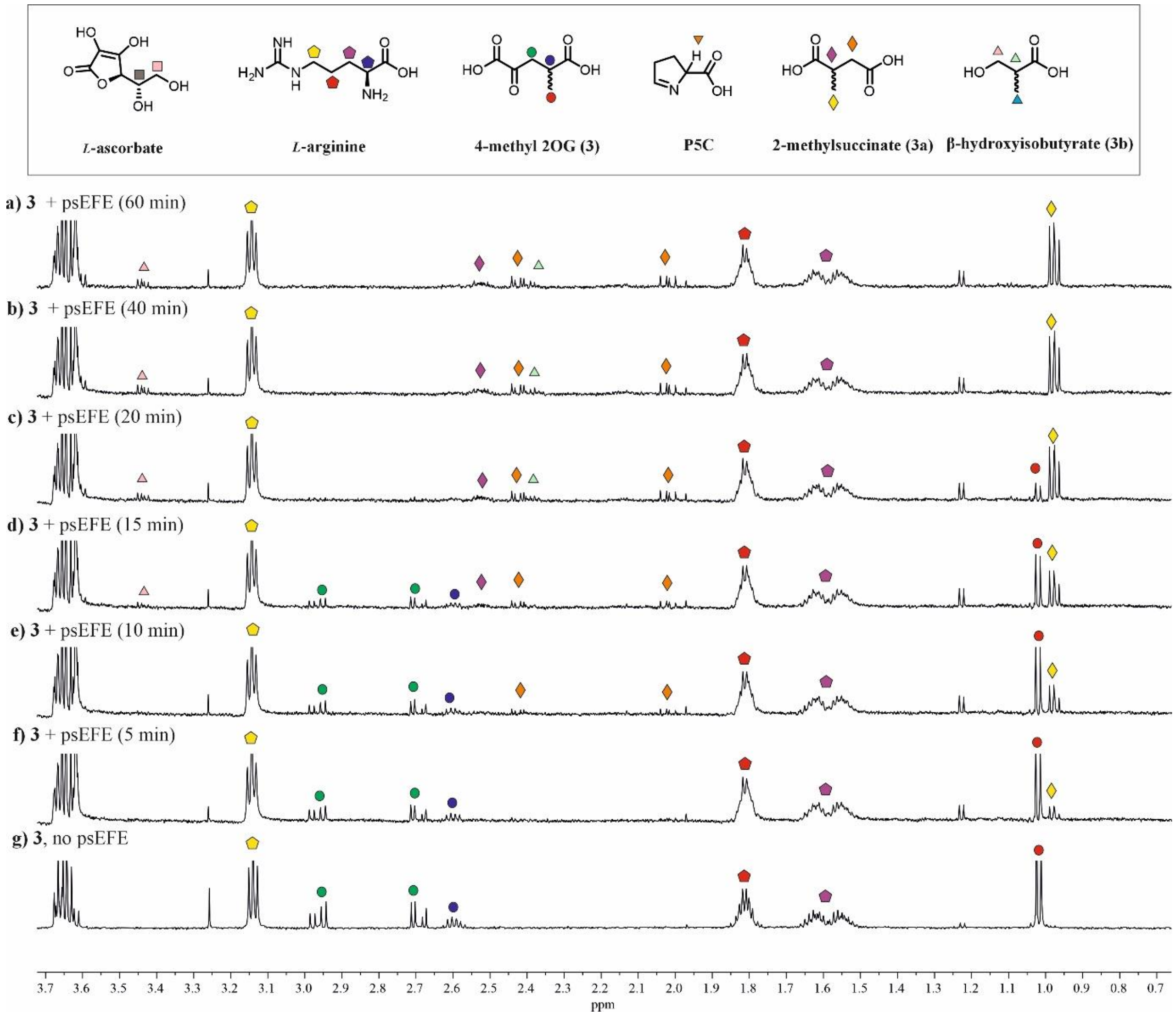

Supporting Figure S10. Time-dependent psEFE-catalyzed conversion of racemic 4-methyl-2OG (3). a-f) Representative ^{1}H NMR spectra (~0.7 to ~3.7 ppm) monitoring the reaction of psEFE with 4-methyl-2OG (**3**) after: **a**) 60 min, **b**) 40 min, **c**) 20 min, **d**) 15 min, **e**) 10 min, and **f**) 5 min. **g**) ^{1}H NMR analysis of the reaction mixture in the absence of psEFE. Conditions: 400 µM **3**, 500 µM *L*-arginine, 500 µM *L*-ascorbate, 50 µM Fe(II), 800 µM TMSP-d_4, and 10 µM psEFE in buffer (50 mM sodium phosphate, pH 7.4, 10%$_{v/v}$ D_2O). The doublet observed at ~1.2 ppm originates from the compound-only control of 4-methyl 2OG (**3**).

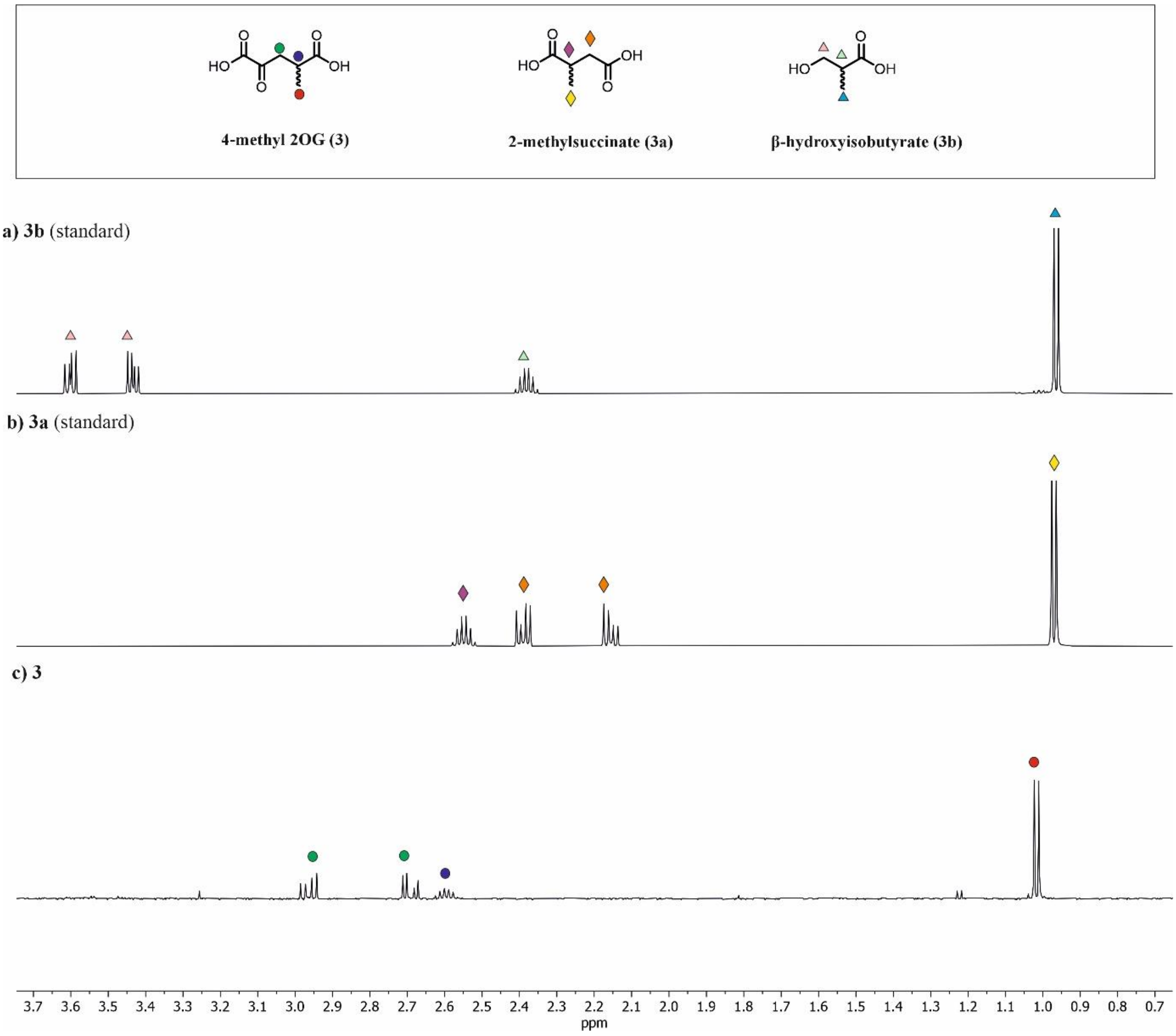

Supporting Figure S11. ^{1}H NMR analysis of racemic 4-methyl-2OG (3) and authentic samples of its products formed during psEFE catalysis. a-c) ^{1}H NMR spectra (~0.7 to ~3.7 ppm) of: **a**) β-hydroxyisobutyrate (**3b**), **b**) 2-methylsuccinate (**3a**), and **c**) 4-methyl 2OG (**3**) in buffer (50 mM sodium phosphate, pH 7.4, 10%$_{v/v}$ D_2O).

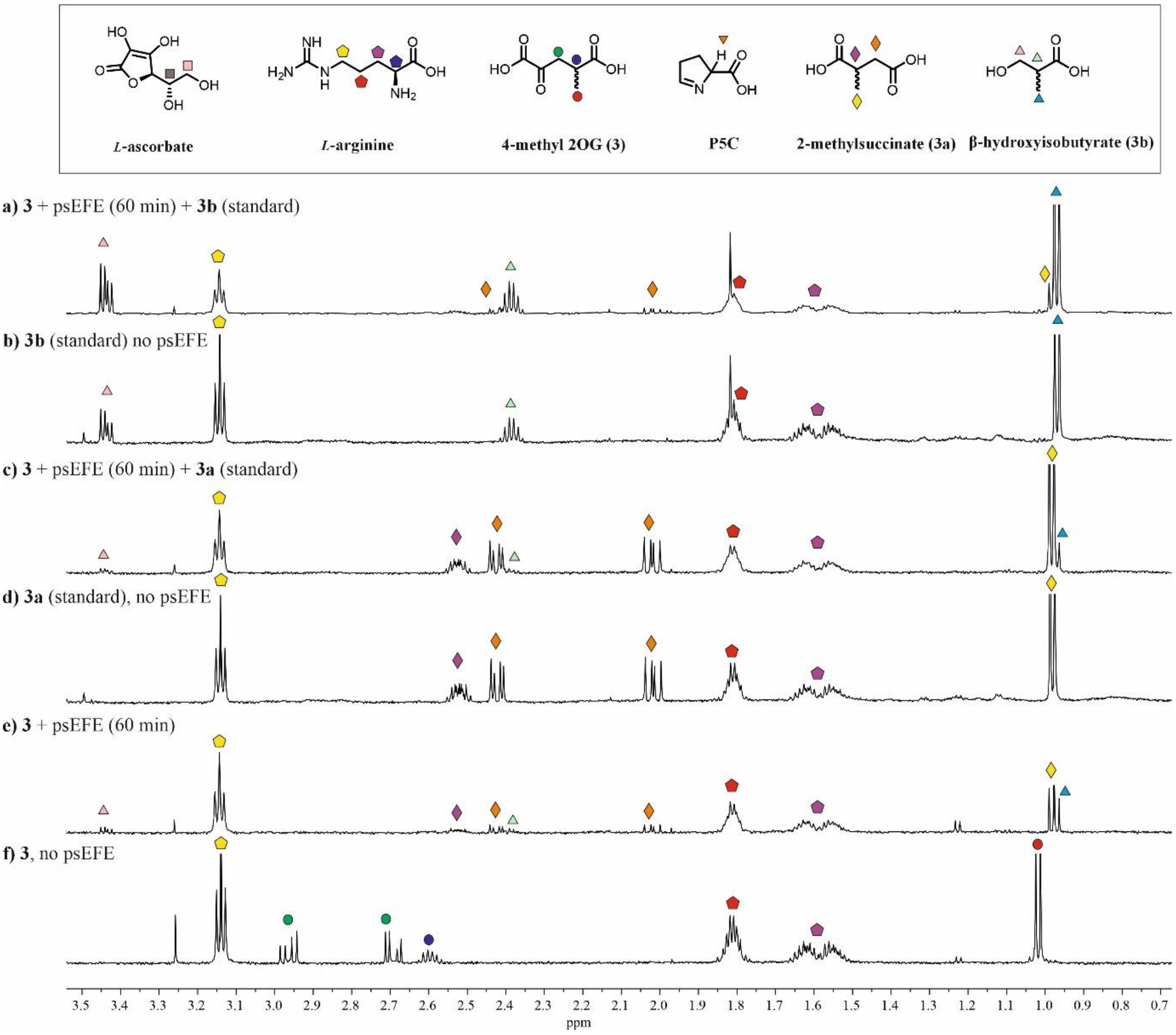

Supporting Figure S12. Analysis of psEFE catalysis using racemic 4-methyl-2OG (3) as a substrate. ^{1}H NMR analysis (~0.7 to ~3.7 ppm) of: **a**) a reaction mixture of psEFE and racemic 4-methyl-2OG (**3**) spiked with β-hydroxyisobutyrate (**3b**), **b**) **3b** in the absence of **3** under standard conditions; **c**) a reaction mixture of psEFE and **3** spiked with 2-methylsuccinate (**3a**), **d**) **3a** in the absence of **3** under standard conditions; **e**) a reaction mixture of psEFE and **3** 80 min post addition of psEFE; **f**) **3** under standard conditions in the absence of psEFE. Conditions: 400 µM **3**, 500 µM L-arginine, 500 µM L-ascorbate, 50 µM Fe(II), 800 µM TMSP-d_4, 10 µM psEFE, and, if appropriate, 2 mM of the appropriate authentic standard in buffer (50 mM phosphate, pH 7.4, 10%$_{v/v}$ D_2O). The singlet observed at ~4.4 ppm originates from the enzyme sample; it was observed in the enzyme-only control.

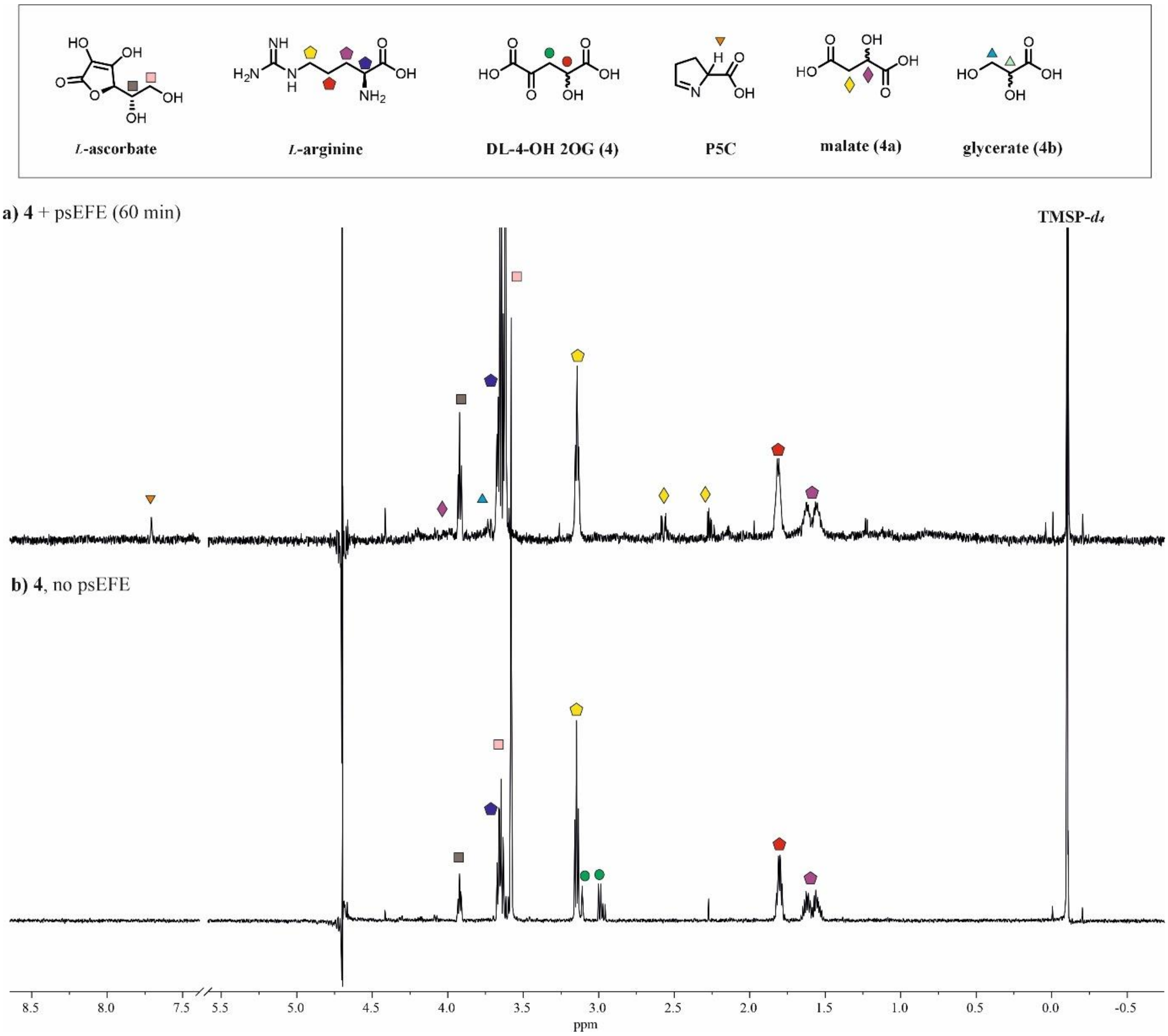

Supporting Figure S13. psEFE catalyzes the conversion of *L*-arginine to P5C using racemic 4-OH-2OG (4) as a cosubstrate. **a**) ^{1}H NMR analysis of the reaction of psEFE with racemic 4-OH-2OG (**4**) reveals formation of P5C; **b**) ^{1}H NMR spectrum of **4** under standard conditions in the absence of psEFE. Conditions: 400 µM **4**, 500 µM *L*-arginine, 500 µM *L*-ascorbate, 50 µM Fe (II), 800 µM TMSP-d_4, and 10 µM psEFE in buffer (50 mM sodium phosphate, pH 7.4, 10%$_{v/v}$ D_2O). Both the doublet observed at ~1.2 ppm and the singlet observed at ~4.4 ppm originate from the enzyme sample; both signals were observed in the enzyme-only control.

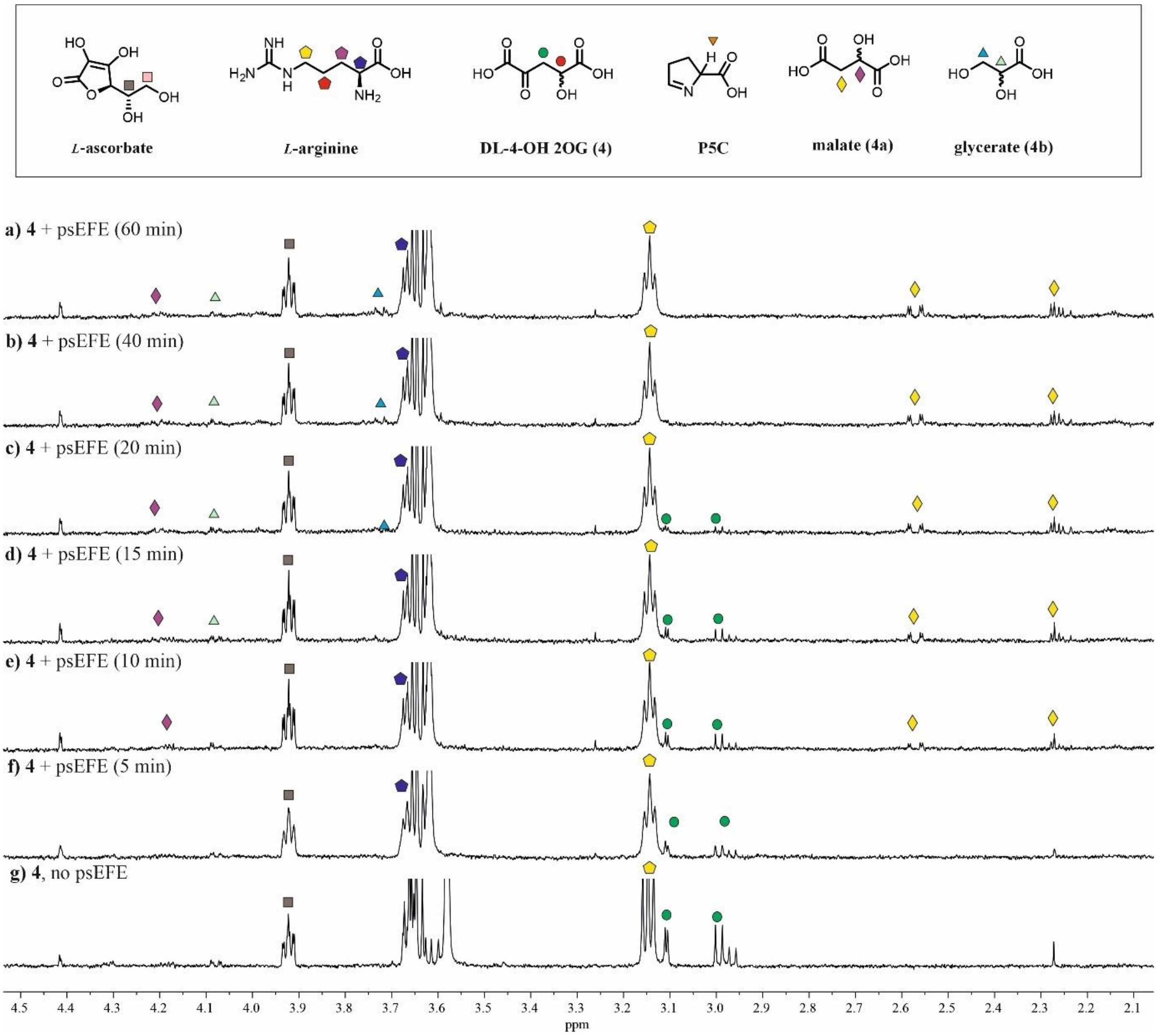

Supporting Figure S14. Time-dependent psEFE-catalyzed conversion of racemic 4-OH-2OG (4). a-f) Representative ^{1}H NMR spectra (~2.1 to ~4.5 ppm) monitoring the reaction of psEFE with racemic 4-OH-2OG (**4**) after: **a**) 60 min, **b**) 40 min, **c**) 20 min, **d**) 15 min, **e**) 10 min, and **f**) 5 min. **g**) ^{1}H NMR analysis of the reaction mixture in the absence of psEFE. Conditions: 400 µM **4**, 500 µM *L*-arginine, 500 µM *L*-ascorbate, 50 µM Fe (II), 800 µM TMSP-d_4, and 10 µM psEFE in buffer (50 mM sodium phosphate, pH 7.4, 10%$_{v/v}$ D_2O). The singlet observed at ~4.4 ppm originates from the enzyme sample; it was observed in the enzyme-only control.

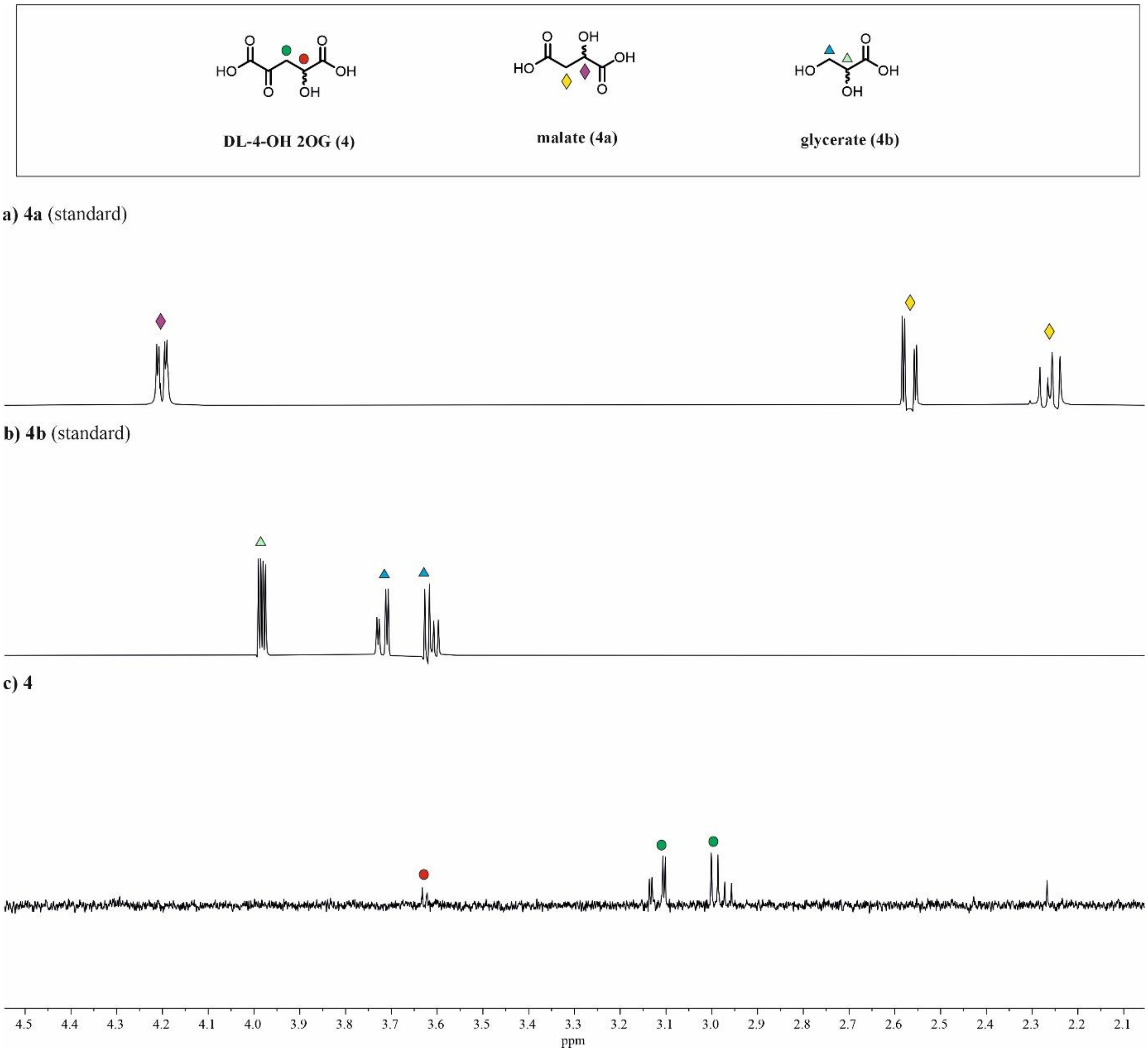

Supporting Figure S15. ^{1}H NMR analysis of racemic 4-OH-2OG (4) and authentic samples of its products formed during psEFE catalysis. a-c) ^{1}H NMR spectra (~2.1 to ~4.5 ppm) of: **a**) malate (**4a**), **b**) glycerate (**4b**), and **c**) racemic 4-OH-2OG (**4**) in buffer (50 mM sodium phosphate, pH 7.4, 10%$_{v/v}$ D_2O).

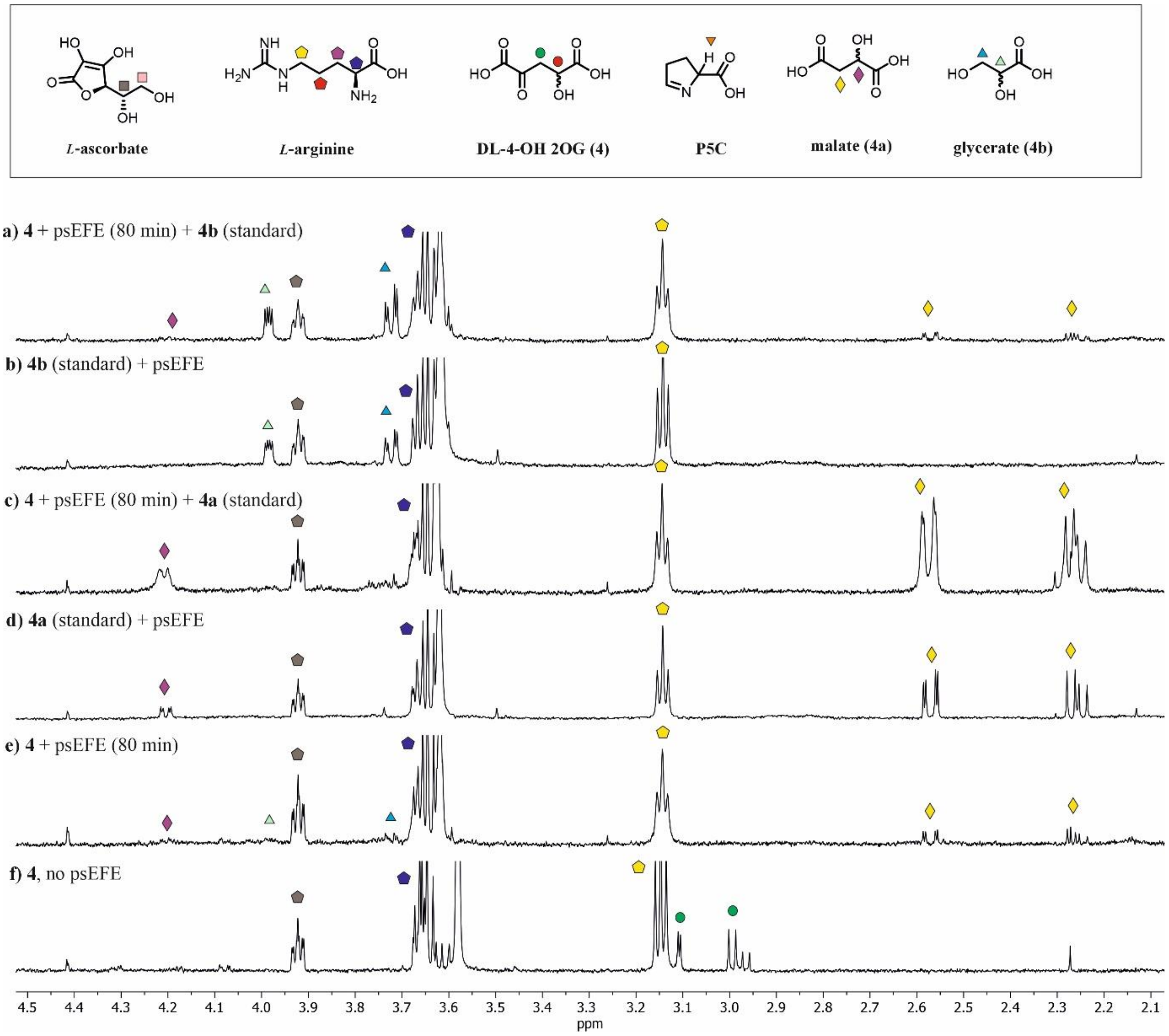

Supporting Figure S16. Analysis of psEFE catalysis using racemic 4-OH-2OG (4) as a substrate. ^{1}H NMR analysis (~2.1 to ~4.5 ppm) of: **a**) a reaction mixture of psEFE and racemic 4-OH-2OG (**4**) spiked with glycerate (**4b**), **b**) **4b** in the absence of **4** under standard conditions; **c**) a reaction mixture of psEFE and **4** spiked with malate (**4a**), **d**) **4a** in the absence of **4** under standard conditions; **e**) a reaction mixture of psEFE and **4** 80 min post addition of psEFE; **f**) **4** under standard conditions in the absence of psEFE. Conditions: 400 µM **4**, 500 µM L-arginine, 500 µM L-ascorbate, 50 µM Fe(II), 800 µM TMSP-d_4, 10 µM psEFE, and, if appropriate, 2 mM of an appropriate authentic standard in buffer (50 mM phosphate, pH 7.4, 10%$_{v/v}$ D_2O). The singlet observed at ~4.4 ppm originates from the enzyme sample; it was observed in the enzyme-only control.

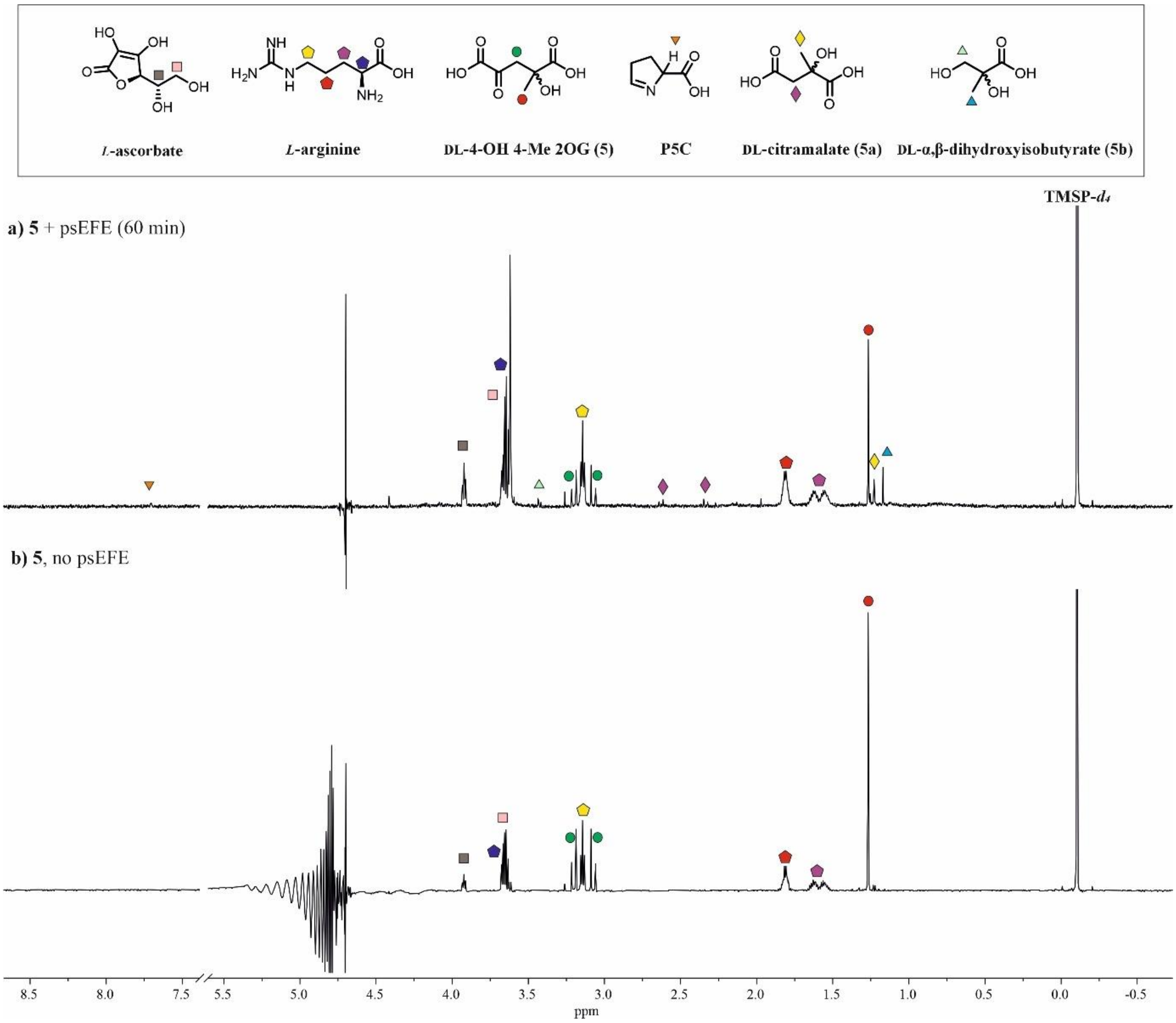

Supporting Figure S17. psEFE catalyzes the conversion of *L*-arginine to P5C using racemic 4-hydroxy-4-methyl-2OG (5) as a cosubstrate. **a**) ^{1}H NMR analysis of the reaction of psEFE with racemic 4-hydroxy-4-methyl-2OG (**5**) as a cosubstrate reveals formation of P5C; **b**) ^{1}H NMR spectrum of **5** under standard conditions in the absence of psEFE. Conditions: 400 µM **5**, 500 µM *L*-arginine, 500 µM *L*-ascorbate, 50 µM Fe (II), 800 µM TMSP-d_4, and 10 µM psEFE in buffer (50 mM sodium phosphate, pH 7.4, 10%$_{v/v}$ D_2O). Both the doublet observed at ~1.2 ppm and the singlet observed at ~4.4 ppm originate from the enzyme sample; both signals were observed in the enzyme-only control.

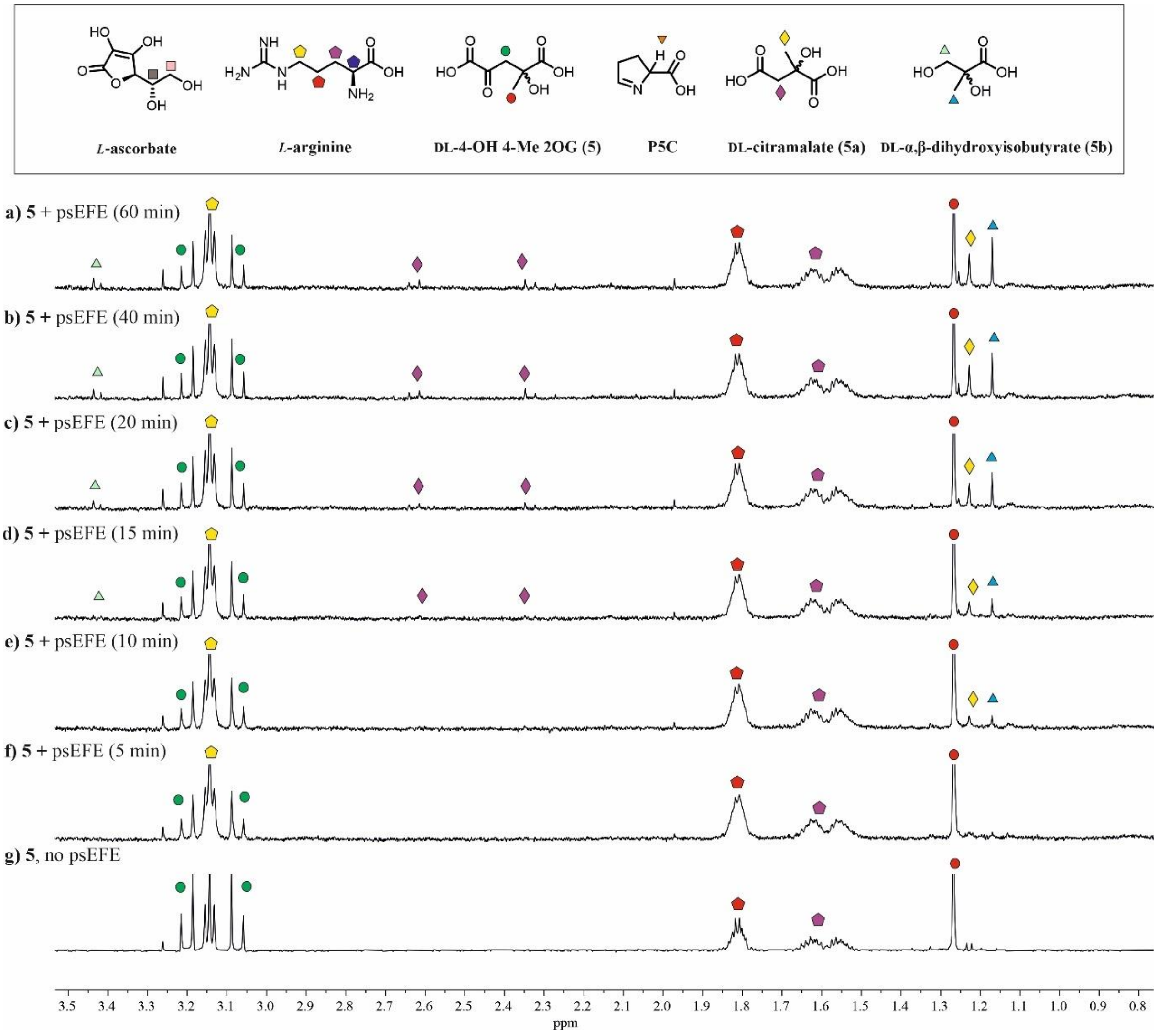

Supporting Figure S18. Time-dependent psEFE-catalyzed conversion of racemic 4-hydroxy-4-methyl-2OG (5). a-f) Representative ^{1}H NMR spectra (~0.8 to ~3.7 ppm) monitoring the reaction of psEFE with racemic 4-hydroxy-4-methyl-2OG (**5**) after: **a**) 60 min, **b**) 40 min, **c**) 20 min, **d**) 15 min, **e**) 10 min, and **f**) 5 min. **g**) ^{1}H NMR analysis of the reaction mixture in the absence of psEFE. Conditions: 400 µM **5**, 500 µM *L*-arginine, 500 µM *L*-ascorbate, 50 µM Fe (II), 800 µM TMSP-d_4, and 10 µM psEFE in buffer (50 mM sodium phosphate, pH 7.4, 10%$_{v/v}$ D_2O). The doublet observed at ~1.2 ppm originates from the enzyme sample; it was observed in the enzyme-only control.

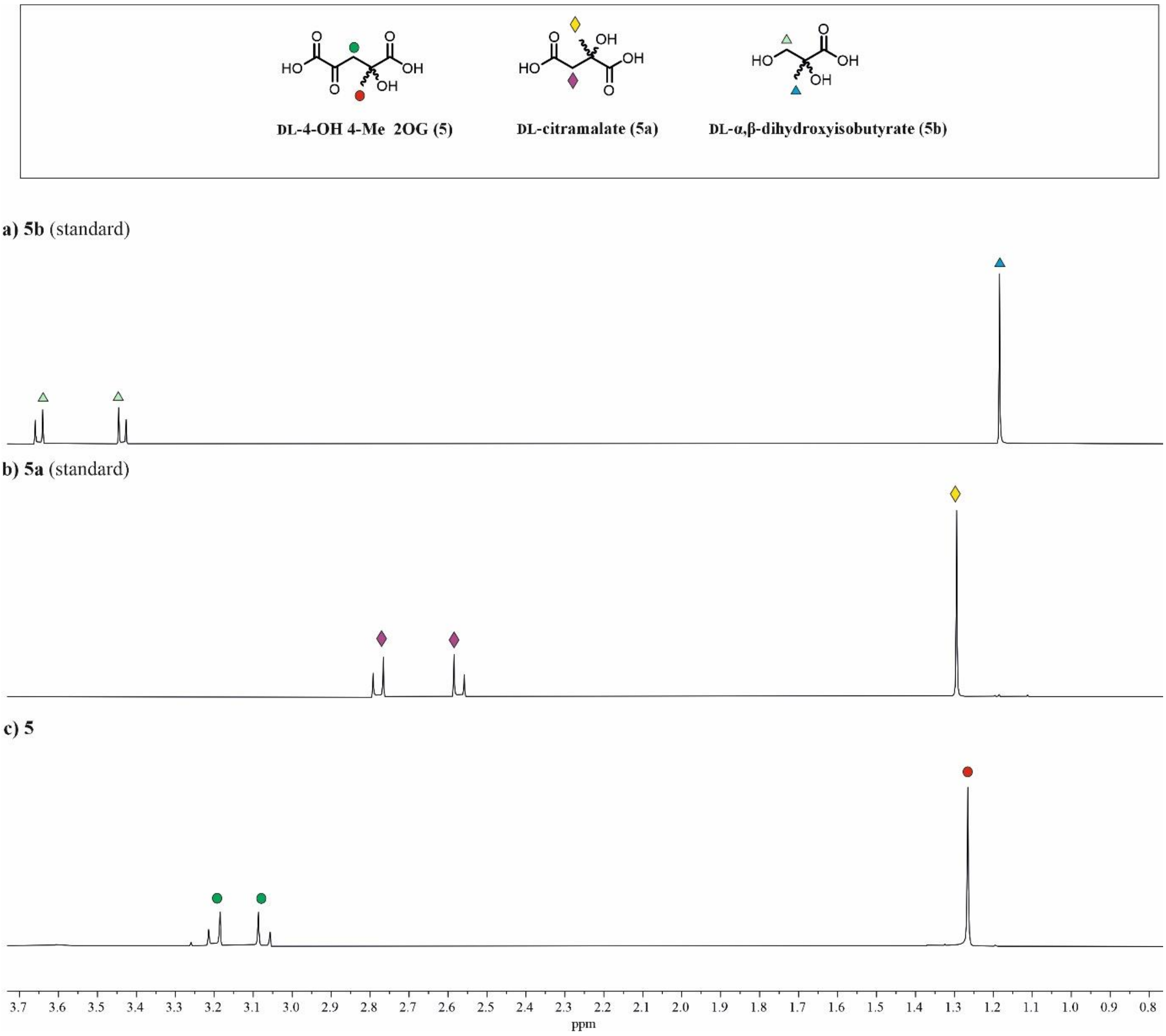

Supporting Figure S19. ^{1}H NMR analysis of racemic 4-hydroxy-4-methyl-2OG (5) and authentic samples of its products formed during psEFE catalysis. a-c) ^{1}H NMR spectra (~0.8 to ~3.7 ppm) of: **a**) racemic α,β-dihydroxyisobutyrate (**5b**), **b**) racemic citramalate (**5a**), and **c**) racemic 4-hydroxy-4-methyl-2OG (**5**) in buffer (50 mM sodium phosphate, pH 7.4, 10%$_{v/v}$ D_2O).

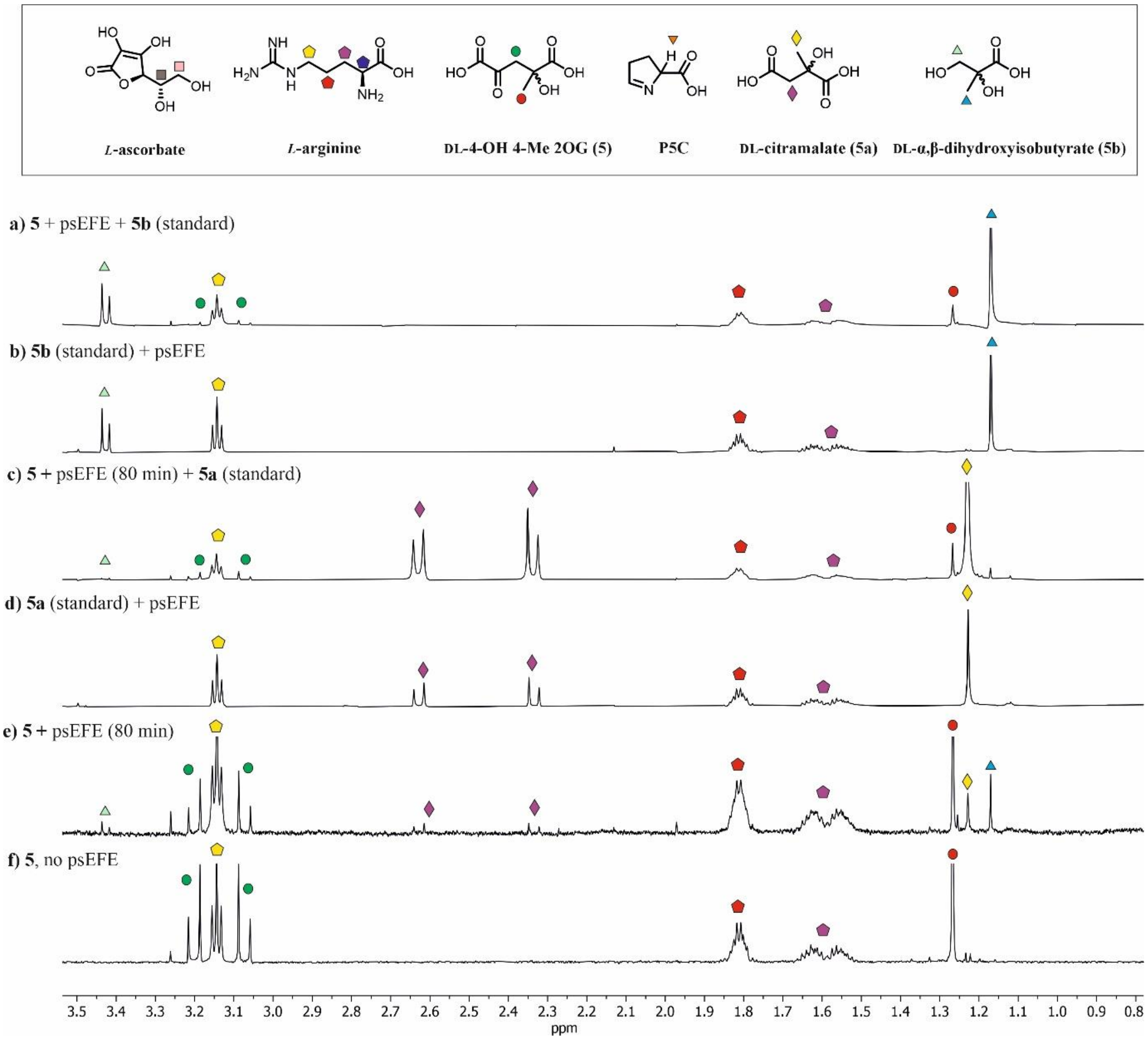

Supporting Figure S20. Analysis of psEFE catalysis using racemic 4-hydroxy-4-methyl-2OG (5) as a substrate. ^{1}H NMR analysis (~0.8 to ~3.7 ppm) of: **a**) a reaction mixture of psEFE and racemic 4-hydroxy-4-methyl-2OG (**5**) spiked with racemic α,β-dihydroxyisobutyrate (**5b**), **b**) **5b** in the absence of **5** under standard conditions; **c**) a reaction mixture of psEFE and **5** spiked with racemic citramalate (**5a**), **d**) **5a** in the absence of **5** under standard conditions; **e**) a reaction mixture of psEFE and **5** 80 min post addition of psEFE; **f**) **5** under standard conditions in the absence of psEFE. Conditions: 400 µM **5**, 500 µM L-arginine, 500 µM L-ascorbate, 50 µM Fe (II), 800 µM TMSP-d_4, 10 µM psEFE, and, if appropriate, 2 mM of an appropriate authentic standard in buffer (50 mM phosphate, pH 7.4, 10%$_{v/v}$ D_2O). The doublet observed at ~1.2 ppm originates from the enzyme sample; it was observed in the enzyme-only control.

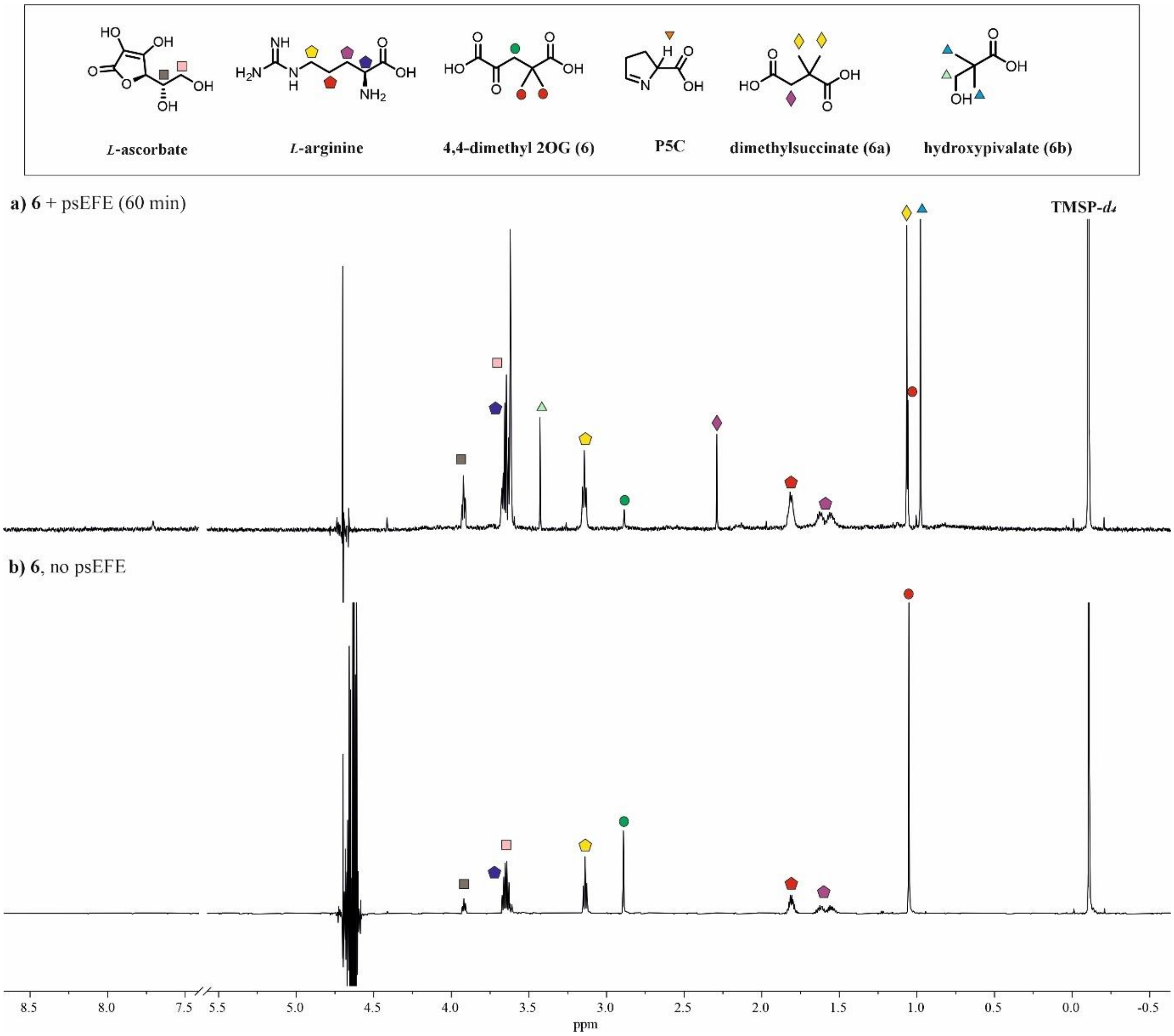

Supporting Figure S21. psEFE catalyzes the conversion of *L*-arginine to P5C using 4,4-dimethyl-2OG (6) as a cosubstrate. a) ^{1}H NMR analysis of the reaction of psEFE with 4,4-dimethyl-2OG (**6**) as a cosubstrate reveals formation of P5C; **b**) ^{1}H NMR spectrum of **5** under standard conditions in the absence of psEFE. Conditions: 400 µM **6**, 500 µM *L*-arginine, 500 µM *L*-ascorbate, 50 µM Fe (II), 800 µM TMSP-d_4, and 10 µM psEFE in buffer (50 mM sodium phosphate, pH 7.4, 10%$_{v/v}$ D_2O).

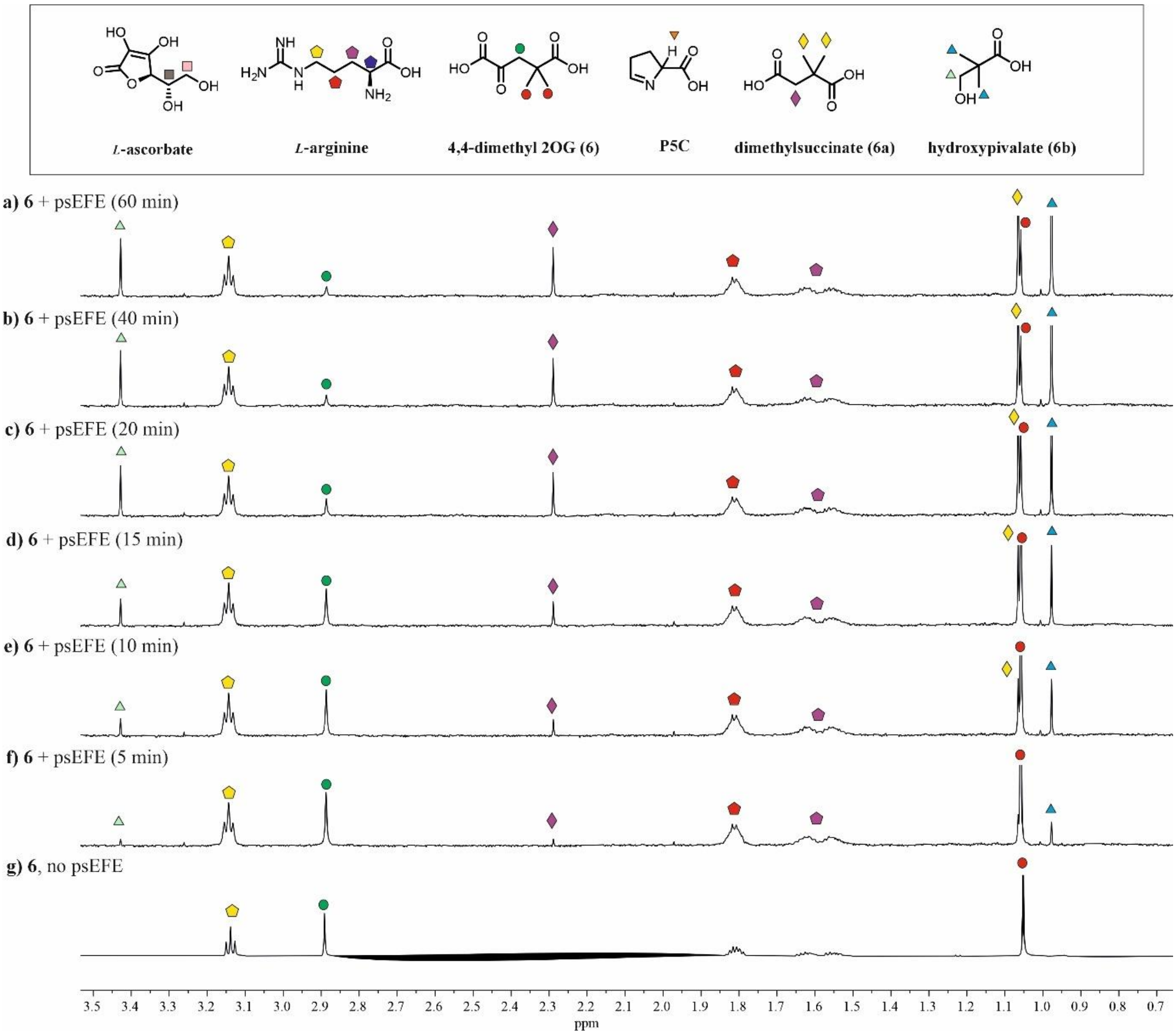

Supporting Figure S22. Time-dependent psEFE-catalyzed conversion of 4,4-dimethyl-2OG (6). a-f) Representative ^{1}H NMR spectra (~0.7 to ~3.5 ppm) monitoring the reaction of psEFE with 4,4-dimethyl-2OG (**6**) after: **a**) 60 min, **b**) 40 min, **c**) 20 min, **d**) 15 min, **e**) 10 min, and **f**) 5 min. **g**) ^{1}H NMR analysis of the reaction mixture in the absence of psEFE. Conditions: 400 µM **6**, 500 µM *L*-arginine, 500 µM *L*-ascorbate, 50 µM Fe (II), 800 µM TMSP-d_4, and 10 µM psEFE in buffer (50 mM sodium phosphate, pH 7.4, 10%$_{v/v}$ D_2O).

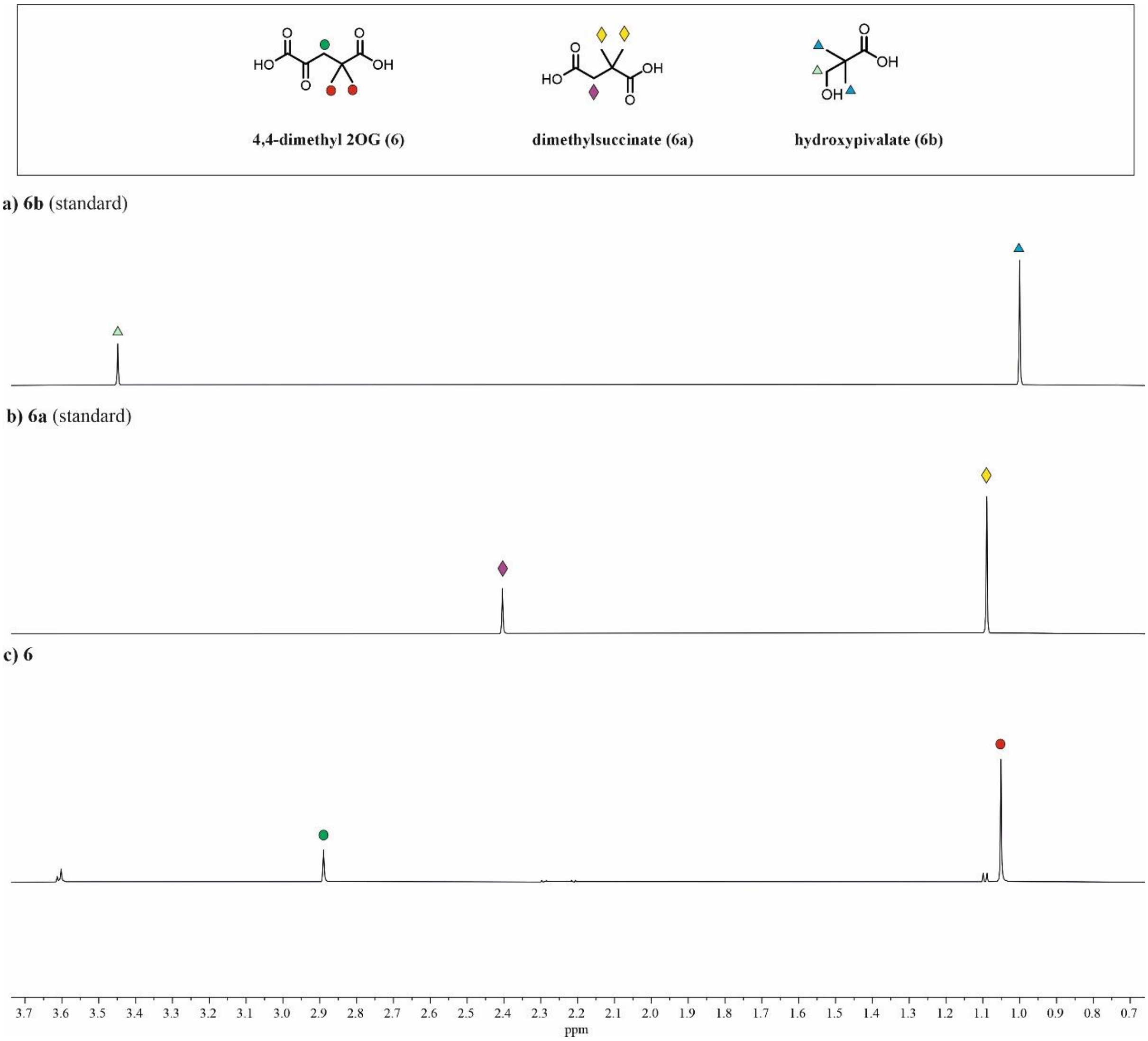

Supporting Figure S23. ^{1}H NMR analysis of 4,4-dimethyl-2OG (6) and authentic samples of its products formed during psEFE catalysis. a-c) ^{1}H NMR spectra (~0.7 to ~3.7 ppm) of: **a**) hydroxypivalate (**6b**), **b**) 2,2-dimethylsuccinate (**6a**), and **c**) 4,4-dimethyl-2OG (**6**) in buffer (50 mM sodium phosphate, pH 7.4, 10%$_{v/v}$ D_2O).

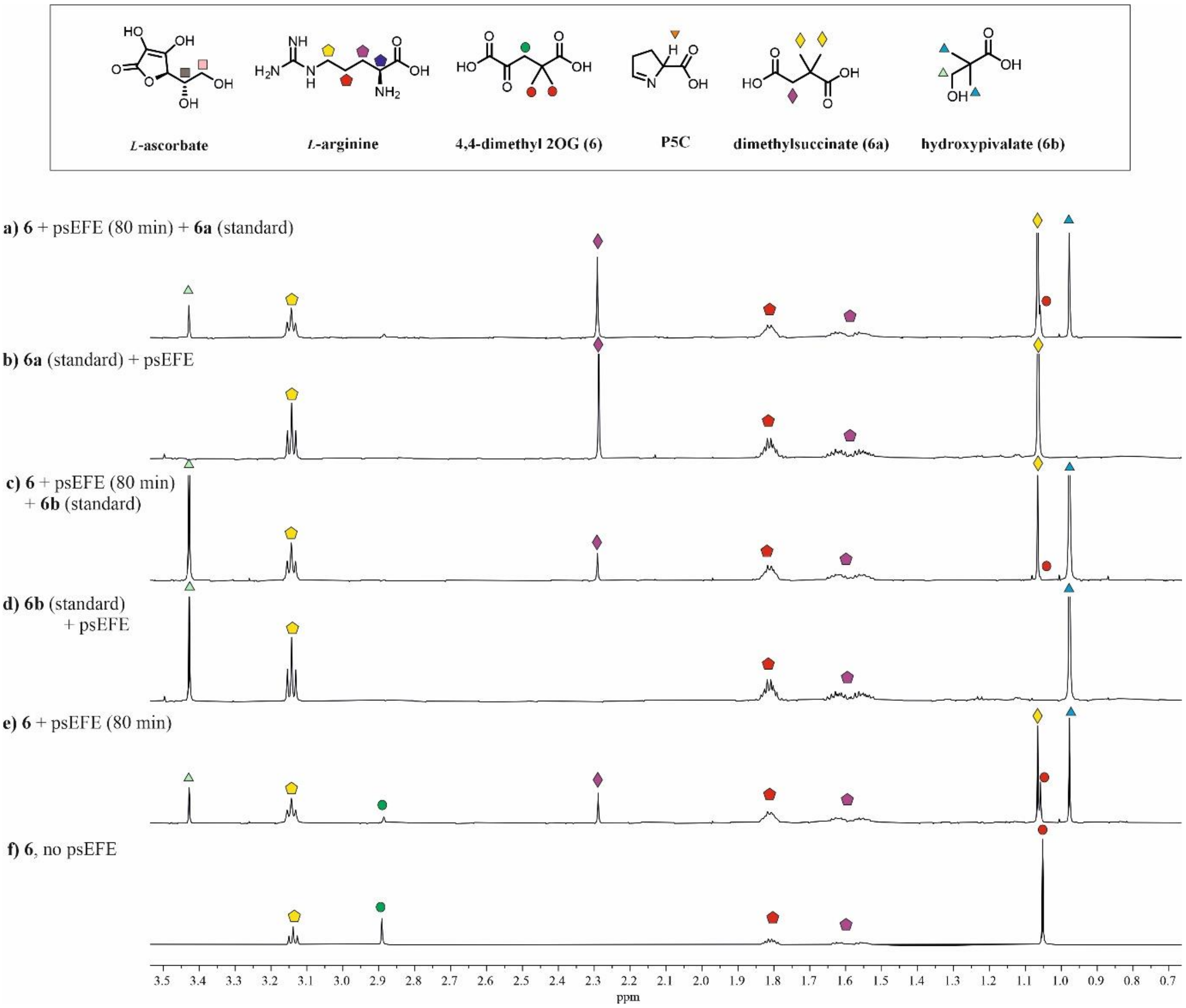

Supporting Figure S24. Analysis of psEFE catalysis using 4,4-dimethyl-2OG (6) as a substrate. ^{1}H NMR analysis (~0.7 to ~3.7 ppm) of: **a**) a reaction mixture of psEFE and 4,4-dimethyl-2OG (**6**) spiked with 2,2-dimethylsuccinate (**6a**), **b**) **6a** in the absence of **6** under standard conditions; **c**) a reaction mixture of psEFE and **6** spiked with hydroxypivalate (**6b**), **d**) **6b** in the absence of **6** under standard conditions; **e**) a reaction mixture of psEFE and **6** 80 min post addition of psEFE; **f**) **6** under standard conditions in the absence of psEFE. Conditions: 400 µM **6**, 500 µM L-arginine, 500 µM L-ascorbate, 50 µM Fe(II), 800 µM TMSP-d_4, 10 µM psEFE, and, if appropriate, 2 mM of an appropriate authentic standard in buffer (50 mM phosphate, pH 7.4, $10\%_{v/v}$ D_2O).

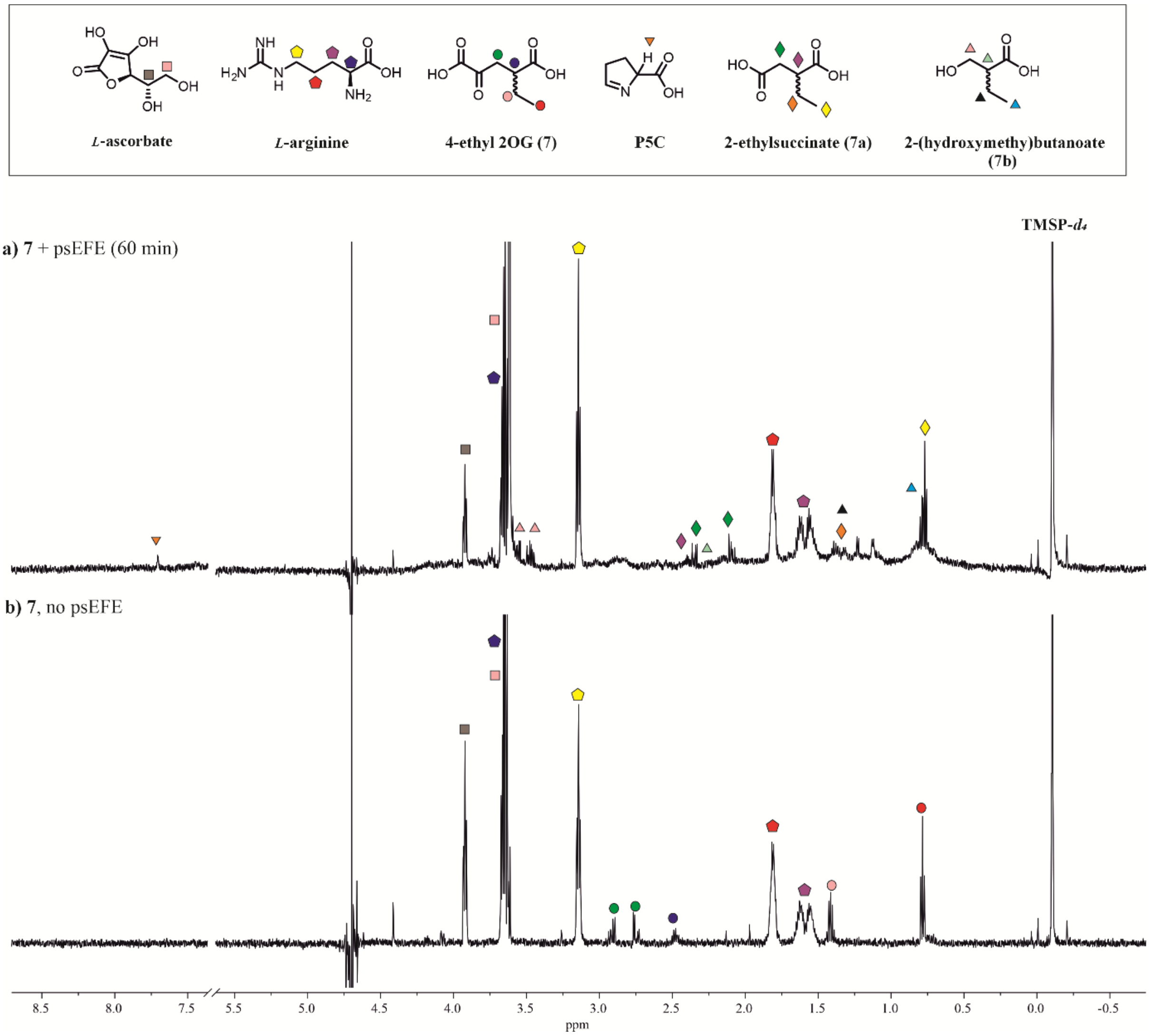

Supporting Figure S25. psEFE catalyzes the conversion of *L*-arginine to P5C using racemic 4-ethyl-2OG (7) as a cosubstrate. a) ^{1}H NMR analysis of the reaction of psEFE with racemic 4-ethyl-2OG (**7**) as a cosubstrate reveals formation of P5C; **b**) ^{1}H NMR spectrum of **7** under standard conditions in the absence of psEFE. Conditions: 400 µM **7**, 500 µM *L*-arginine, 500 µM *L*-ascorbate, 50 µM Fe (II), 800 µM TMSP-d_4, and 10 µM psEFE in buffer (50 mM sodium phosphate, pH 7.4, $10\%_{v/v}$ D_2O). Both the doublet observed at ~1.2 ppm and the singlet observed at ~4.4 ppm originate from the enzyme sample; both signals were observed in the enzyme-only control.

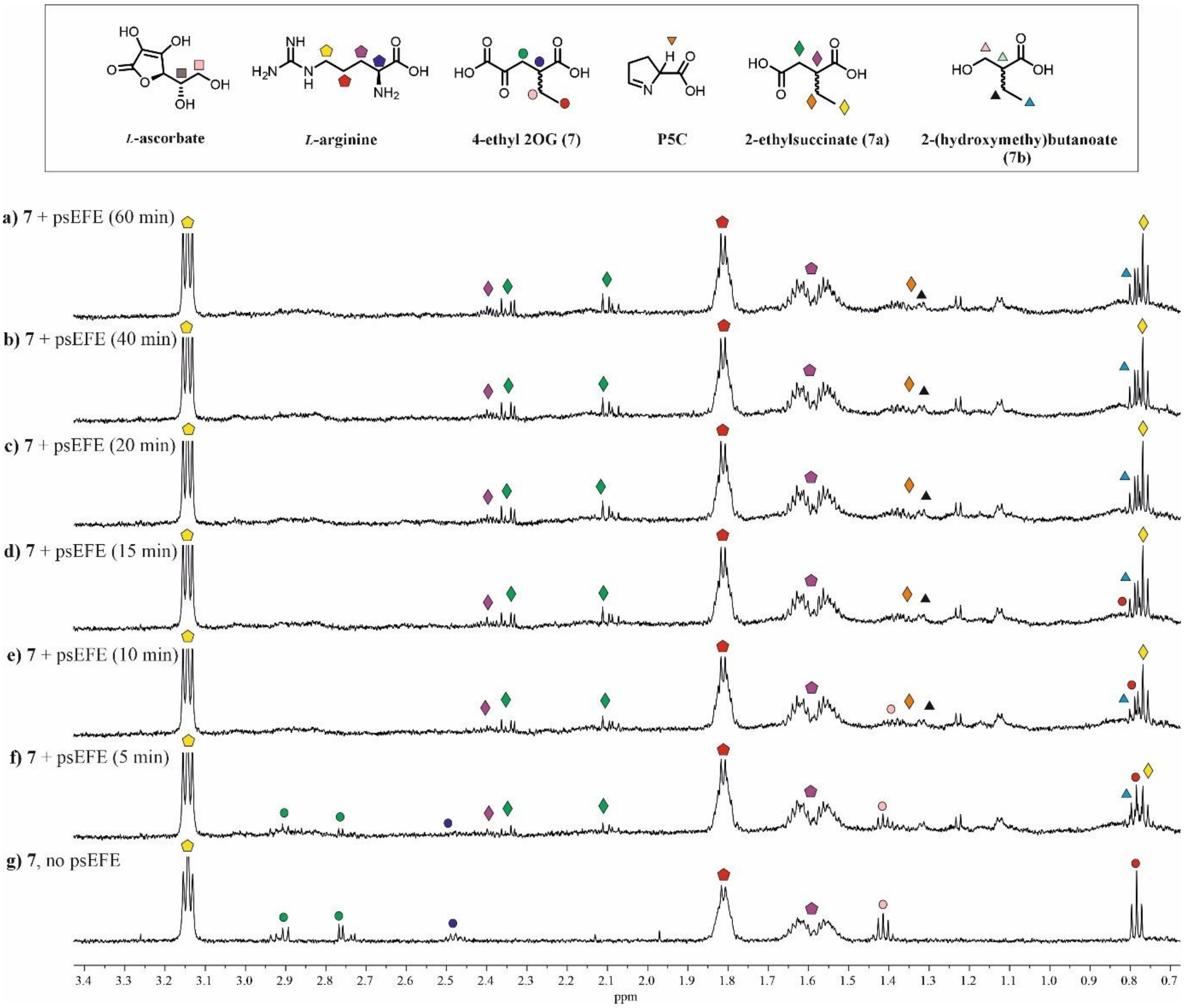

Supporting Figure S26. Time-dependent psEFE-catalyzed conversion of racemic 4-ethyl-2OG (7). a-f) Representative ^{1}H NMR spectra (~0.7 to ~3.4 ppm) monitoring the reaction of psEFE with racemic 4-ethyl-2OG (**6**) after: **a**) 60 min, **b**) 40 min, **c**) 20 min, **d**) 15 min, **e**) 10 min, and **f**) 5 min. **g**) ^{1}H NMR analysis of the reaction mixture in the absence of psEFE. Conditions: 400 µM **7**, 500 µM *L*-arginine, 500 µM *L*-ascorbate, 50 µM Fe (II), 800 µM TMSP-d_4, and 10 µM psEFE in buffer (50 mM sodium phosphate, pH 7.4, 10%$_{v/v}$ D_2O). The doublet observed at ~1.2 ppm originates from the enzyme sample; it was observed in the enzyme-only control.

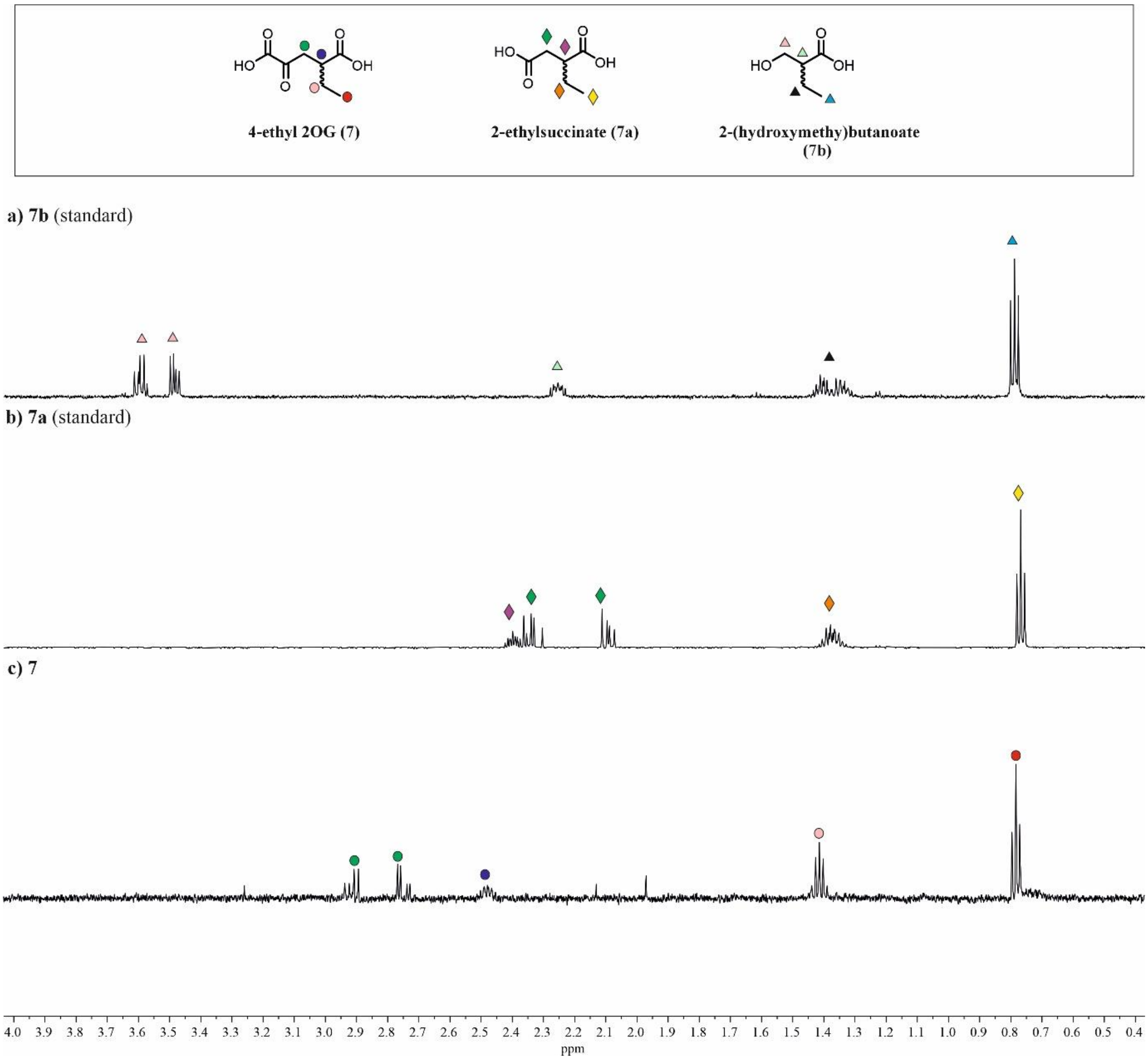

Supporting Figure S27. ^{1}H NMR analysis of racemic 4-ethyl-2OG (7) and authentic samples of its products formed during psEFE catalysis. a-c) ^{1}H NMR spectra (~0.4 to ~4.0 ppm) of: **a**) racemic 2-(hydroxymethyl)butyrate (**7b**), **b**) racemic 2-ethylsuccinate (**7a**), and **c**) racemic 4-ethyl-2OG (**7**) in buffer (50 mM sodium phosphate, pH 7.4, 10%$_{v/v}$ D_2O).

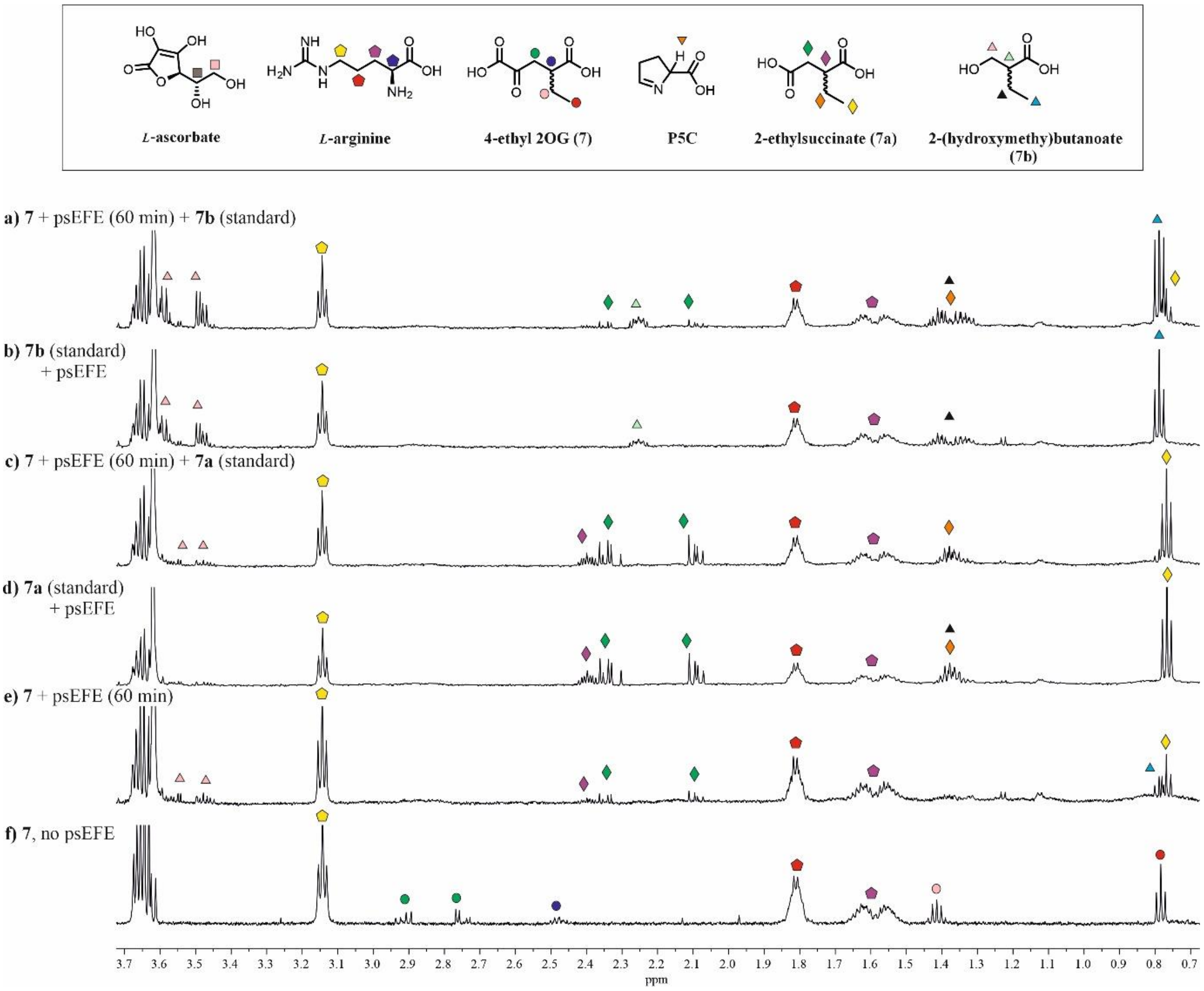

Supporting Figure S28. Analysis of psEFE catalysis using racemic 4-ethyl-2OG (7) as a substrate. ^{1}H NMR analysis (~0.7 to ~3.7 ppm) of: **a**) a reaction mixture of psEFE and racemic 4-ethyl-2OG (**7**) spiked with 2-(hydroxymethyl)butyrate (**7b**), **b**) **7b** in the absence of **7** under standard conditions; **c**) a reaction mixture of psEFE and **7** spiked with 2-ethylsuccinate (**7a**), **d**) **7a** in the absence of **7** under standard conditions; **e**) a reaction mixture of psEFE and **7** 160 min post addition of psEFE; **f**) **7** under standard conditions in the absence of psEFE. Conditions: 400 µM **7**, 500 µM L-arginine, 500 µM L-ascorbate, 50 µM Fe(II), 800 µM TMSP-d_4, 10 µM psEFE, and, if appropriate, 2 mM of an appropriate authentic standard in buffer (50 mM phosphate, pH 7.4, 10%$_{v/v}$ D_2O). The doublet observed at ~1.2 ppm originates from the enzyme sample; it was observed in the enzyme-only control.

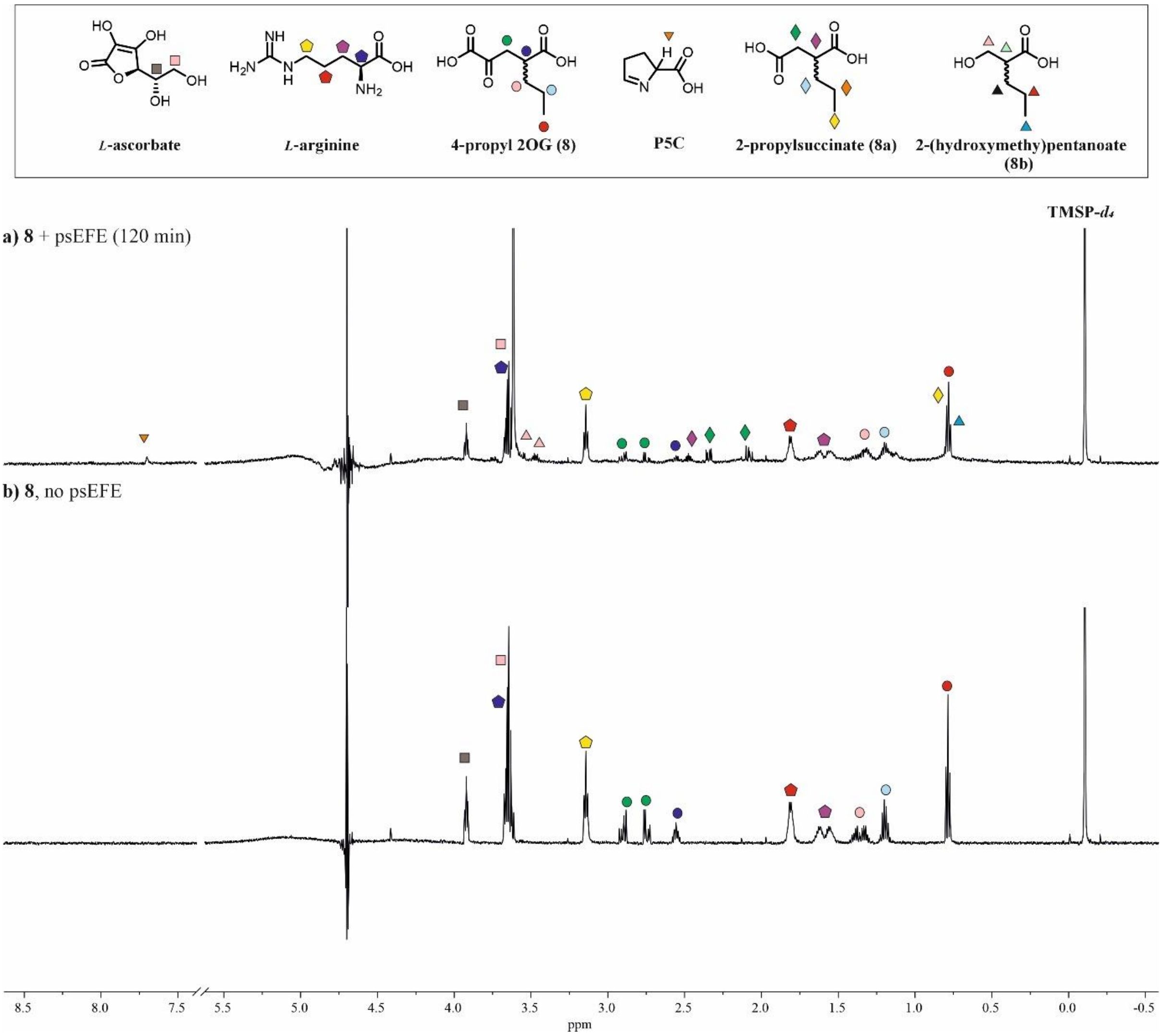

Supporting Figure S29. psEFE catalyses the conversion of *L*-arginine to P5C using racemic 4-propyl-2OG (8) as a cosubstrate. a) ^{1}H NMR analysis of the reaction of psEFE with racemic 4-propyl-2OG (**8**) as a cosubstrate reveals formation of P5C; **b**) ^{1}H NMR spectrum of **8** under standard conditions in the absence of psEFE. Conditions: 400 µM **8**, 500 µM *L*-arginine, 500 µM *L*-ascorbate, 50 µM Fe (II), 800 µM TMSP-d_4, and 10 µM psEFE in buffer (50 mM sodium phosphate, pH 7.4, 10%$_{v/v}$ D_2O). Both the doublet observed at ~1.2 ppm and the singlet observed at ~4.4 ppm originate from the enzyme sample; both signals were observed in the enzyme-only control.

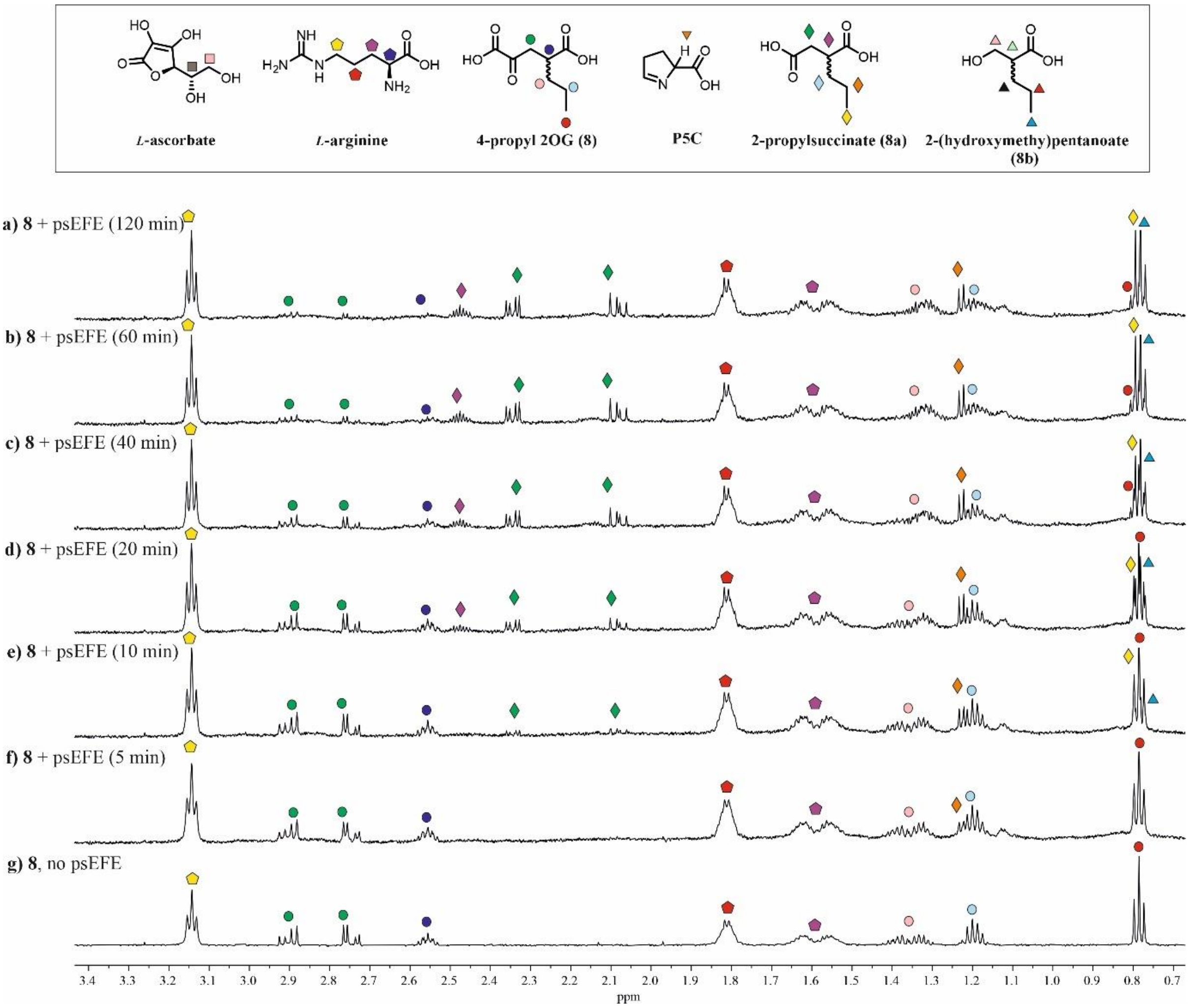

Supporting Figure S30. Time-dependent psEFE-catalyzed conversion of racemic 4-propyl-2OG (8). a-f) Representative ^{1}H NMR spectra (~0.7 to ~3.7 ppm) monitoring the reaction of psEFE with racemic 4-propyl-2OG (**8**) after: **a**) 120 min, **b**) 60 min, **c**) 40 min, **d**) 20 min, **e**) 10 min, and **f**) 5 min. **g**) ^{1}H NMR analysis of the reaction mixture in the absence of psEFE. Conditions: 400 µM **8**, 500 µM *L*-arginine, 500 µM *L*-ascorbate, 50 µM Fe (II), 800 µM TMSP-d_4, and 10 µM psEFE in buffer (50 mM sodium phosphate, pH 7.4, 10%$_{v/v}$ D_2O).

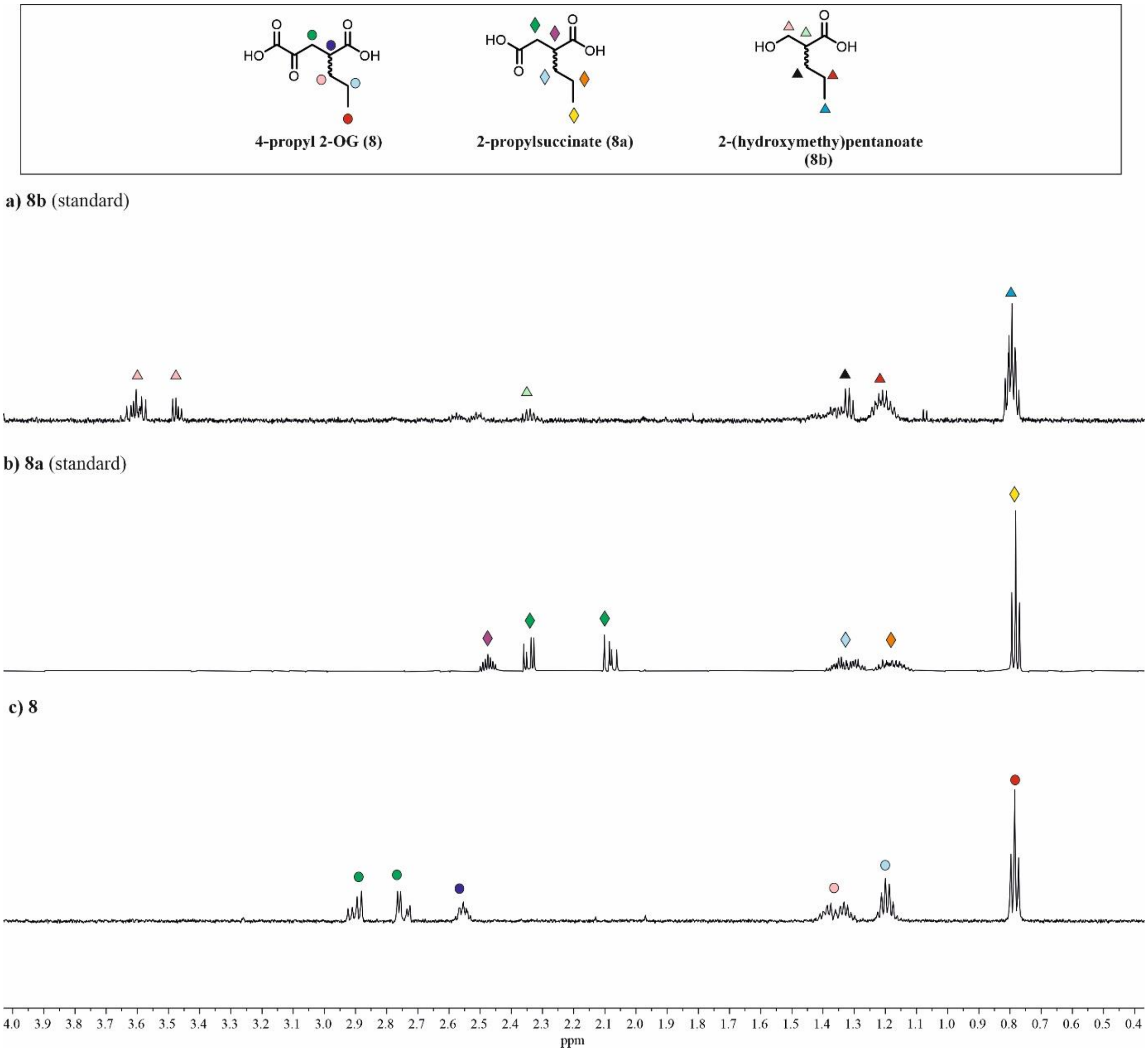

Supporting Figure S31. ^{1}H NMR analysis of racemic 4-propyl-2OG (8) and authentic samples of its products formed during psEFE catalysis. a-c) ^{1}H NMR spectra (~0.4 to ~4.0 ppm) of: **a**) 2-(hydroxymethyl)pentanoate (**8b**), **b**) 2-propylsuccinate (**8a**), and **c**) racemic 4-propyl-2OG (**8**) in buffer (50 mM sodium phosphate, pH 7.4, 10%$_{v/v}$ D_2O).

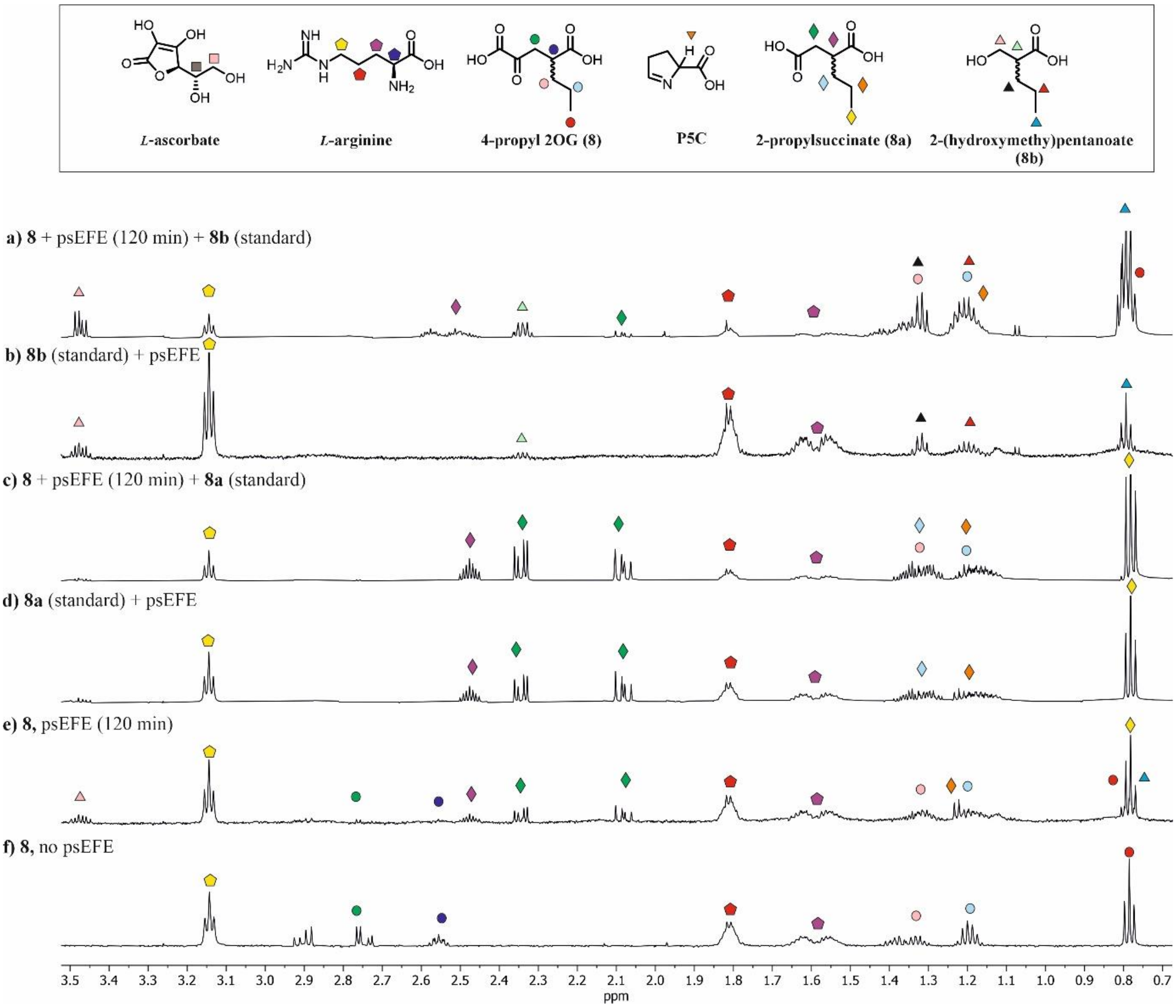

Supporting Figure S32. Analysis of psEFE catalysis using racemic 4-propyl-2OG (8) as a substrate. ^{1}H NMR analysis (~0.7 to ~3.4 ppm) of: **a**) a reaction mixture of psEFE and racemic 4-propyl-2OG (**8**) spiked with 2-(hydroxymethyl)pentanoate (**8a**) 120 min post-incubation with psEFE, **b**) **8a** in the absence of **8** under standard conditions; **c**) a reaction mixture of psEFE and **8** spiked with 2-propylsuccinate (**8b**) 120 min post-incubation with psEFE, **d**) **8b** in the absence of **8** under standard conditions; **e**) a reaction mixture of psEFE and **8** 120 min post addition of psEFE; **f**) **8** under standard conditions in the absence of psEFE. Conditions: 400 µM **8**, 500 µM L-arginine, 500 µM L-ascorbate, 50 µM Fe(II), 800 µM TMSP-d_4, 10 µM psEFE, and, if appropriate, 2 mM of an appropriate authentic standard in buffer (50 mM phosphate, pH 7.4, 10%$_{v/v}$ D_2O). The doublet observed at ~1.2 ppm originates from the enzyme sample; it was observed in the enzyme-only control.

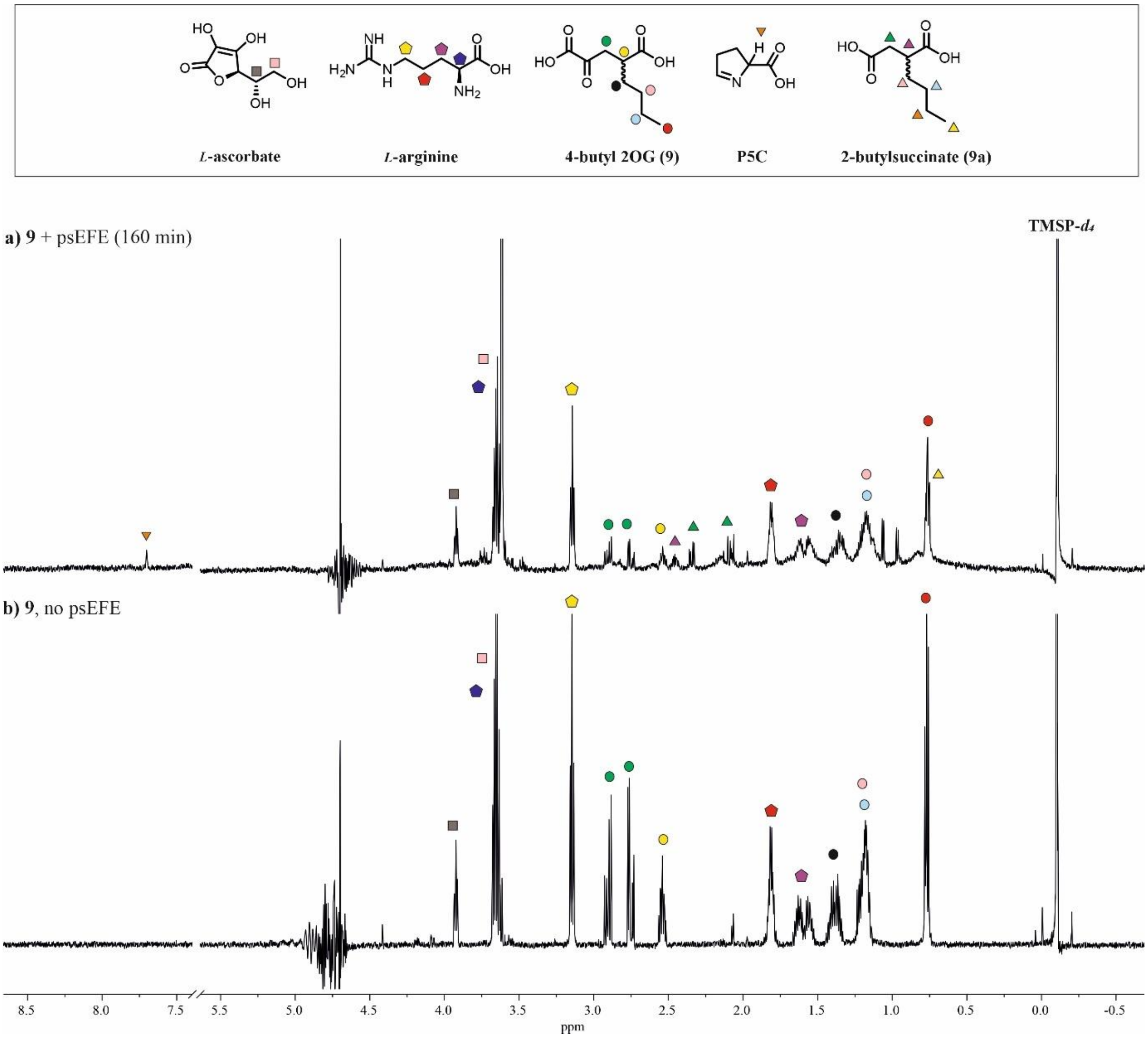

Supporting Figure S33. psEFE catalyses the conversion of *L*-arginine to P5C using racemic 4-butyl-2OG (9) as a cosubstrate. a) ^{1}H NMR analysis of the reaction of psEFE with racemic 4-butyl-2OG (**9**) as a cosubstrate reveals formation of P5C; **b**) ^{1}H NMR spectrum of **9** under standard conditions in the absence of psEFE. Conditions: 400 µM **9**, 500 µM *L*-arginine, 500 µM *L*-ascorbate, 50 µM Fe (II), 800 µM TMSP-d_4, and 10 µM psEFE in buffer (50 mM sodium phosphate, pH 7.4, 10%$_{v/v}$ D_2O). Both the doublet observed at ~1.2 ppm and the singlet observed at ~4.4 ppm originate from the enzyme sample; both signals were observed in the enzyme-only control.

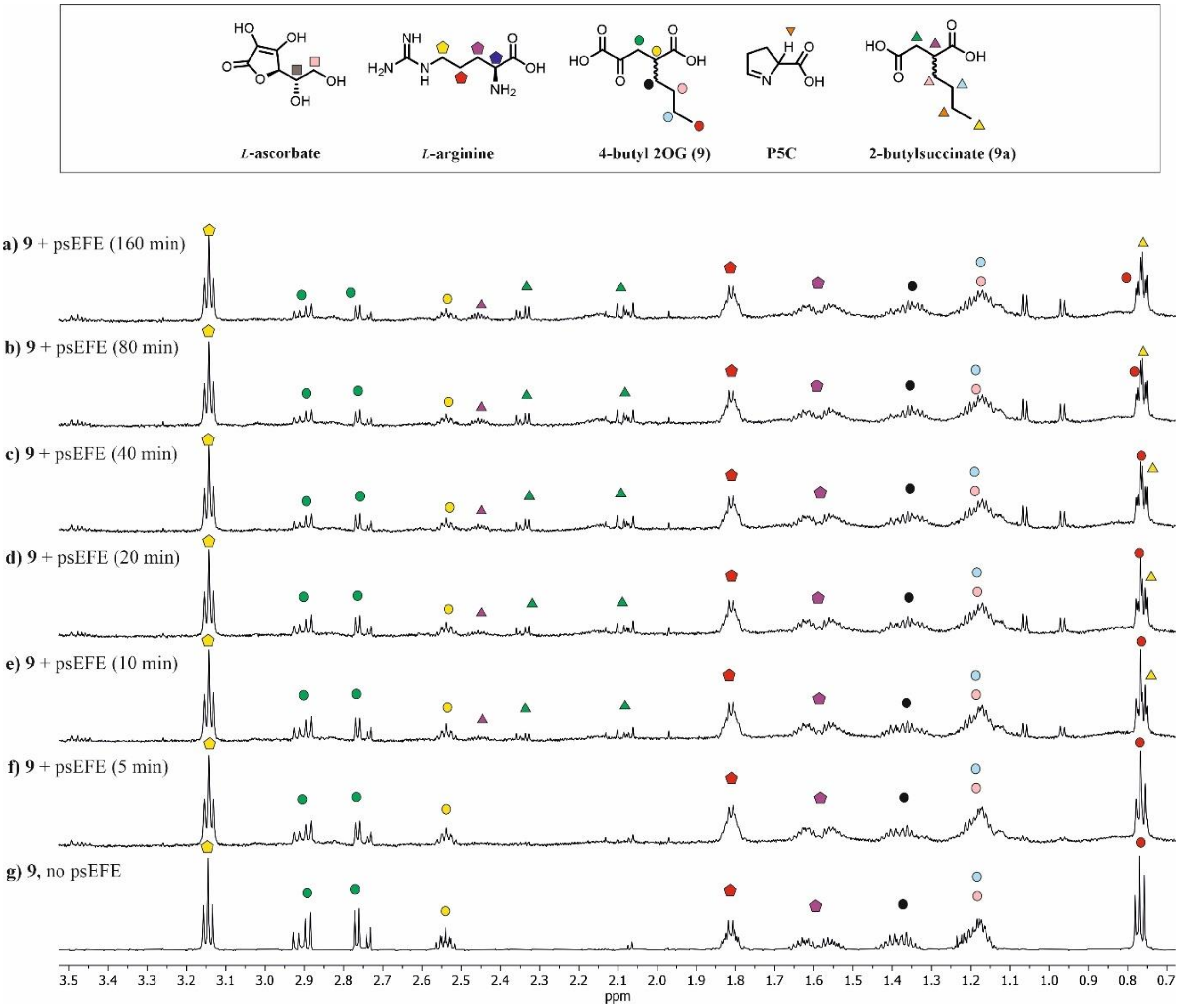

Supporting Figure S34. Time-dependent psEFE-catalyzed conversion of racemic 4-butyl-2OG (9). a-f) Representative ^{1}H NMR spectra (~0.7 to ~3.4 ppm) monitoring the reaction of psEFE with racemic 4-butyl-2OG (**9**) after: **a**) 160 min, **b**) 80 min, **c**) 40 min, **d**) 20 min, **e**) 10 min, and **f**) 5 min. **g**) ^{1}H NMR analysis of the reaction mixture in the absence of psEFE. Conditions: 400 µM **9**, 500 µM *L*-arginine, 500 µM *L*-ascorbate, 50 µM Fe (II), 800 µM TMSP-d_4, and 10 µM psEFE in buffer (50 mM sodium phosphate, pH 7.4, 10%$_{v/v}$ D_2O). Note the two doublets at ~1.07 and ~0.97 ppm increase over time, but do not appear to be a direct product of **9**, because the sum of integrals for **9** and **9a** implies the presence of 400 µM **9** and **9a** (*i.e.*, the starting concentration of **9**) at any given time point during the assay (Figure 5i). The species giving rise to the signals at ~1.07 and ~0.97 ppm, however, has not been assigned.

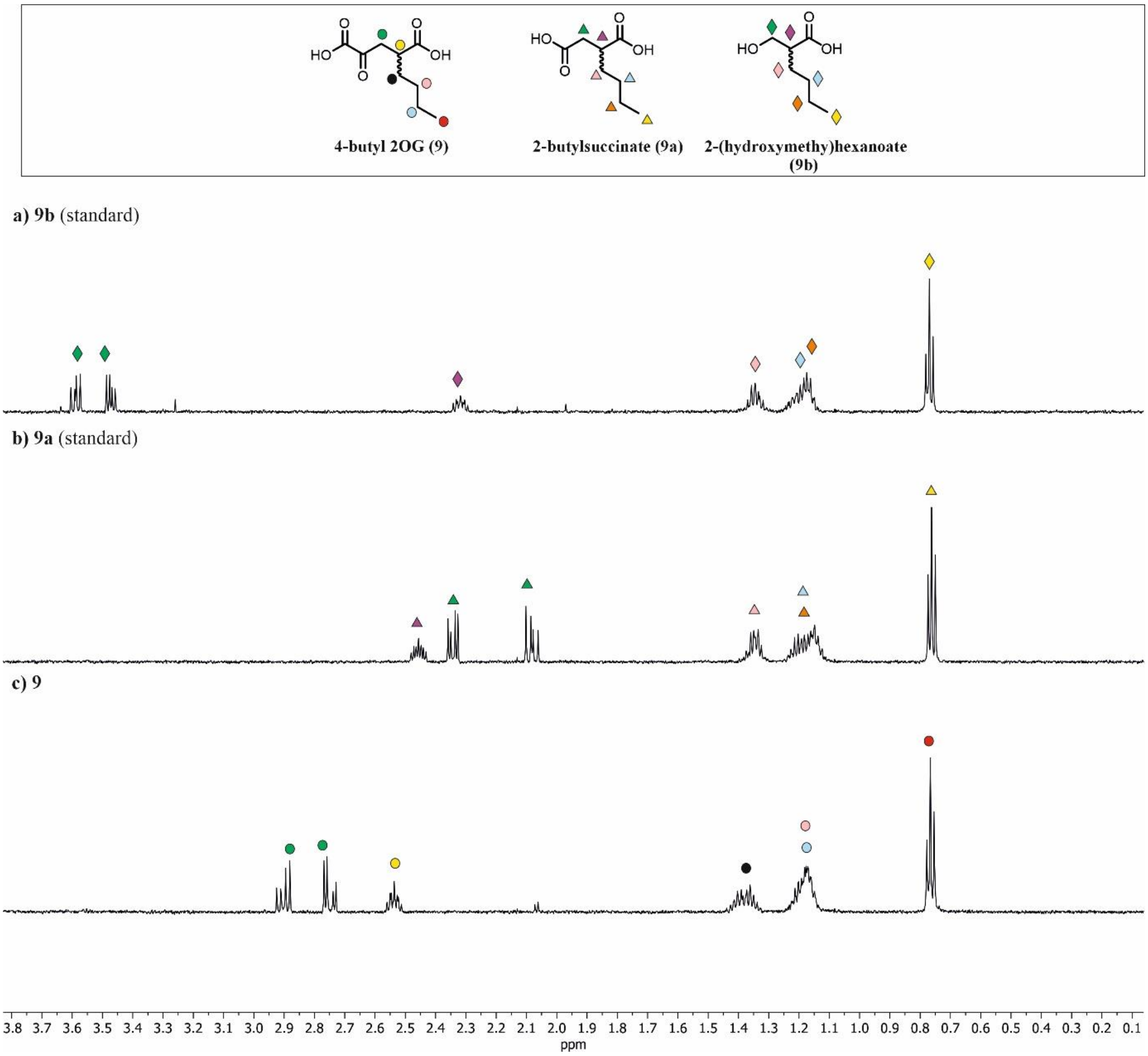

Supporting Figure S35. ^{1}H NMR analysis of racemic 4-butyl-2OG (9) and authentic sample of its products formed during psEFE catalysis. a-c) ^{1}H NMR spectra (~0.4 to ~4.0 ppm) of: **a**) 2-(hydroxymethyl)hexanoate (**9b**), **b)** 2-butylsuccinate (**9a**), and **c**) racemic 4-butyl-2OG (**9**) in buffer (50 mM sodium phosphate, pH 7.4, $10\%_{v/v}$ D_2O).

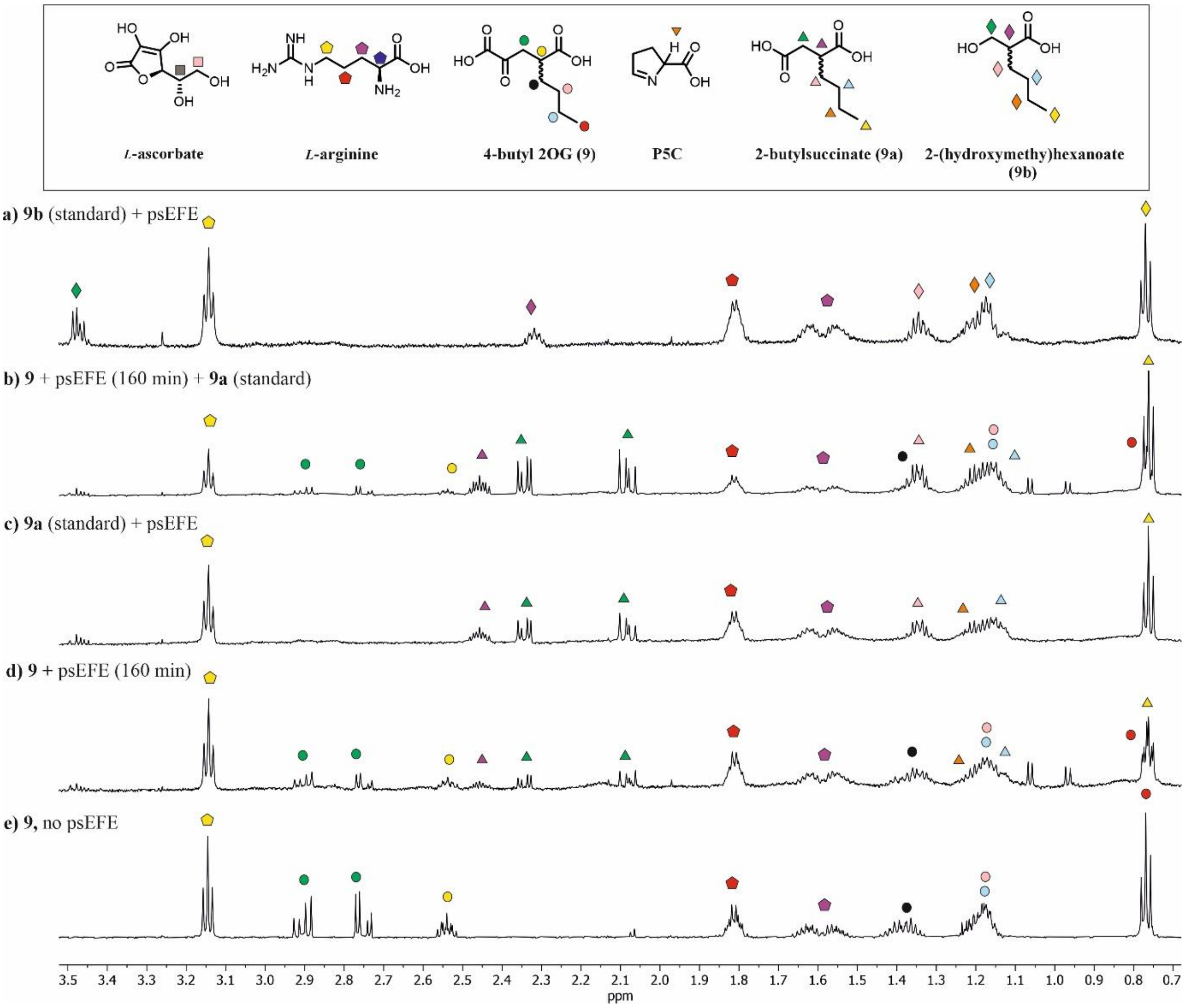

Supporting Figure S36. Analysis of psEFE catalysis using racemic 4-butyl-2OG (9) as a substrate. ^{1}H NMR analysis (~0.7 to ~3.4 ppm) of: **a**) 2-(hydroxymethyl)hexanoate (**9a**) in the absence of racemic 4-butyl-2OG (**9**) under standard conditions, **b)** a reaction mixture of psEFE and **9** spiked with 2-butylsuccinate (**9b**), **c**) **9b** in the absence of **9** under standard conditions; **d**) a reaction mixture of psEFE and **9** 180 min post addition of psEFE; **e**) **9** under standard conditions in the absence of psEFE. Conditions: 400 µM **9**, 500 µM L-arginine, 500 µM L-ascorbate, 50 µM Fe(II), 800 µM TMSP-d_4, 10 µM psEFE, and, if appropriate, 2 mM of an appropriate authentic standard in buffer (50 mM phosphate, pH 7.4, 10%$_{v/v}$ D_2O). Note the two doublets at ~1.07 and ~0.97 ppm increase over time, but do not appear to be a direct product of **9**, because the sum of integrals for **9** and **9a** implies the presence of 400 µM **9** and **9a** (*i.e.*, the starting concentration of **9**) at any given time point during the assay (Figure 5i). The species giving rise to the signals at ~1.07 and ~0.97 ppm, however, has not been assigned. Note that the signals at ~3.5 ppm observed originate from the enzyme sample; it was observed in the enzyme-only control and the integrals do not increase over time (Supporting Figure S34).

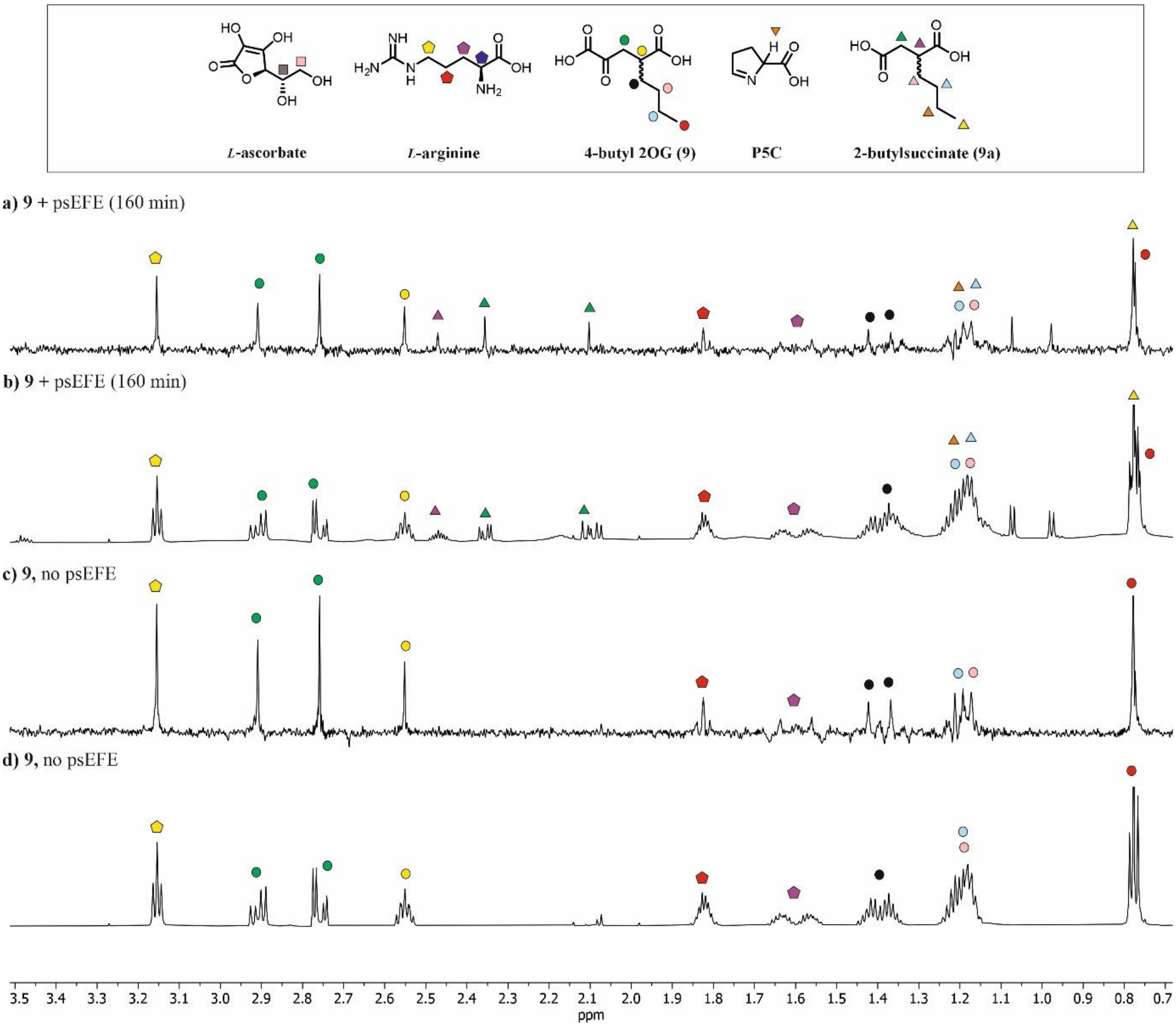

Supporting Figure S37. Analysis of the reaction outcome of psEFE catalyzed conversion of racemic 4-butyl 2OG (9) using pure shift ¹H NMR. Analysis of the reaction of psEFE with racemic 4-butyl 2OG (**9**) 1 h post addition of psEFE using: **a**) pure shift ¹H NMR (1, 5-7) and **b**) ¹H NMR results indicate that substantial amounts of reaction products other than 2-butylsuccinate (**9a**) (Supporting Figures S33-36) are not being formed under the tested conditions. Analysis of the reaction mixture with **9** in the absence of psEFE using: **c**) pure shift ¹H NMR (1, 5-7) and **d**) ¹H NMR. Conditions: 400 µM **9**, 500 µM *L*-arginine, 500 µM *L*-ascorbate, 50 µM Fe(II), 800 µM TMSP-d_4, and 10 µM psEFE in buffer (50 mM sodium phosphate, pH 7.4, 10%$_{v/v}$ D_2O). Note that for the pure shift ¹H NMR analysis, the observed peak may not necessarily be at the center of the peak obtained when using standard NMR analysis (1, 5-7). The singlet observed at ~4.4 ppm originates from the enzyme sample; it was observed in the enzyme-only control. Note the two doublets at ~1.07 and ~0.97 ppm increase over time, but do not appear to be a direct product of **9**, because the sum of integrals for **9** and **9a** implies the presence of 400 µM **9** and **9a** (*i.e.*, the starting concentration of **9**) at any given time point during the assay (Figure 5i). The species giving rise to the signals at ~1.07 and ~0.97 ppm, however, has not been assigned.

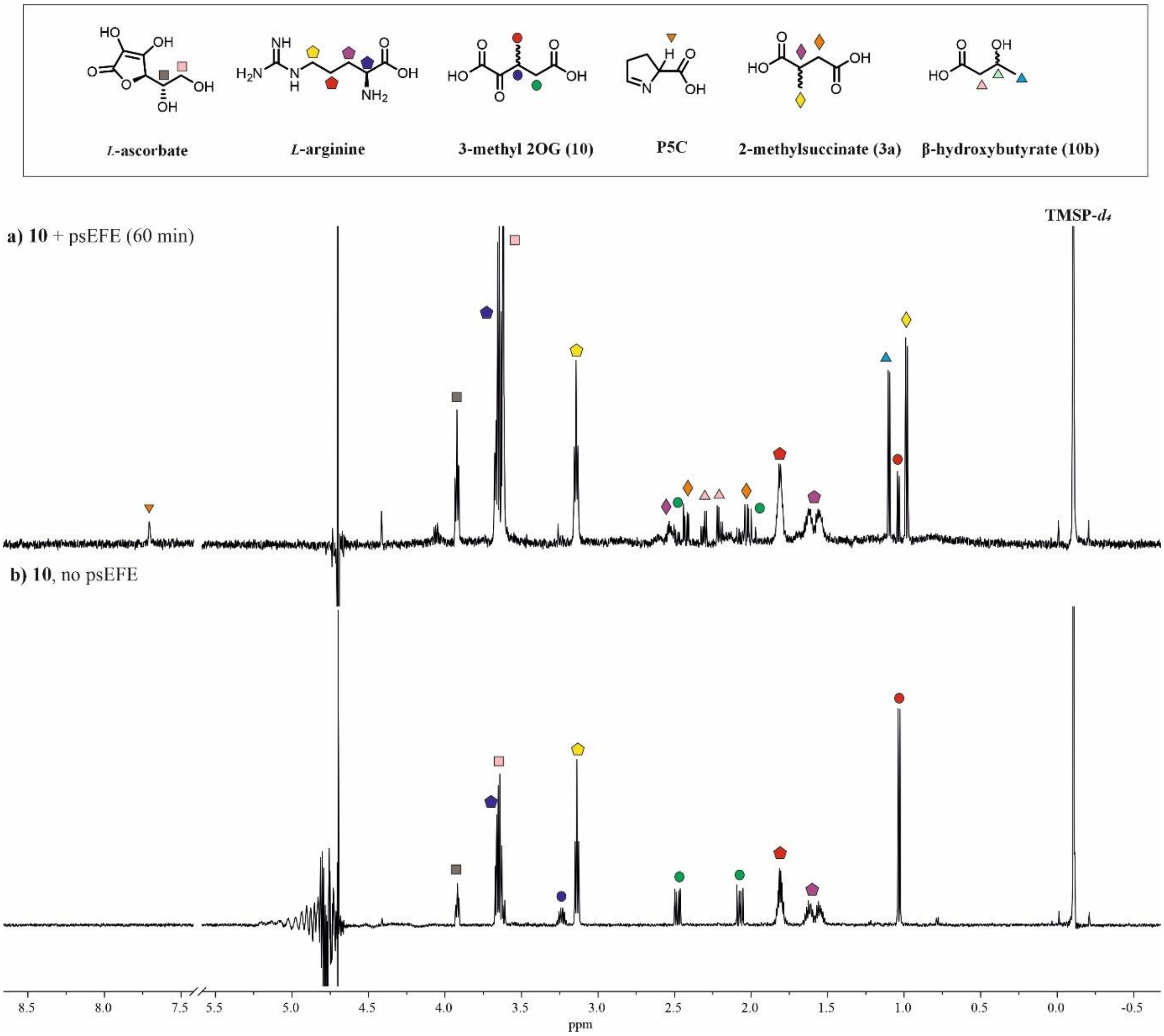

Supporting Figure S38. psEFE catalyzes the conversion of *L*-arginine to P5C using racemic 3-methyl-2OG (10) as a cosubstrate. a) ^{1}H NMR analysis of the reaction of psEFE with racemic 3-methyl-2OG (**10**) as a cosubstrate reveals formation of P5C; **b**) ^{1}H NMR spectrum of **10** under standard conditions in the absence of psEFE. Conditions: 400 µM **10**, 500 µM *L*-arginine, 500 µM *L*-ascorbate, 50 µM Fe (II), 800 µM TMSP-d_4, and 10 µM psEFE in buffer (50 mM sodium phosphate, pH 7.4, 10%$_{v/v}$ D_2O). The singlet observed at ~4.4 ppm originates from the enzyme sample; it was observed in the enzyme-only control.

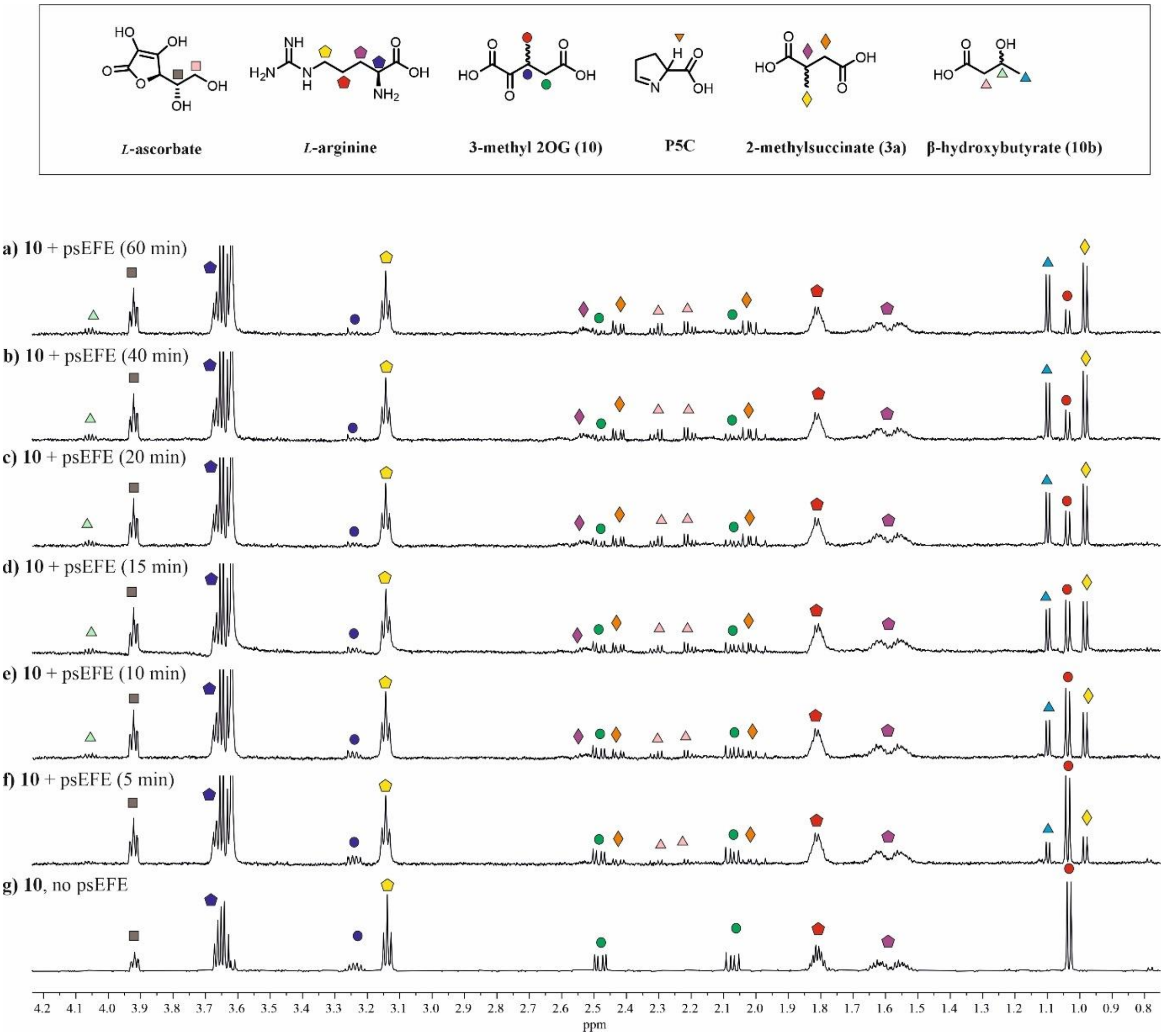

Supporting Figure S39. Time-dependent psEFE-catalyzed conversion of racemic 3-methyl-2OG (10). a-f) Representative ^{1}H NMR spectra (~0.8 to ~4.2 ppm) monitoring the reaction of psEFE with racemic 3-methyl-2OG (**10**) after: **a**) 60 min, **b**) 40 min, **c**) 20 min, **d**) 15 min, **e**) 10 min, and **f**) 5 min. **g**) ^{1}H NMR analysis of the reaction mixture in the absence of psEFE. Conditions: 400 µM **10**, 500 µM *L*-arginine, 500 µM *L*-ascorbate, 50 µM Fe (II), 800 µM TMSP-d_4, and 10 µM psEFE in buffer (50 mM sodium phosphate, pH 7.4, 10%$_{v/v}$ D_2O).

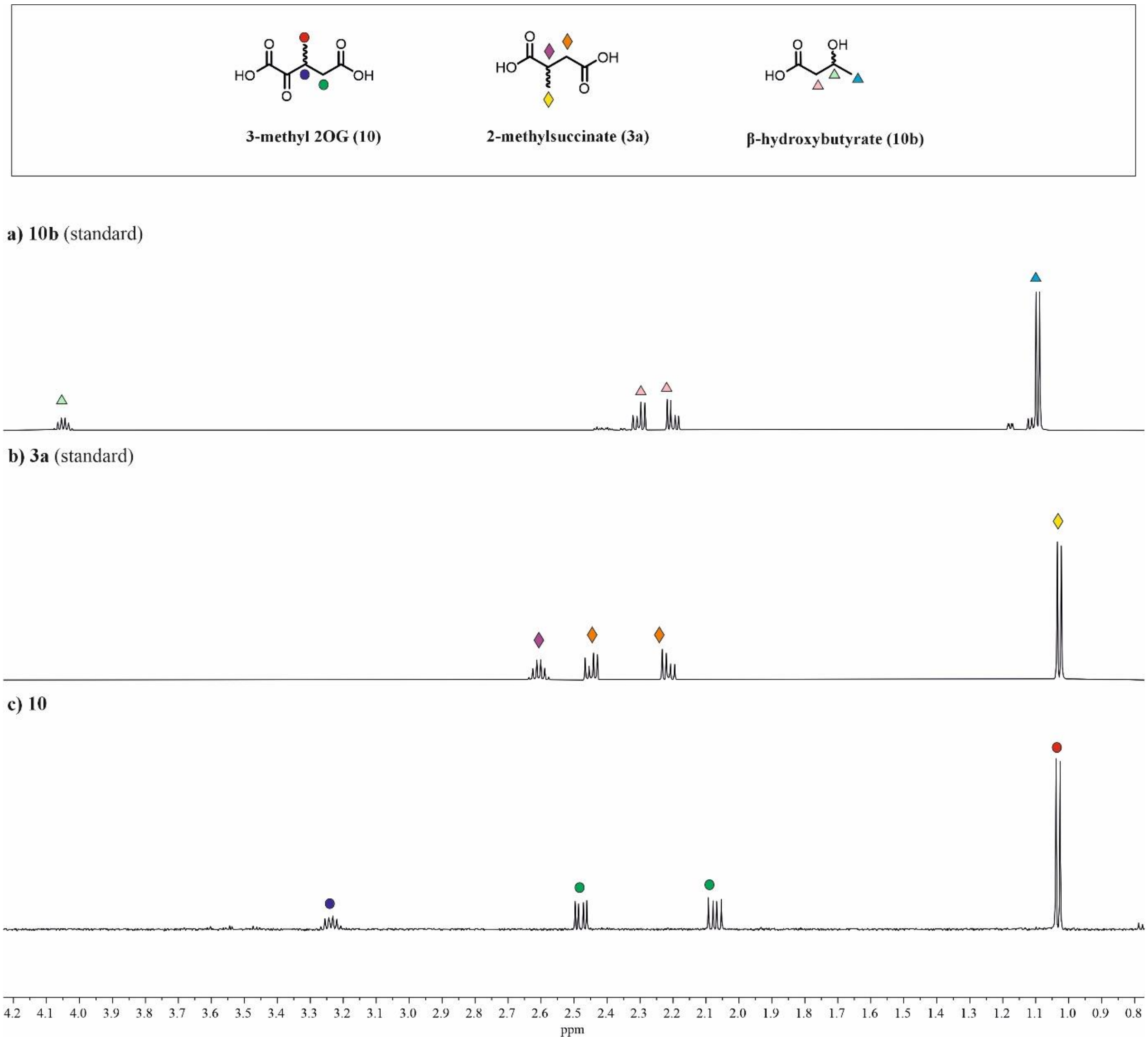

Supporting Figure S40. ^{1}H NMR analysis of racemic 3-methyl-2OG (10) and authentic samples of its products formed during psEFE catalysis. a-c) ^{1}H NMR spectra (~0.8 to ~4.2 ppm) of: **a**) β-hydroxybutyrate (**10b**), **b**) 2-methylsuccinate (**3a**), and **c**) racemic 3-methyl-2OG (**10**) in buffer (50 mM sodium phosphate, pH 7.4, 10%$_{v/v}$ D_2O).

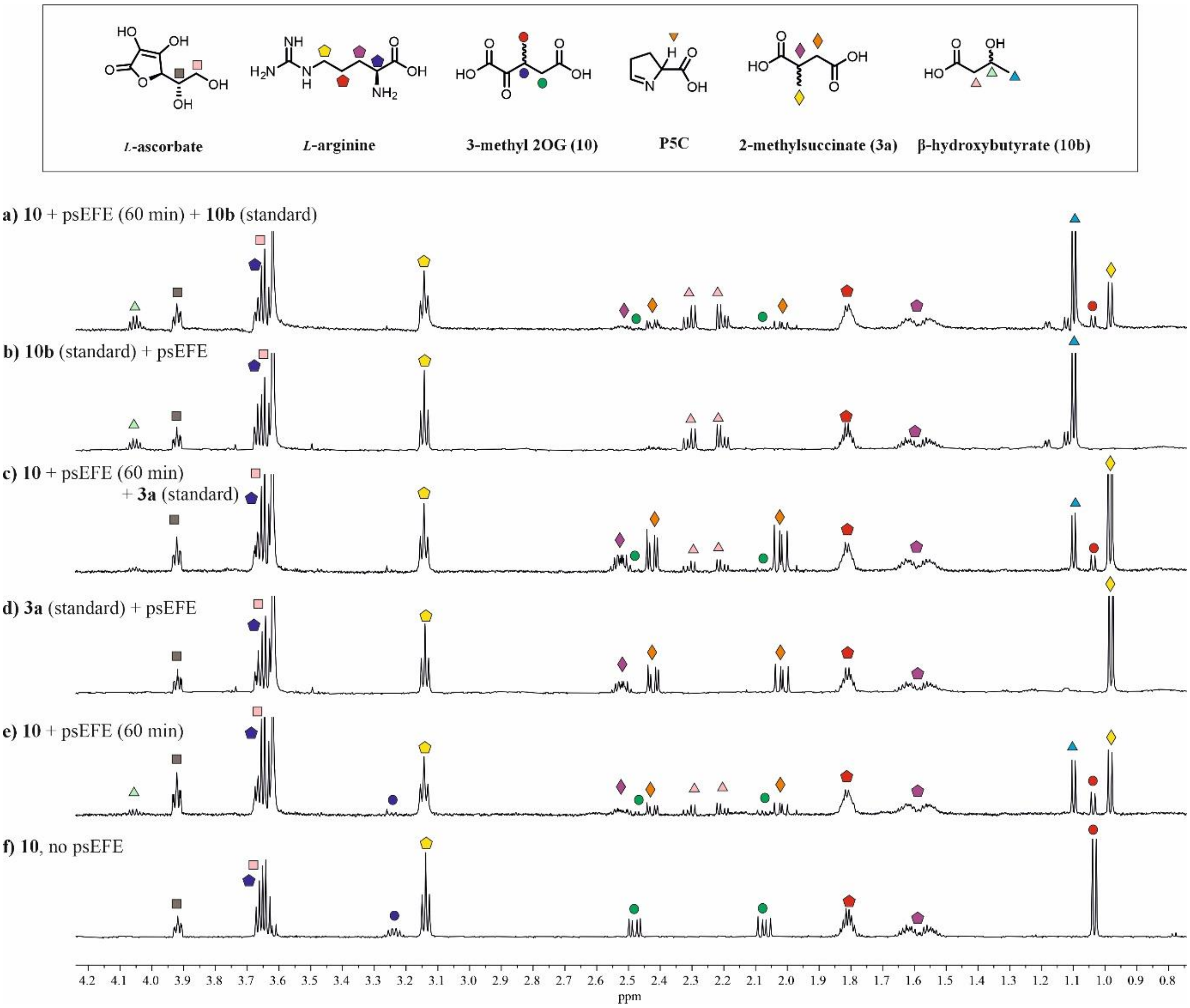

Supporting Figure S41. Analysis of psEFE catalysis using racemic 3-methyl-2OG (10) as a substrate. ^{1}H NMR analysis (~0.8 to ~4.2 ppm) of: **a**) a reaction mixture of psEFE and racemic 3-methyl-2OG (**10**) spiked with β-hydroxybutyrate (**10b**), **b**) **10b** in the absence of **10** under standard conditions; **c**) a reaction mixture of psEFE and **10** spiked with 2-methylsuccinate (**3a**), **d**) **3a** in the absence of **10** under standard conditions; **e**) a reaction mixture of psEFE and **10** 100 min post addition of psEFE; **f**) **10** under standard conditions in the absence of psEFE. Conditions: 400 µM **10**, 500 µM L-arginine, 500 µM L-ascorbate, 50 µM Fe(II), 800 µM TMSP-d_4, 10 µM psEFE, and, if appropriate, 2 mM of an appropriate authentic standard in buffer (50 mM phosphate, pH 7.4, 10%$_{v/v}$ D_2O).

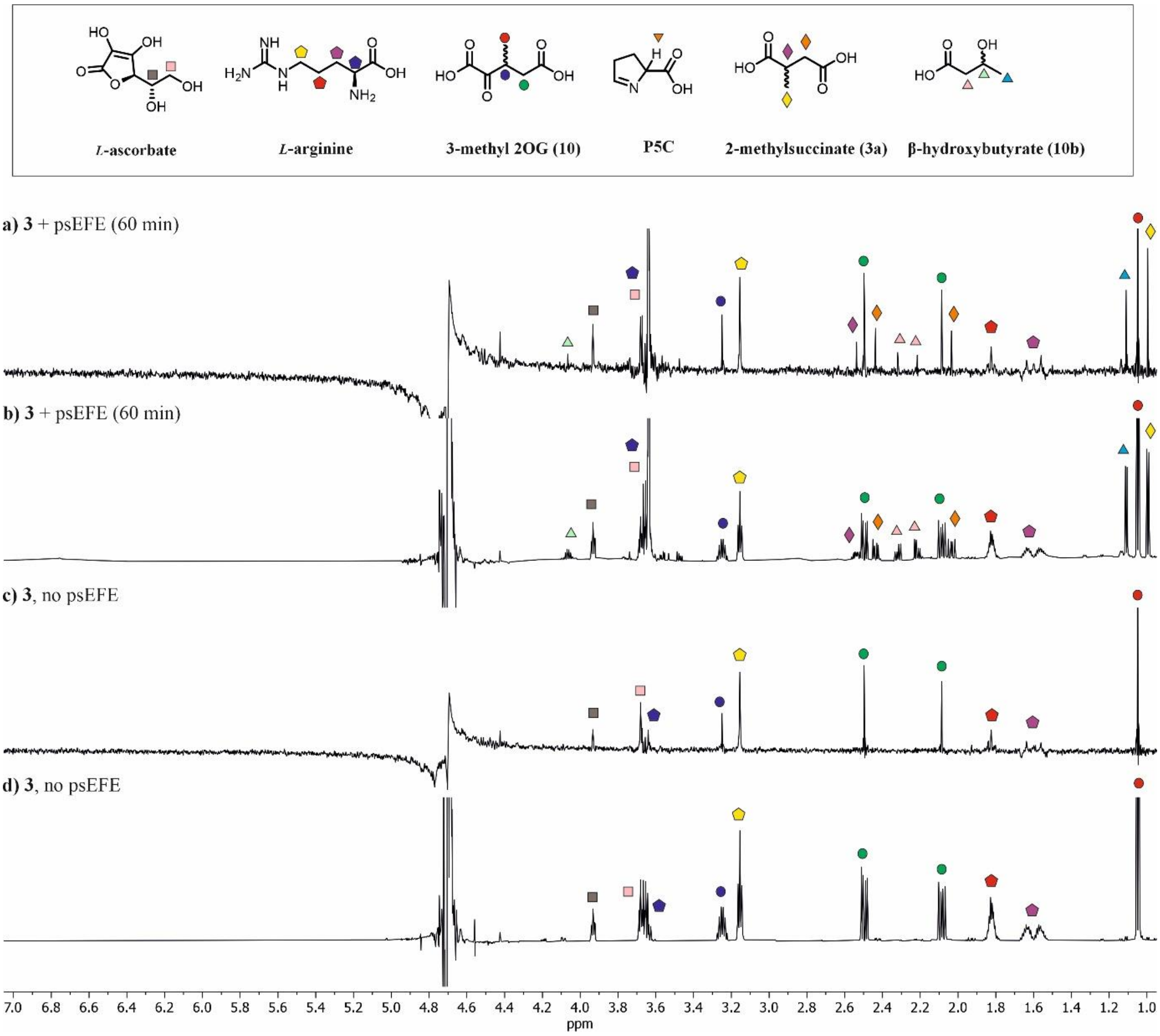

Supporting Figure S42. Analysis of the reaction outcome of psEFE catalysed conversion of racemic 3-methyl-2OG (10) using pure shift ^{1}H NMR. Analysis of the reaction of psEFE with racemic 3-methyl-2OG (**10**) 30 min post addition of psEFE using: **a**) pure shift ^{1}H NMR (1, 5-7) and **b**) ^{1}H NMR results indicate that substantial amounts of reaction products other than β-hydroxyisobutyrate (**10b**), 2-methylsuccinate (**3a**), and P5C (Supporting Figures S38-41) are not being formed under the tested conditions. Analysis of the reaction mixture with **10** in the absence of psEFE using: **c**) pure shift ^{1}H NMR (1, 5-7) and **d**) ^{1}H NMR. Conditions: 400 µM **10**, 500 µM *L*-arginine, 500 µM *L*-ascorbate, 50 µM Fe (II), 800 µM TMSP-d_4, and 10 µM psEFE in buffer (50 mM sodium phosphate, pH 7.4, 10%$_{v/v}$ D_2O). Note that for the pure shift ^{1}H NMR analysis, the observed peak may not necessarily be at the center of the peak obtained when using standard NMR analysis (1, 5-7). The singlet observed at ~4.4 ppm originates from the enzyme sample; it was observed in the enzyme-only control.

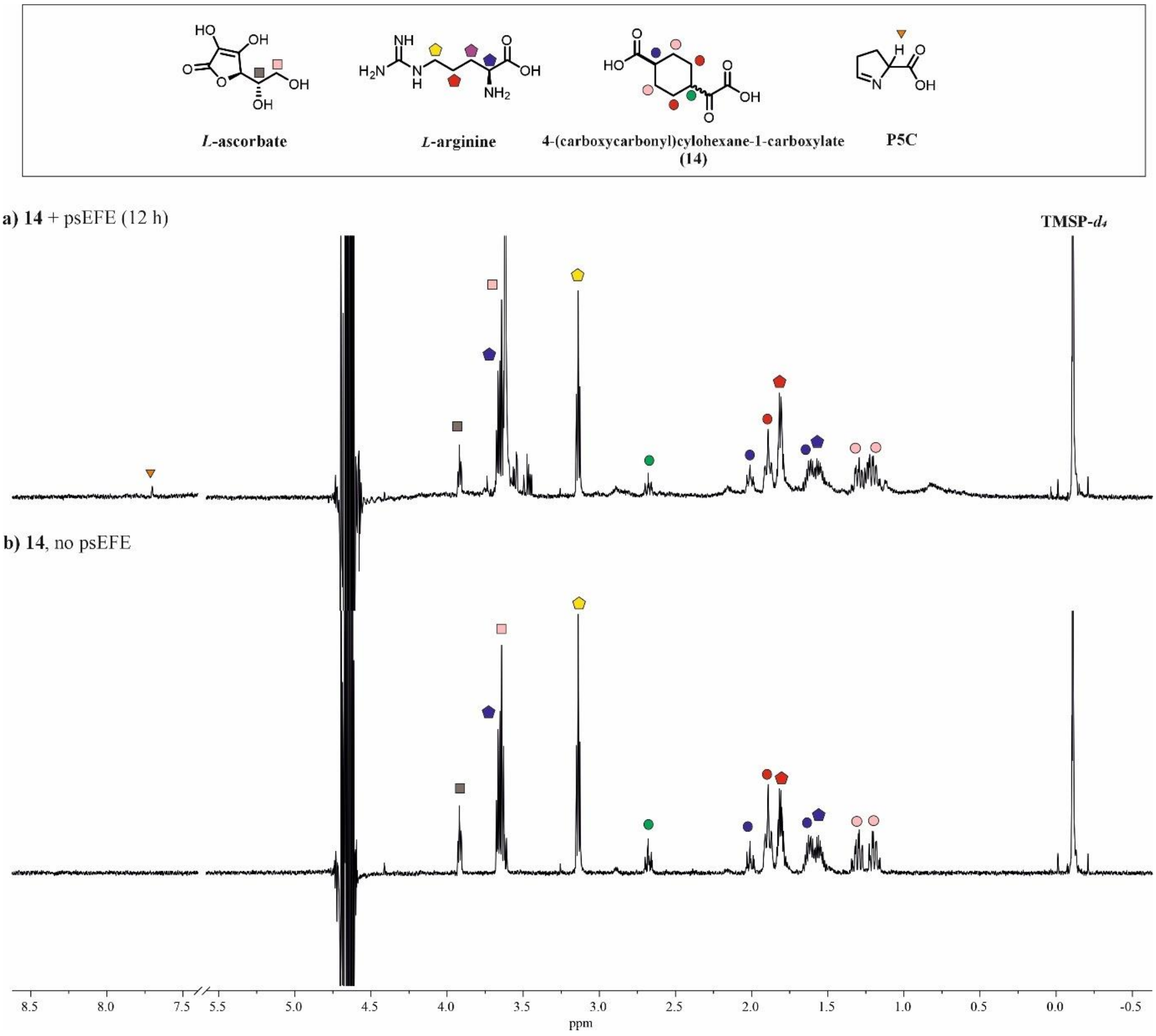

Supporting Figure S43. psEFE catalyzes the conversion of *L*-arginine to P5C using 4-(carboxycarbonyl)cyclohexane-1-carboxylate (14) as a cosubstrate. a) ^{1}H NMR analysis of the reaction of psEFE with 4-(carboxycarbonyl)cyclohexane-1-carboxylate (**14**) as a cosubstrate reveals formation of P5C; **b**) ^{1}H NMR spectrum of **14** under standard conditions in the absence of psEFE. Conditions: 400 µM **14**, 500 µM *L*-arginine, 500 µM *L*-ascorbate, 50 µM Fe (II), 800 µM TMSP-d_4, and 30 µM psEFE in buffer (50 mM sodium phosphate, pH 7.4, 10%$_{v/v}$ D_2O). **14** was used as a mixture of diastereomers, dr (*trans*:*cis*) = 5:1. Both the doublet observed at ~1.2 ppm and the singlet observed at ~4.4 ppm originate from the enzyme sample; both signals were observed in the enzyme-only control.

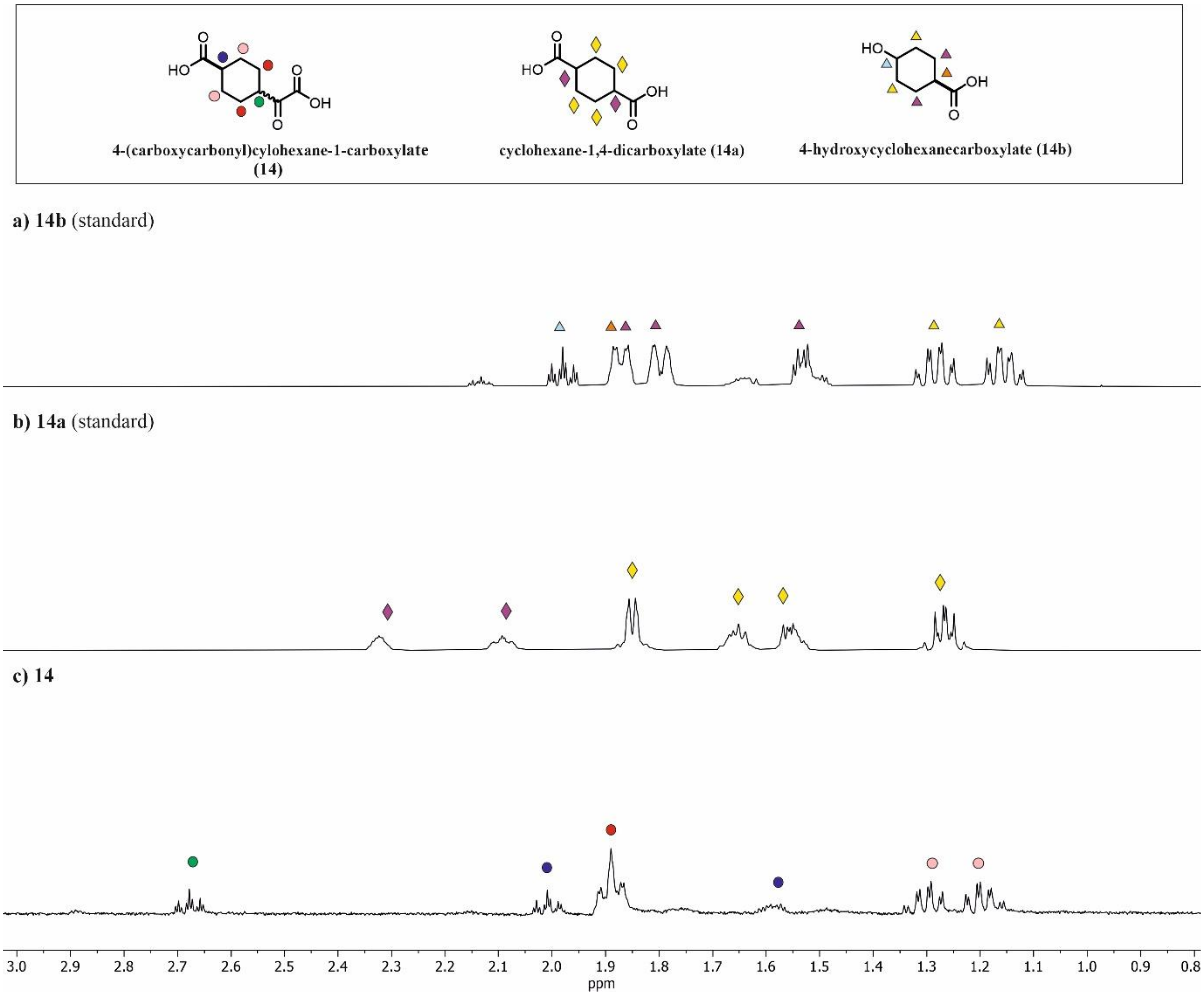

Supporting Figure S44. ^{1}H NMR analysis of 4-(carboxycarbonyl)cyclohexane-1-carboxylic acid (14) and authentic samples of its products formed during psEFE catalysis. a-c) ^{1}H NMR spectra (~0.8 to ~3.0 ppm) of: **a**) a *cis*/*trans* isomeric mixture of 4-hydroxycyclohexanecarboxylate (**14b**), **b**) a *cis*/*trans* isomeric mixture of cyclohexane-1,4-dicarboxylate (**14a**), and **c**) 4-(carboxycarbonyl)cyclohexane-1-carboxylate (**14**) in buffer (50 mM sodium phosphate, pH 7.4, 10%$_{v/v}$ D_2O). **14** was used as a mixture of diastereomers, dr (*trans*:*cis*) = 5:1 (2).

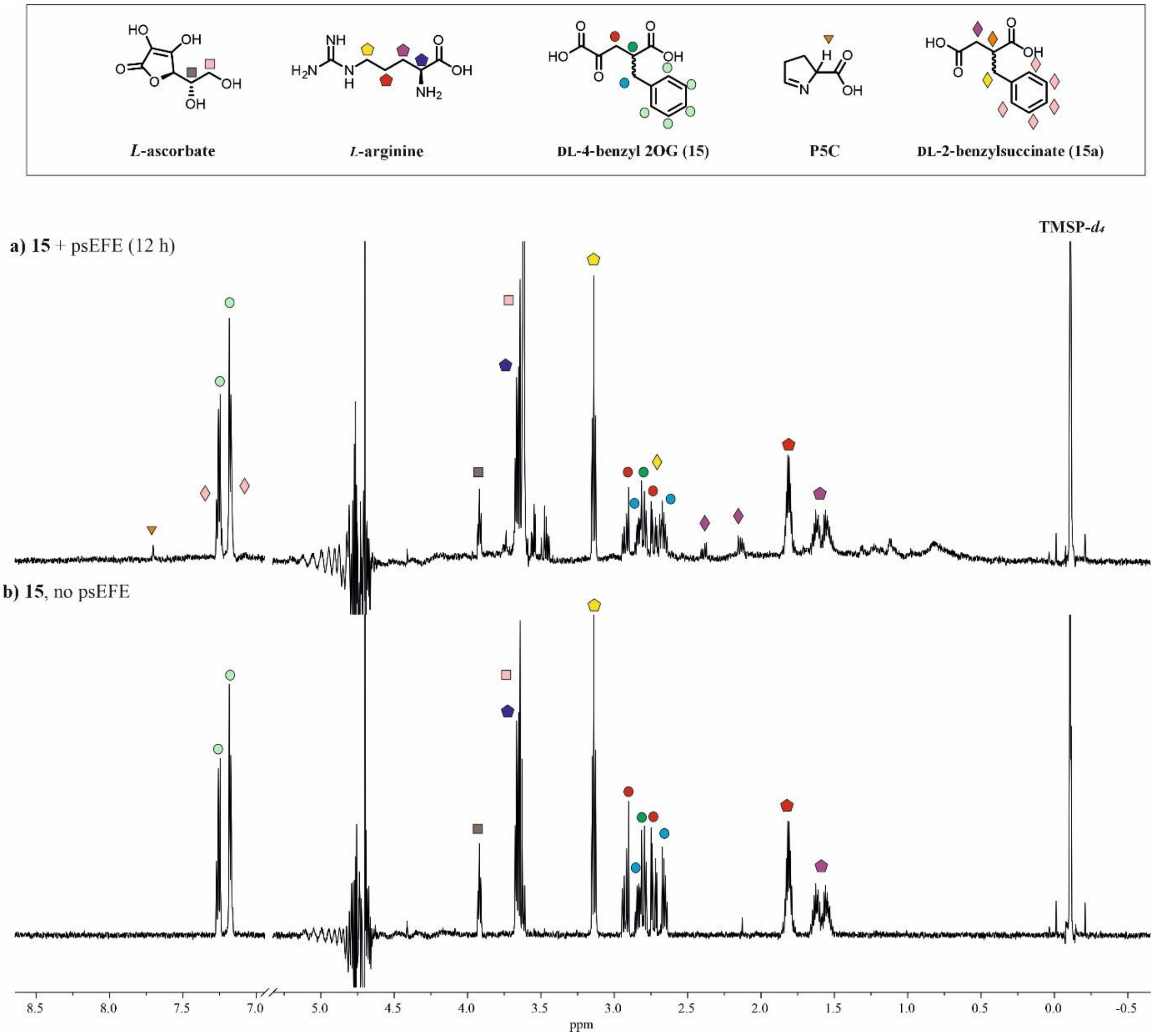

Supporting Figure S45. psEFE catalyzes the conversion of *L*-arginine to P5C using racemic 4-benzyl-2OG (15) as a cosubstrate. a) ^{1}H NMR analysis of the reaction of psEFE with racemic 4-benzyl-2OG (**15**) as a cosubstrate reveals formation of P5C; **b**) ^{1}H NMR spectrum of **15** under standard conditions in the absence of psEFE. Conditions: 400 µM **15**, 500 µM *L*-arginine, 500 µM *L*-ascorbate, 50 µM Fe (II), 800 µM TMSP-d_4, and 30 µM psEFE in buffer (50 mM sodium phosphate, pH 7.4, 10%$_{v/v}$ D_2O). Both the doublet observed at ~1.2 ppm and the singlet observed at ~4.4 ppm originate from the enzyme sample; both signals were observed in the enzyme-only control.

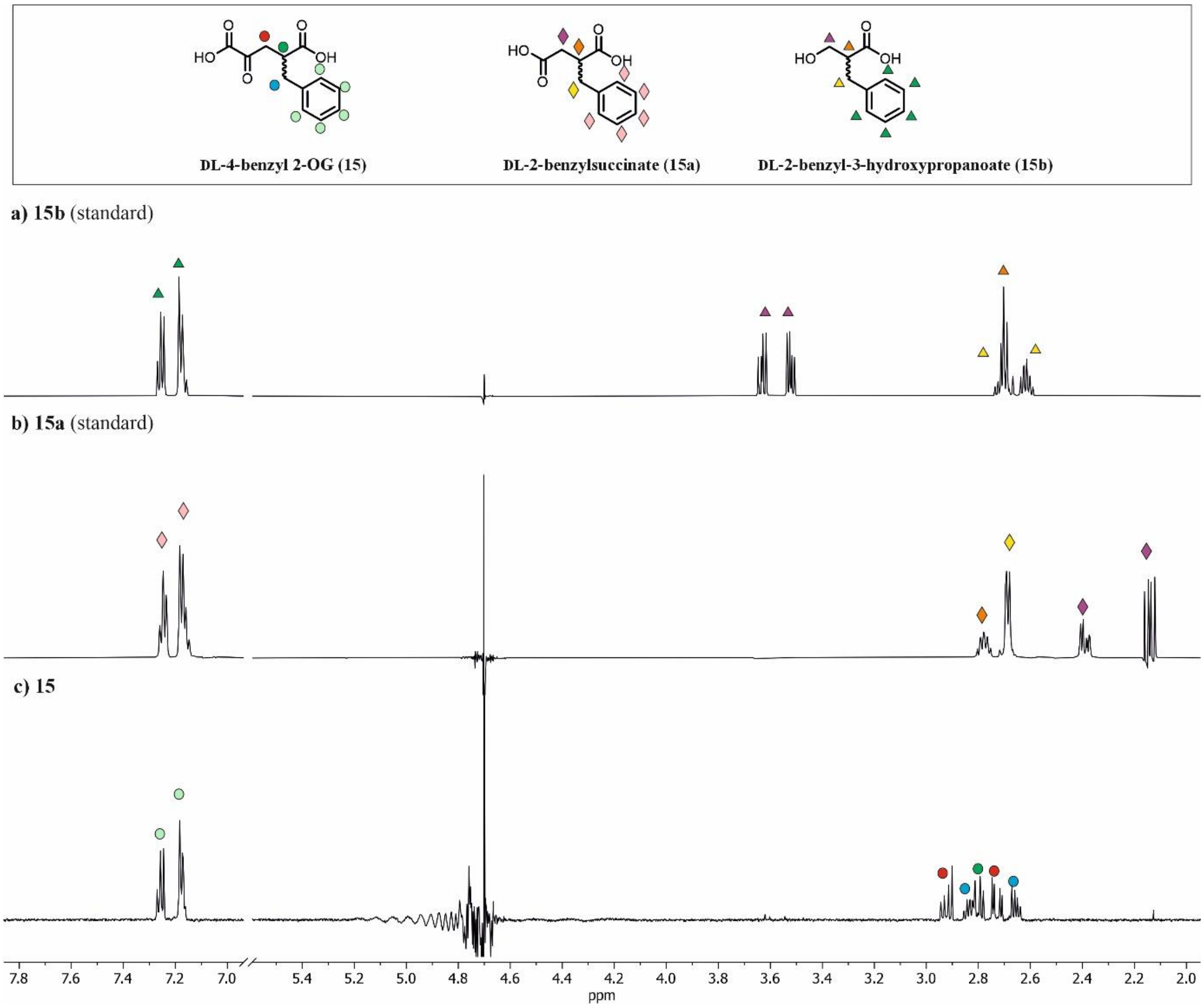

Supporting Figure S46. ^{1}H NMR analysis of racemic 4-benzyl-2OG (15) and authentic samples of its products formed during psEFE catalysis. a-c) ^{1}H NMR spectra (~2.0 to ~7.8 ppm) of: **a**) racemic 2-benzyl-3-hydroxypropanoate (**15b**), **b**) racemic 2-benzylsuccinate (**15a**), and **c**) racemic 4-benzyl-2OG (**15**) in buffer (50 mM sodium phosphate, pH 7.4, 10%$_{v/v}$ D_2O).

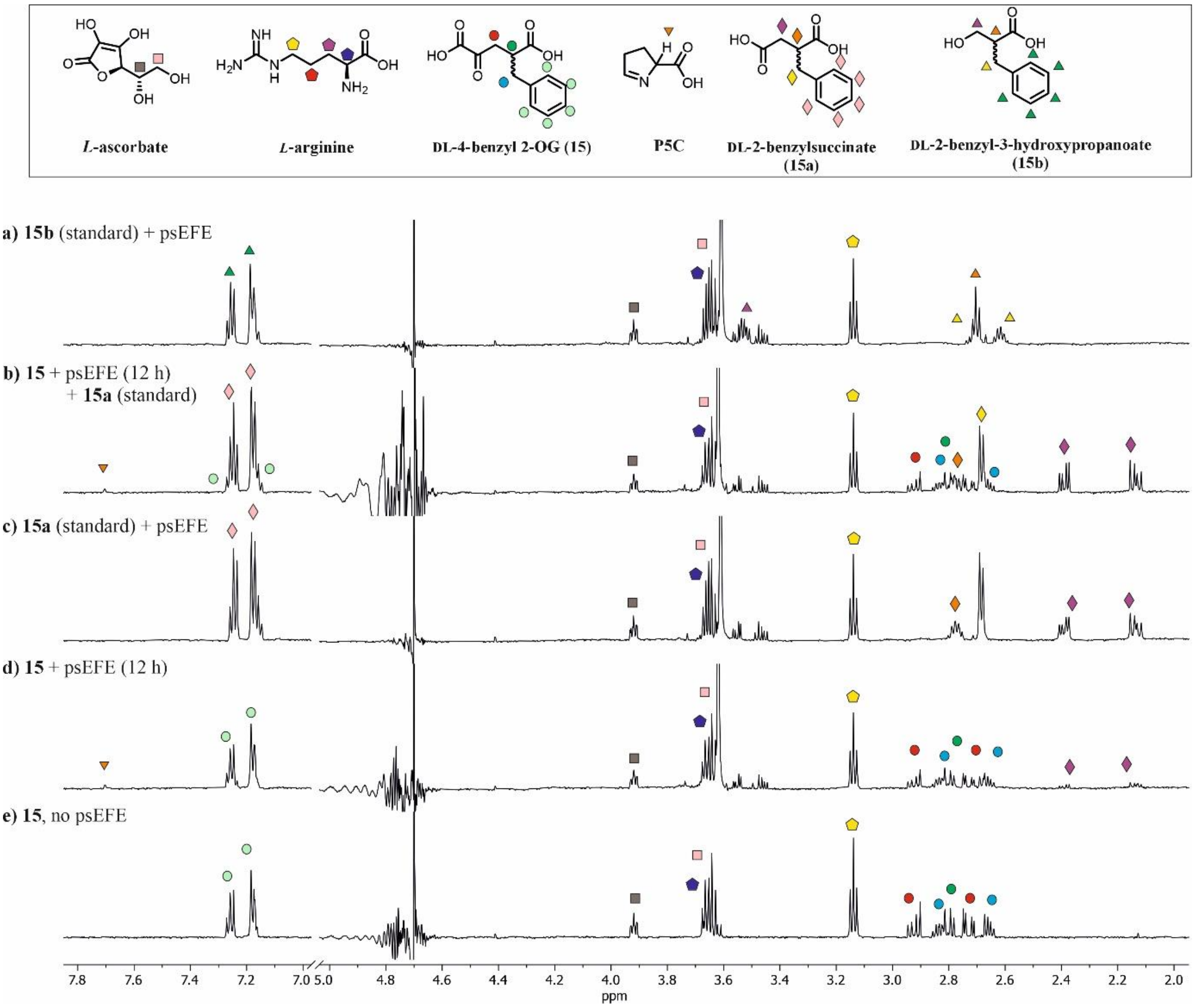

Supporting Figure S47. Analysis of psEFE catalysis using racemic 4-benzyl-2OG (15) as a substrate. ^{1}H NMR analysis (~2.0 to ~7.8 ppm) of: **a**) racemic 2-benzyl-3-hydroxypropanoate (**15b**) in the absence of racemic 4-benzyl-2OG (**15**) under standard conditions, **b**) a reaction mixture of psEFE and **15** spiked with racemic 2-benzylsuccinate (**15a**), **c**) **15a** in the absence of **15** under standard conditions; **d**) a reaction mixture of psEFE and **15** 12 h post addition of psEFE; **e**) **15** under standard conditions in the absence of psEFE. Conditions: 400 µM **15**, 500 µM L-arginine, 500 µM L-ascorbate, 50 µM Fe(II), 800 µM TMSP-d_4, 30 µM psEFE, and, if appropriate, 2 mM of an appropriate authentic standard in buffer (50 mM phosphate, pH 7.4, 10%$_{v/v}$ D_2O). The singlet observed at ~4.4 ppm originates from the enzyme sample; it was observed in the enzyme-only control.

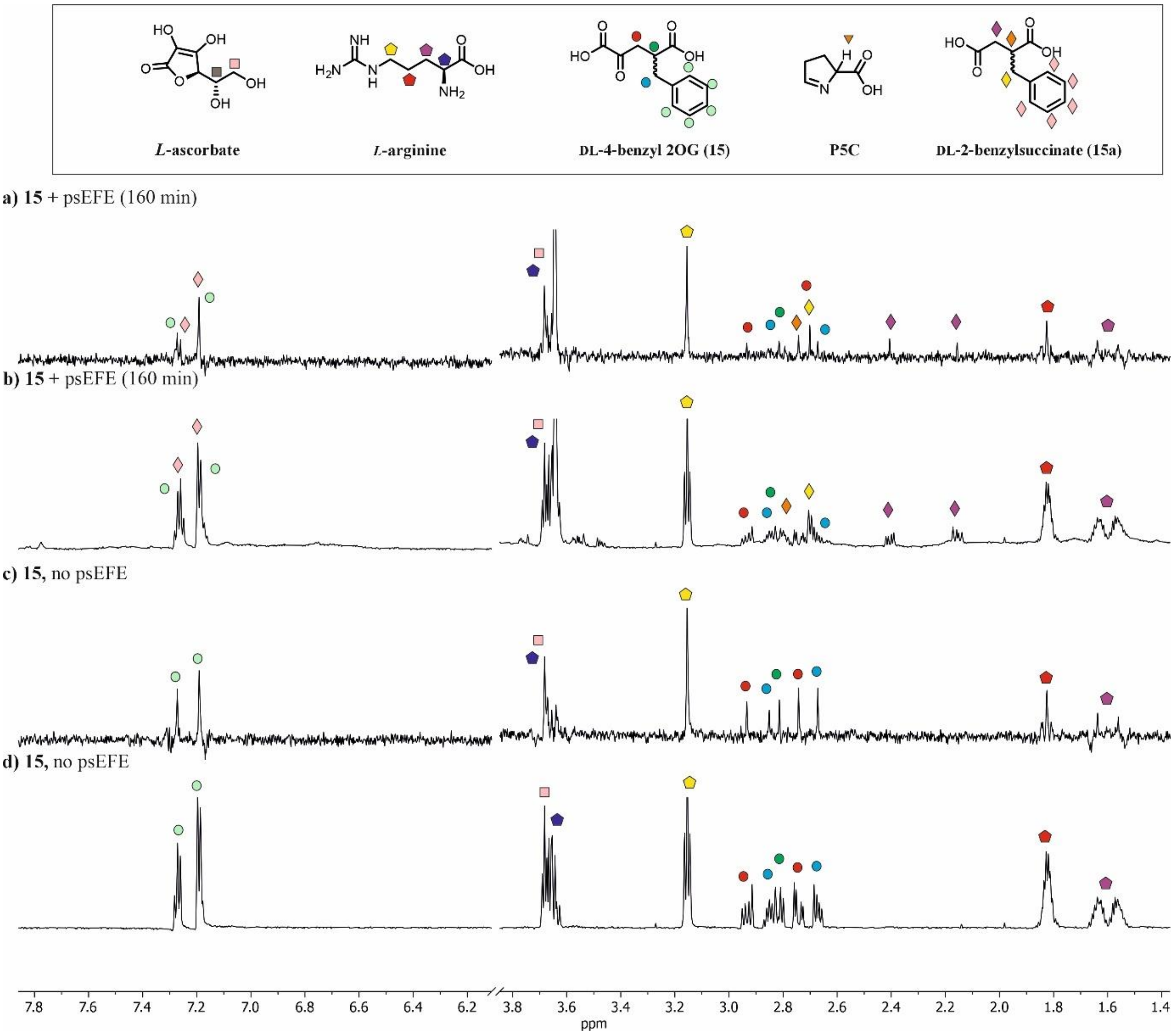

Supporting Figure S48. Analysis of the reaction outcome of psEFE catalyzed conversion of racemic 4-benzyl 2OG (15) using pure shift ^{1}H NMR. Analysis of the reaction of psEFE with racemic 4-benzyl 2OG (**15**) 30 min post addition of psEFE using: **a**) pure shift ^{1}H NMR (1, 5-7) and **b**) ^{1}H NMR results indicate that substantial amounts of reaction products other than 2-benzylsuccinate and P5C (Supporting Figures S44-46) are not being formed under the tested conditions. Analysis of the reaction mixture with **15** in the absence of psEFE using: **c**) pure shift ^{1}H NMR (1, 5-7) and **d**) ^{1}H NMR. Conditions: 400 µM **15**, 500 µM *L*-arginine, 500 µM *L*-ascorbate, 50 µM Fe (II), 800 µM TMSP-d_4, and 30 µM psEFE in buffer (50 mM sodium phosphate, pH 7.4, 10%$_{v/v}$ D_2O). Note that for the pure shift ^{1}H NMR analysis, the observed peak may not necessarily be at the center of the peak obtained when using standard NMR analysis (1, 5-7).

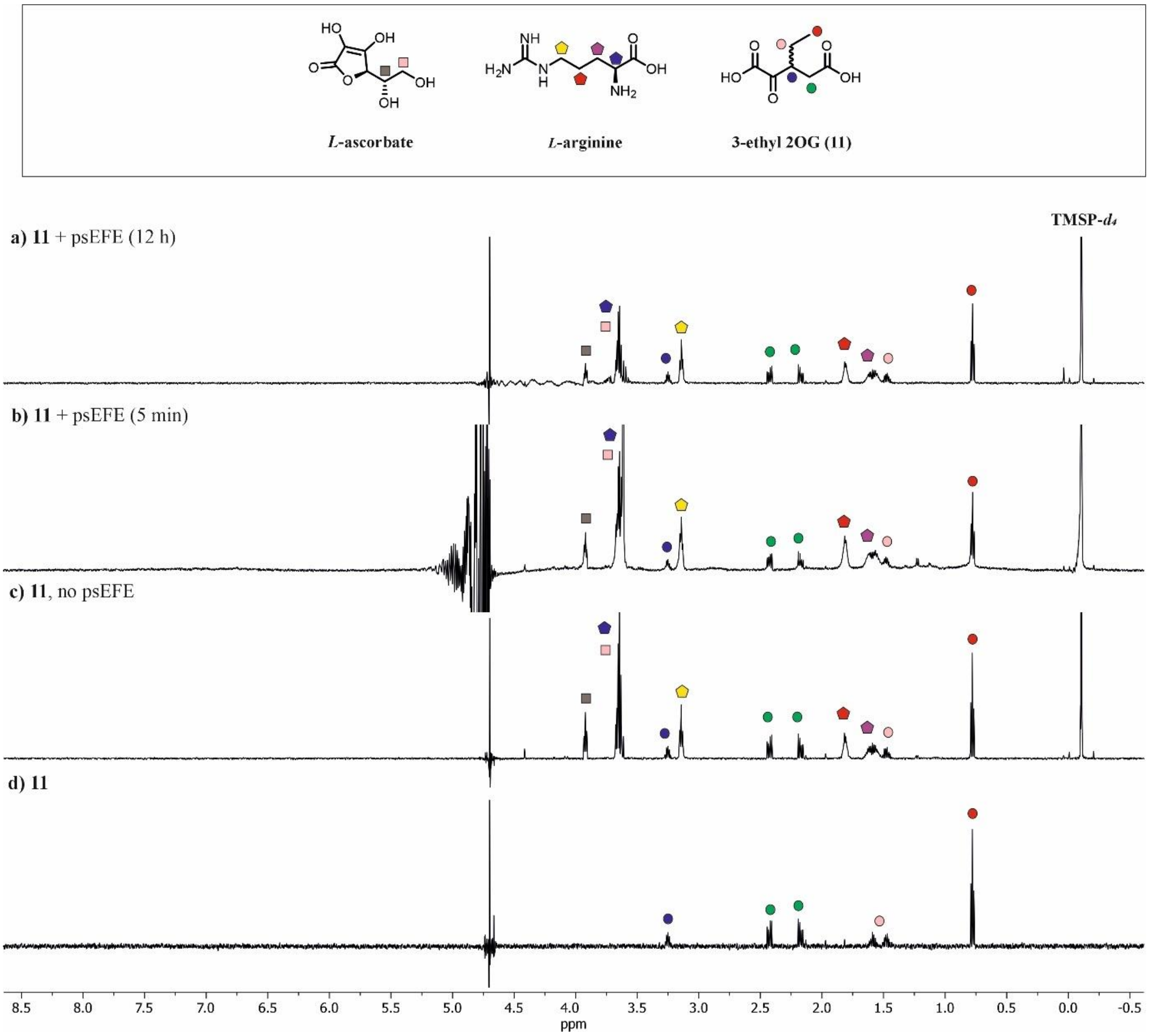

Supporting Figure S49. Analysis of psEFE catalysis using racemic 3-ethyl-2OG (11) as a potential substrate. Representative ^{1}H NMR spectra monitoring the reaction of psEFE with racemic 3-ethyl-2OG (**11**) after: **a**) overnight incubation and **b**) 5 min. **c**) ^{1}H NMR spectrum of **11** under standard conditions in the absence of psEFE; **d**) ^{1}H NMR spectrum of **11** in buffer. Conditions: 400 µM **11**, 500 µM *L*-arginine, 500 µM *L*-ascorbate, 50 µM Fe (II), 800 µM TMSP-d_4, and 30 µM psEFE in buffer (50 mM sodium phosphate, pH 7.4, 11%$_{v/v}$ D_2O). Formation of new peaks was not observed, even after incubating the reaction mixture overnight. Both the doublet observed at ~1.2 ppm and the singlet observed at ~4.4 ppm originate from the enzyme sample; both signals were observed in the enzyme-only control.

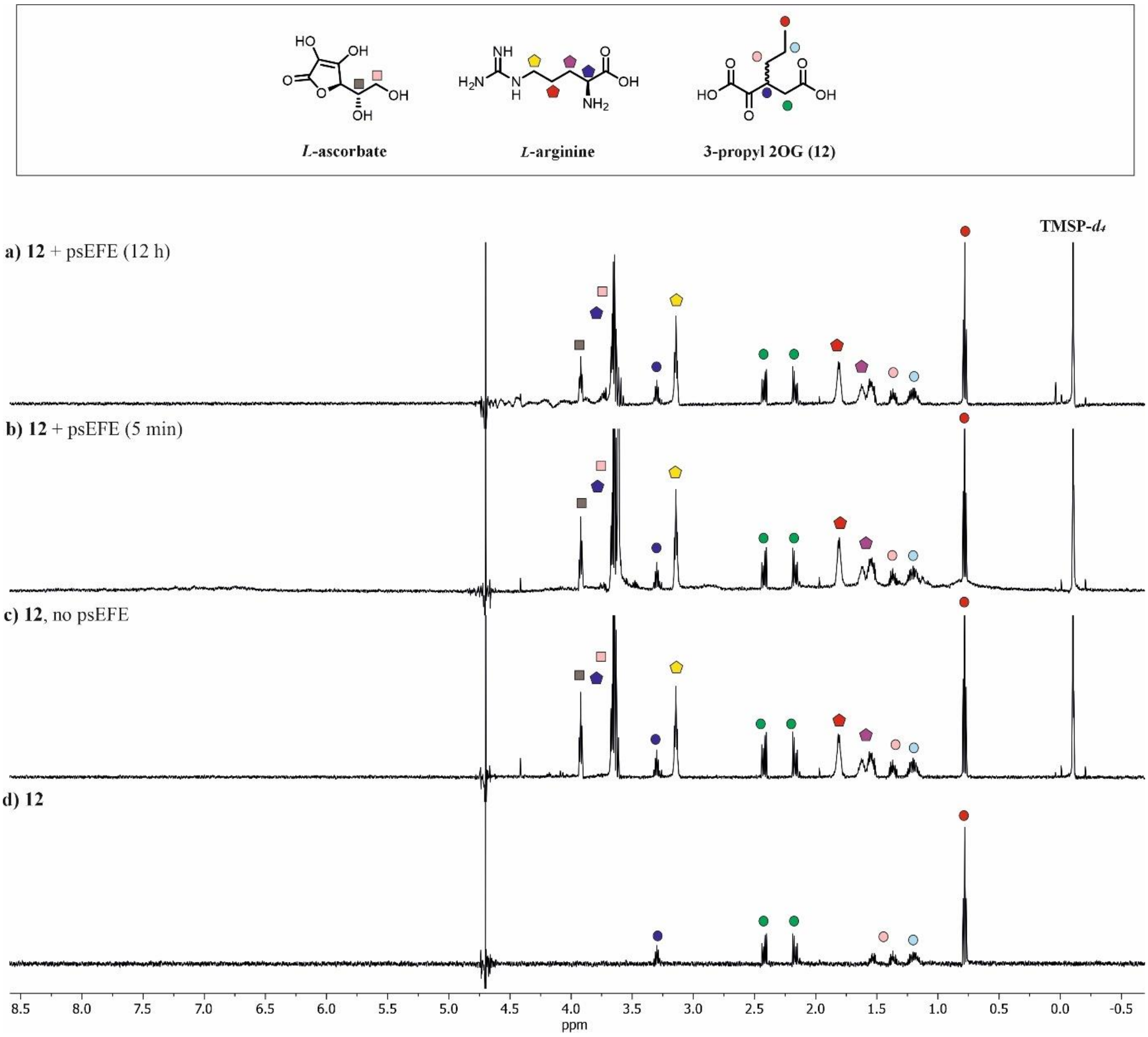

Supporting Figure S50. Analysis of psEFE catalysis using racemic 3-propyl-2OG (12) as a potential substrate. Representative ^{1}H NMR spectra monitoring the reaction of psEFE with racemic 3-propyl-2OG (**12**) after: **a**) overnight incubation and **b**) 5 min. **c**) ^{1}H NMR spectrum of **12** under standard conditions in the absence of psEFE; **d**) ^{1}H NMR spectrum of **12** in buffer. Conditions: 400 µM **12**, 500 µM *L*-arginine, 500 µM *L*-ascorbate, 50 µM Fe (II), 800 µM TMSP-d_4, and 30 µM psEFE in buffer (50 mM sodium phosphate, pH 7.4, 10%$_{v/v}$ D_2O). Formation of new peaks was not observed, even after incubating the reaction mixture overnight. Both the doublet observed at ~1.2 ppm and the singlet observed at ~4.4 ppm originate from the enzyme sample; both signals were observed in the enzyme-only control.

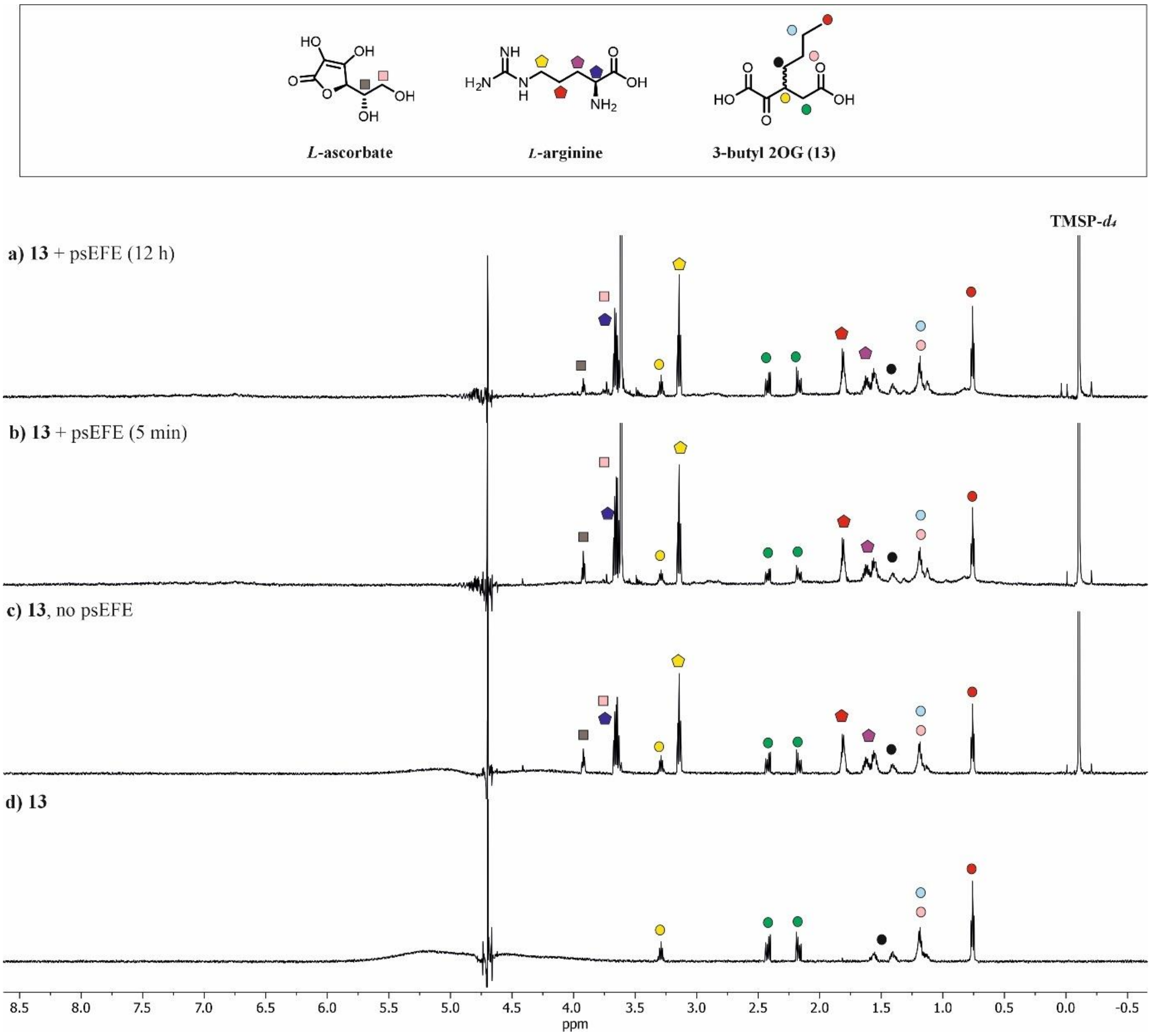

Supporting Figure S51. Analysis of psEFE catalysis using racemic 3-butyl-2OG (13) as a potential substrate. Representative ^{1}H NMR spectra monitoring the reaction of psEFE with racemic 3-butyl-2OG (**13**) after: **a**) overnight incubation and **b**) 5 min. **c**) ^{1}H NMR spectrum of **13** under standard conditions in the absence of psEFE; **d**) ^{1}H NMR spectrum of **13** in buffer. Conditions: 400 µM **13**, 500 µM *L*-arginine, 500 µM *L*-ascorbate, 50 µM Fe (II), 800 µM TMSP-d_4, and 30 µM psEFE in buffer (50 mM sodium phosphate, pH 7.4, 10%$_{v/v}$ D_2O). Formation of new peaks was not observed, even after incubating the reaction mixture overnight. Both the doublet observed at ~1.2 ppm and the singlet observed at ~4.4 ppm originate from the enzyme sample; both signals were observed in the enzyme-only control.

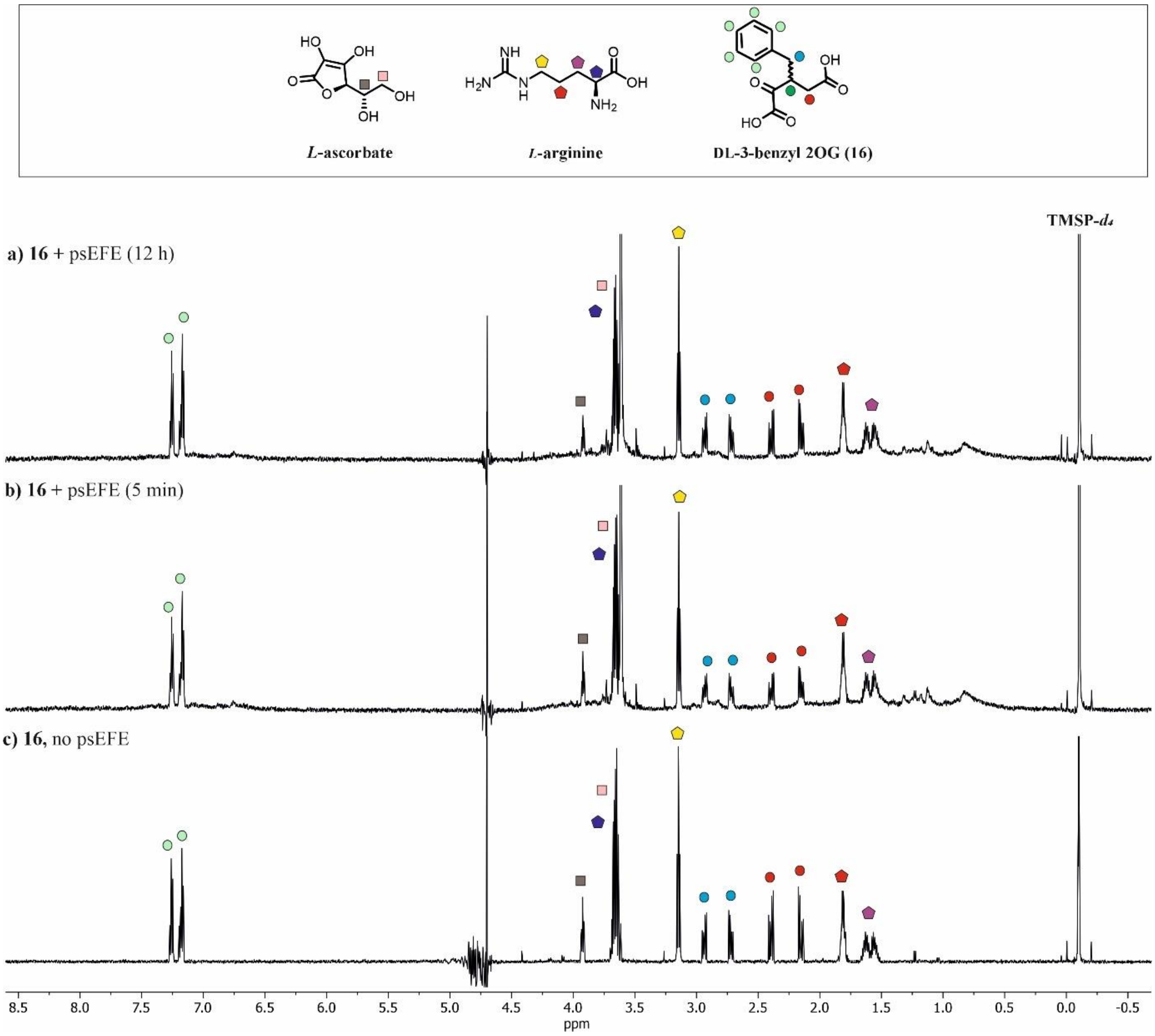

Supporting Figure S52. Analysis of psEFE catalysis using racemic 3-benzyl-2OG (16) as a potential substrate. Representative ^{1}H NMR spectra monitoring the reaction of psEFE with racemic 3-benzyl-2OG (**16**) after: **a**) overnight incubation and **b**) 5 min. **c**) ^{1}H NMR spectrum of **16** under standard conditions in the absence of psEFE; **d**) ^{1}H NMR spectrum of **16** in buffer. Conditions: 400 µM **16**, 500 µM *L*-arginine, 500 µM *L*-ascorbate, 50 µM Fe (II), 800 µM TMSP-d_4, and 30 µM psEFE in buffer (50 mM sodium phosphate, pH 7.4, 10%$_{v/v}$ D_2O). Formation of new peaks was not observed, even after incubating the reaction mixture overnight. Both the doublet observed at ~1.2 ppm and the singlet observed at ~4.4 ppm originate from the enzyme sample; both signals were observed in the enzyme-only control.

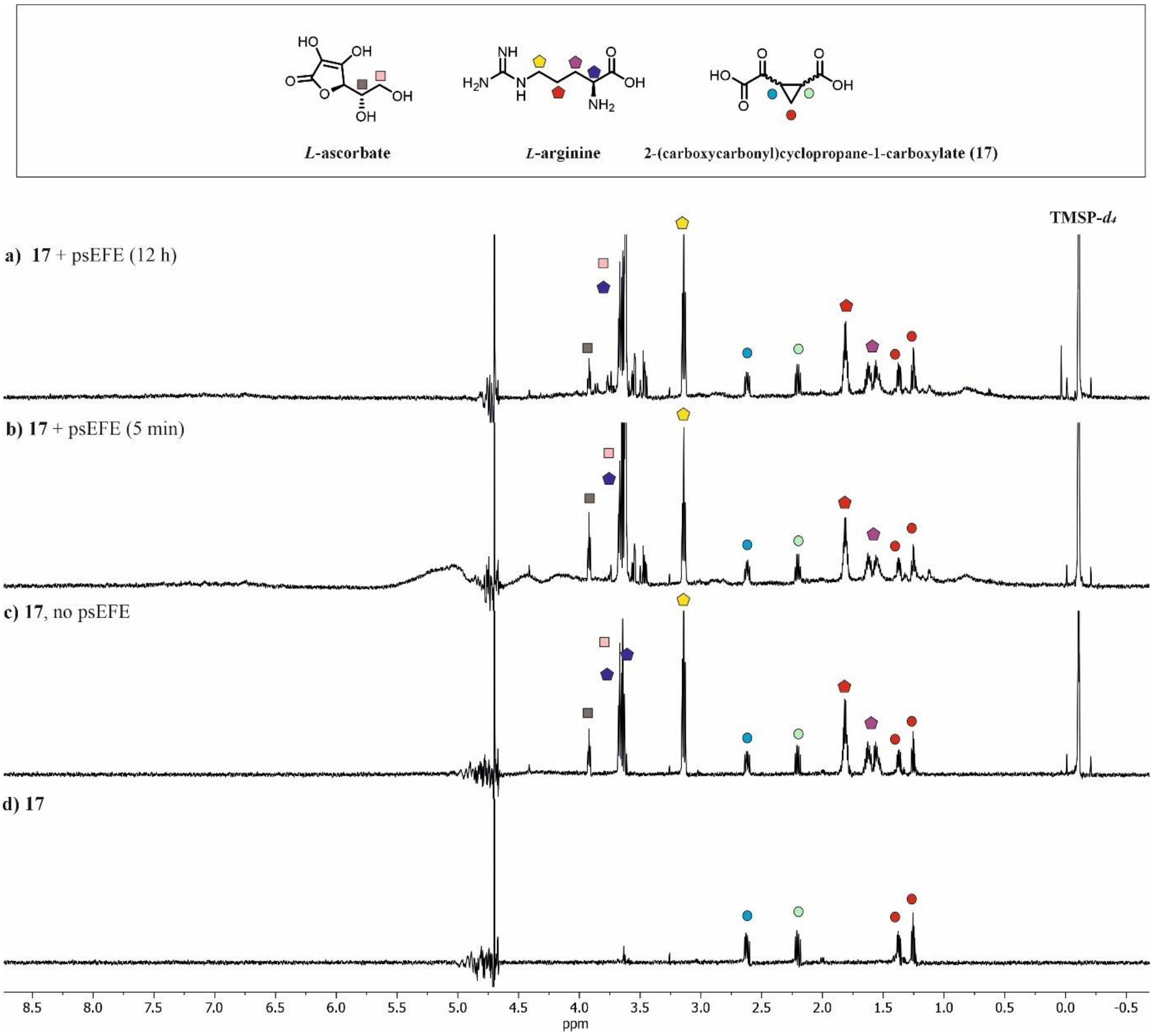

Supporting Figure S53. Analysis of psEFE catalysis using 2-(carboxycarbonyl)cyclopropane-1-carboxylate (17) as a potential substrate. Representative ^{1}H NMR spectra monitoring the reaction of psEFE with 2-(carboxycarbonyl)cyclopropane-1-carboxylate (**17**) after: **a**) overnight incubation and **b**) 5 min. **c**) ^{1}H NMR spectrum of **17** under standard conditions in the absence of psEFE; **d**) ^{1}H NMR spectrum of **17** in buffer. Conditions: 400 µM **17**, 500 µM *L*-arginine, 500 µM *L*-ascorbate, 50 µM Fe (II), 800 µM TMSP-d_4, and 30 µM psEFE in buffer (50 mM sodium phosphate, pH 7.4, 10%$_{v/v}$ D_2O). Formation of new peaks was not observed, even after incubating the reaction mixture overnight. Both the doublet observed at ~1.2 ppm and the singlet observed at ~4.4 ppm originate from the enzyme sample; both signals were observed in the enzyme-only control.

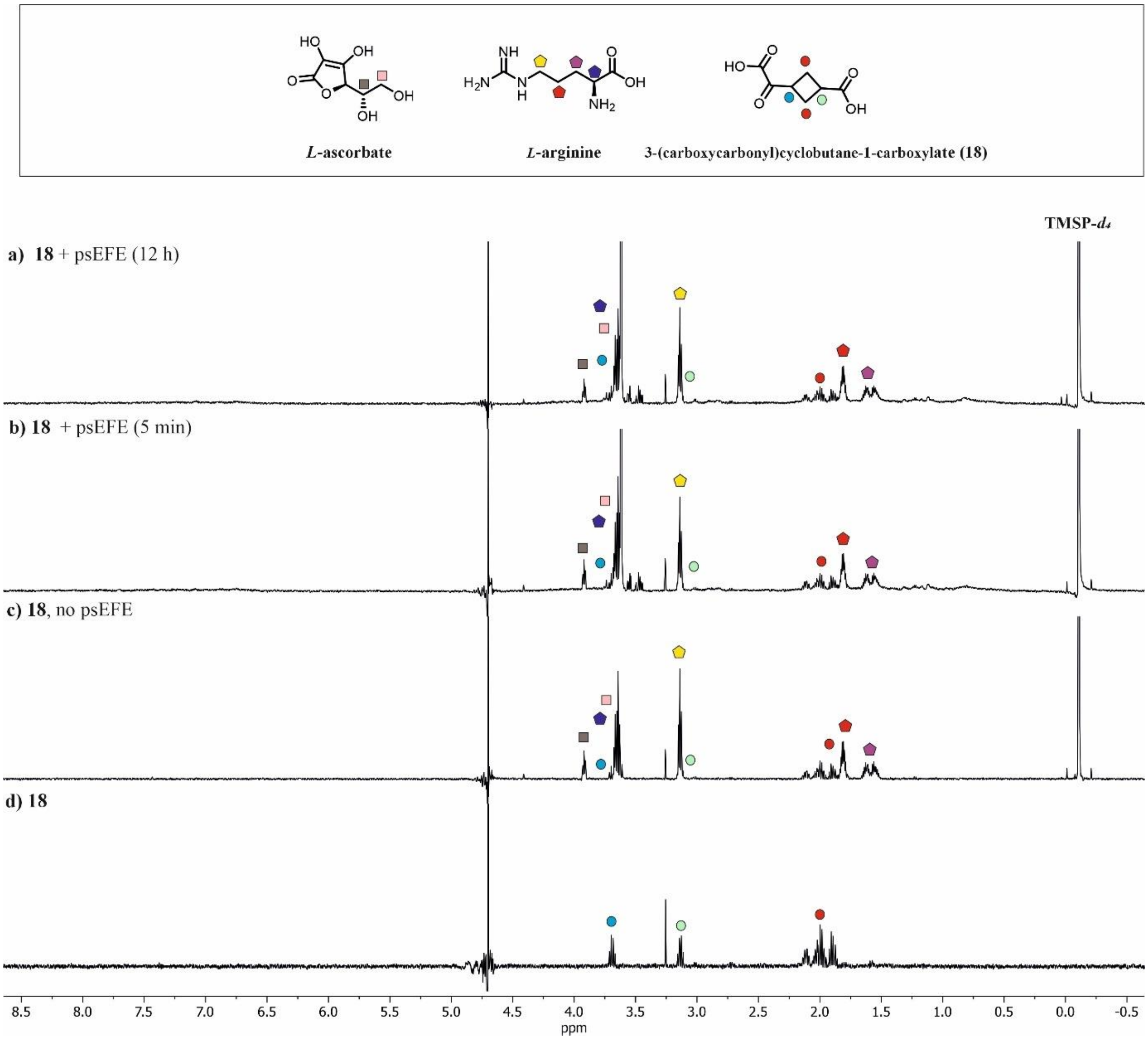

Supporting Figure S54. Analysis of psEFE catalysis using 3-(carboxycarbonyl)cyclobutane-1-carboxylate (18) as a potential substrate. Representative ^{1}H NMR spectra monitoring the reaction of psEFE with 3-(carboxycarbonyl)cyclobutane-1-carboxylate (**18**) after: **a**) overnight incubation and **b**) 5 min. **c**) ^{1}H NMR spectrum of **18** under standard conditions in the absence of psEFE; **d**) ^{1}H NMR spectrum of **18** in buffer. Conditions: 400 µM **18**, 500 µM *L*-arginine, 500 µM *L*-ascorbate, 50 µM Fe (II), 800 µM TMSP-d_4, and 30 µM psEFE in buffer (50 mM sodium phosphate, pH 7.4, 10%$_{v/v}$ D_2O). Formation of new peaks was not observed, even after incubating the reaction mixture overnight. Both the doublet observed at ~1.2 ppm and the singlet observed at ~4.4 ppm originate from the enzyme sample; both signals were observed in the enzyme-only control.

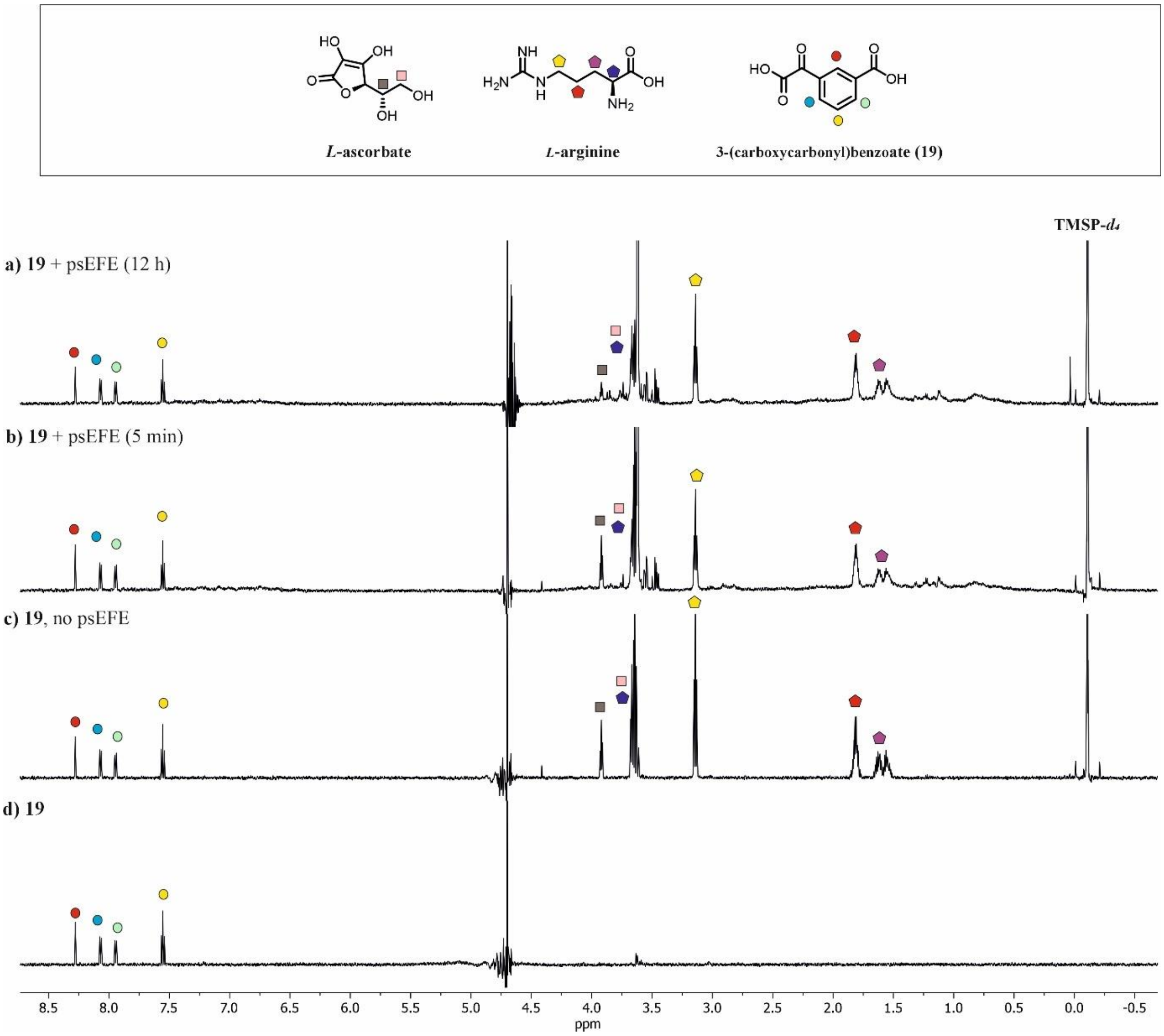

Supporting Figure S55. Analysis of psEFE catalysis using 3-(carboxycarbonyl)benzoate (19) as a potential substrate. Representative ^{1}H NMR spectra monitoring the reaction of psEFE with 3-(carboxycarbonyl)benzoate (**19**) after: **a**) overnight incubation and **b**) 5 min. **c**) ^{1}H NMR spectrum of **19** under standard conditions in the absence of psEFE; **d**) ^{1}H NMR spectrum of **19** in buffer. Conditions: 400 µM **19**, 500 µM *L*-arginine, 500 µM *L*-ascorbate, 50 µM Fe (II), 800 µM TMSP-*d*$_4$, and 30 µM psEFE in buffer (50 mM sodium phosphate, pH 7.4, 10%$_{v/v}$ D_2O). Formation of new peaks was not observed, even after incubating the reaction mixture overnight. Both the doublet observed at ~1.2 ppm and the singlet observed at ~4.4 ppm originate from the enzyme sample; both signals were observed in the enzyme-only control.

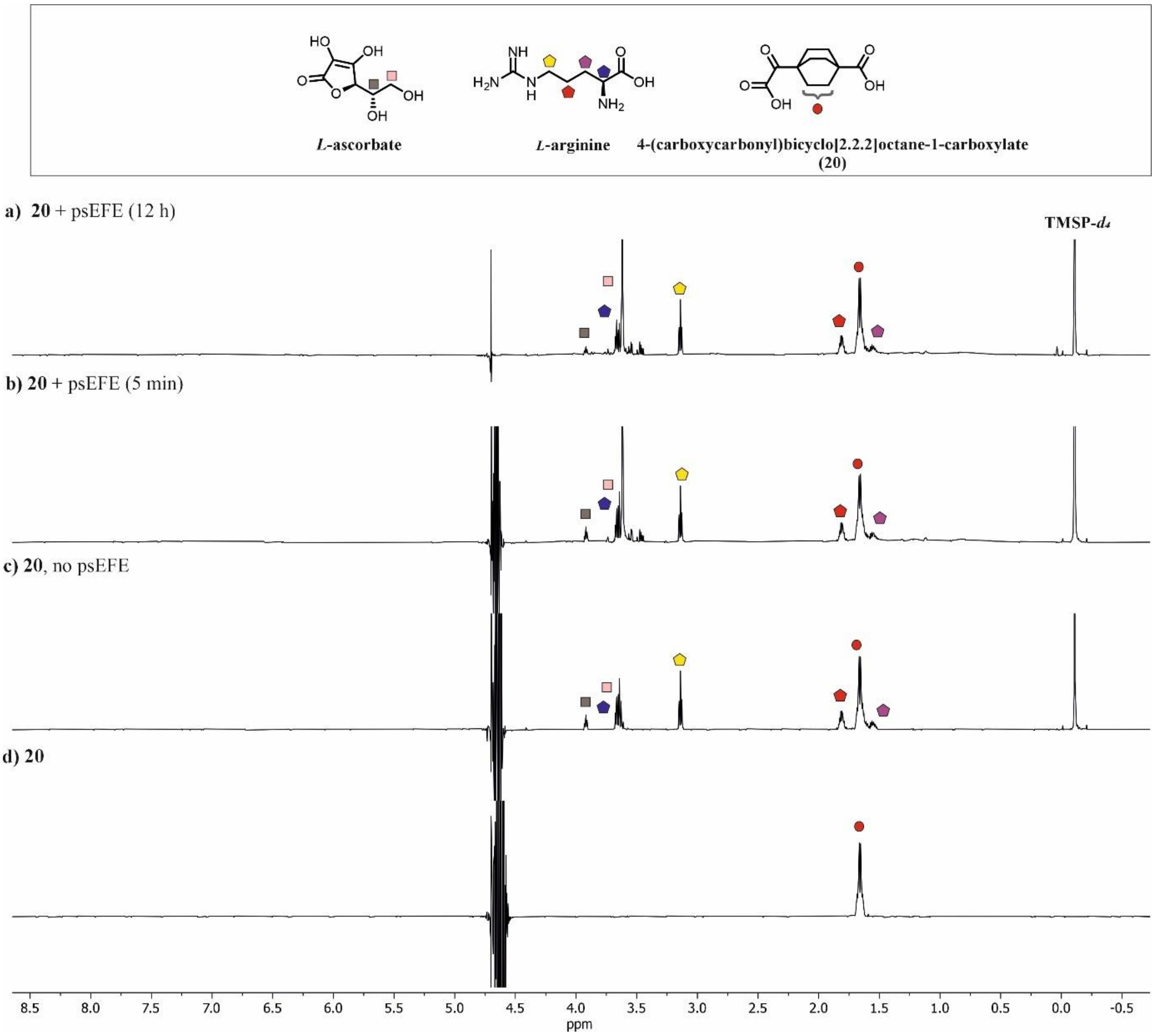

Supporting Figure S56. Analysis of psEFE catalysis using 4-(carboxycarbonyl)bicyclo[2,2,2]octane-1-carboxylate (20) as a potential substrate. Representative ^{1}H NMR spectra monitoring the reaction of psEFE with 4-(carboxycarbonyl)bicyclo[2,2,2]octane-1-carboxylate (**20)** after: **a**) overnight incubation and **b**) 5 min. **c**) ^{1}H NMR spectrum of **20** under standard conditions in the absence of psEFE; **d**) ^{1}H NMR spectrum of **20** in buffer. Conditions: 400 µM **20**, 500 µM *L*-arginine, 500 µM *L*-ascorbate, 50 µM Fe (II), 800 µM TMSP-d_4, and 30 µM psEFE in buffer (50 mM sodium phosphate, pH 7.4, 10%$_{v/v}$ D_2O). Formation of new peaks was not observed, even after incubating the reaction mixture overnight. Both the doublet observed at ~1.2 ppm and the singlet observed at ~4.4 ppm originate from the enzyme sample; both signals were observed in the enzyme-only control.

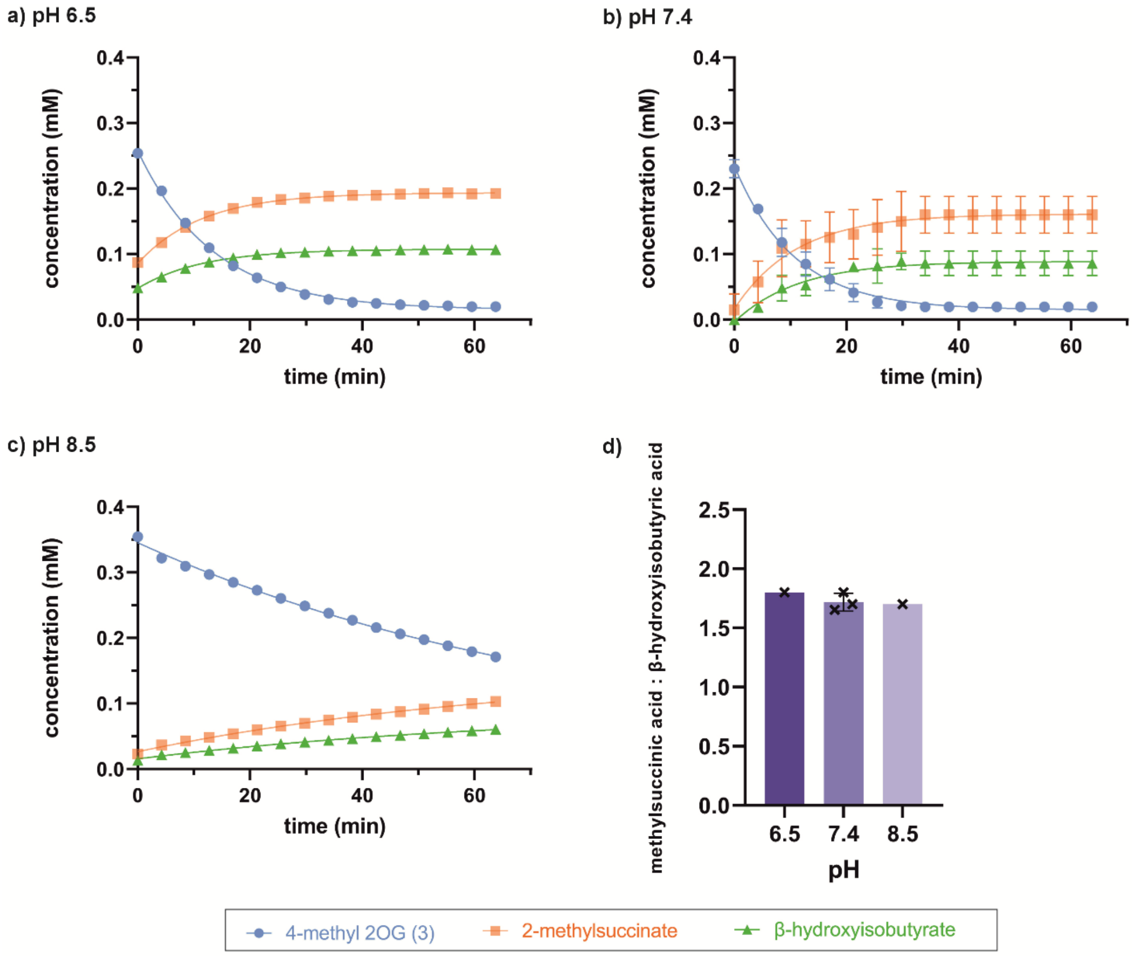

Supporting Figure S57. The product ratio of psEFE catalysis with racemic 4-methyl 2OG (3) as a (co)substrate is not affected by the tested conditions. The 2-methylsuccinate (**3a**):β-hydroxyisobutyrate (**3b**) ratios (~1.8:1) estimated by ^{1}H NMR (600 MHz) analysis of the crude reaction mixtures do not appear to vary within the tested pH range: (**a**) pH 6.5, (**b**) pH 7.4, and (**c**) pH 8.5. The time scales were calibrated to the end of acquisition of the first ^{1}H NMR experiment post addition of psEFE to the reaction mixture (t = 0 min), by which time low levels of conversion were manifest. (**d**) 2-Methylsuccinate (**3a**):β-hydroxyisobutyrate (**3b**) ratios. Conditions: 400 μM **3**, 500 μM L-arginine, 500 μM L-ascorbate, 50 μM Fe(II), 800 μM TMSP-d_4, and 10 μM psEFE in buffer (50 mM sodium phosphate, 10%$_{v/v}$ D_2O) at 25 °C.

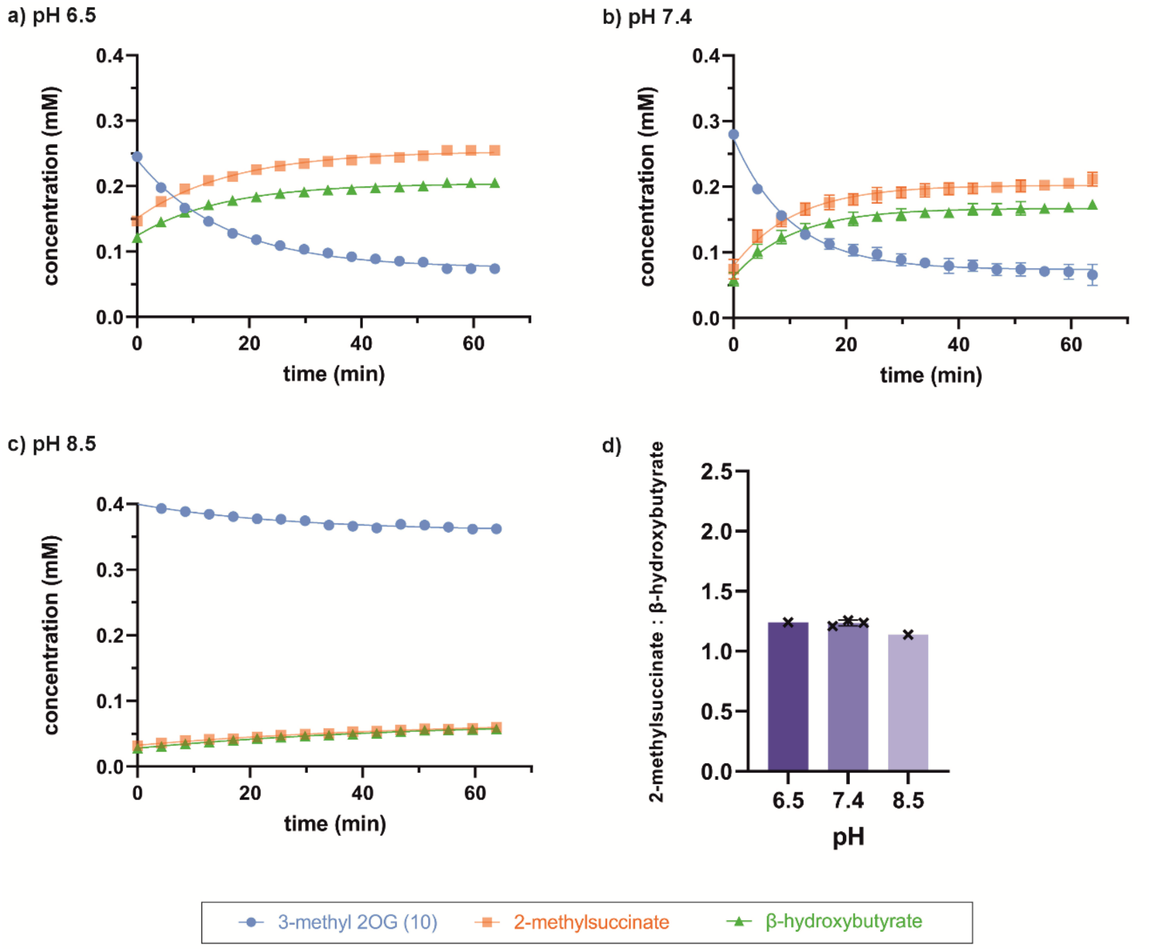

Supporting Figure S58. The product ratio of psEFE catalysis with racemic 3-methyl 2OG (10) as a (co)substrate is not affected by the tested conditions. The 2-methylsuccinate (**3a**):β-hydroxybutyrate (**10b**) ratios (~1.2:1) estimated by ^{1}H NMR (600 MHz) analysis of the crude reaction mixtures do not appear to vary within the tested pH range: (**a**) pH 6.5, (**b**) pH 7.4, and (**c**) pH 8.5. The time scales were calibrated to the end of acquisition of the first ^{1}H NMR experiment post addition of psEFE to the reaction mixture (t = 0 min), by which time low levels of conversion were manifest. (**d**) 2-Methylsuccinate (**3a**):β-hydroxyisobutyrate (**3b**) ratios. Conditions: 400 µM **10**, 500 µM L-arginine, 500 µM L-ascorbate, 50 µM Fe(II), 800 µM TMSP-d_4, and 10 µM psEFE in buffer (50 mM sodium phosphate, 10%$_{v/v}$ D_2O) at 25 °C.

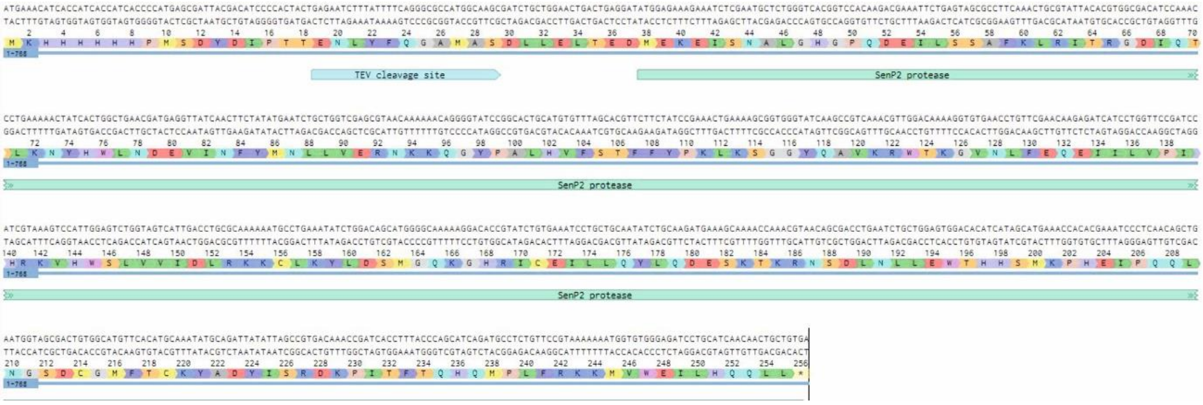

Supporting Figure S59. DNA and amino acid sequence of the SenP2 protease construct used during the production of psEFE.

3. Supporting Tables

Supporting Table S1. Product ratios of psEFE-catalyzed reactions with 2OG derivatives.[a]

	2OG derivative	diacid:alkene ratio	diacid:alcohol ratio	conversion
i	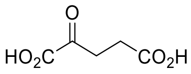<br>**2OG (1)**	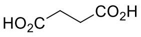 **succinate** ; $H_2C=CH_2$ **ethylene**<br>1.0 ± 0.1:1	no alcohol product formed under the tested conditions	>95%
ii	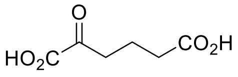<br>**2OA (2)**	no alkene product formed under the tested conditions	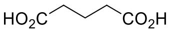 **2a** ; 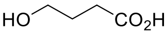 **2b**<br>2.1 ± 0.1 : 1	~60%
iii	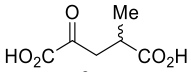<br>**3**	no alkene product formed under the tested conditions	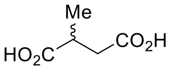 **3a** ; 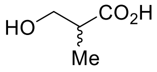 **3b**<br>1.8 ± 0.1 : 1	>95%
iv	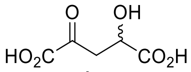<br>**4**	no alkene product formed under the tested conditions	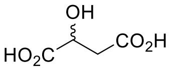 **4a** ; 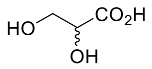 **4b**<br>2.3 ± 0.1 : 1	~90%
v	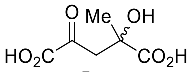<br>**5**	no alkene product formed under the tested conditions	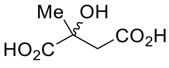 **5a** ; 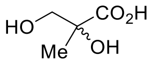 **5b**<br>1.2 ± 0.1 : 1	~30%
vi	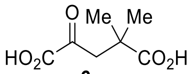<br>**6**	no alkene product formed under the tested conditions	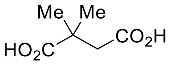 **6a** ; 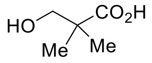 **6b**<br>1.3 ± 0.1 : 1	~80%
vii	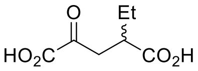<br>**7**	no alkene product formed under the tested conditions	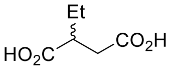 **7a** ; 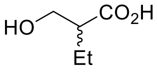 **7b**<br>3.2 ± 0.1 : 1	>95%
viii	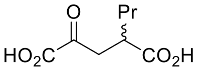<br>**8**	no alkene product formed under the tested conditions	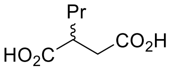 **8a** ; 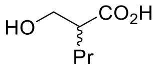 **8b**<br>4.1 ± 0.1 : 1	~70%
ix	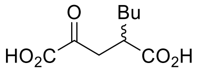<br>**9**	no alkene product formed under the tested conditions	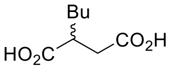 **9a** ; 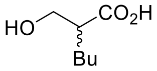 **9b**<br>~1 : 0	~50%
x	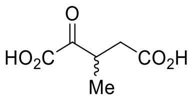<br>**10**	no alkene product formed under the tested conditions	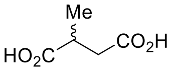 **3a** ; 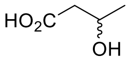 **10b**<br>1.2 ± 0.1 : 1	>75%

	2OG derivative	diacid:alkene ratio	diacid:alcohol ratio	conversion
[b]xi	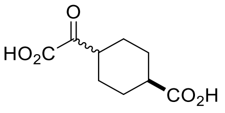 **14** ***trans:cis*, 5:1**	no alkene product formed under the tested conditions	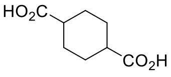 **14a** ***isomeric mixture*** / 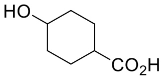 **14b** ***isomeric mixture*** ~1 : 0	[c]not determined
xii	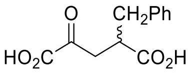 **15**	no alkene product formed under the tested conditions	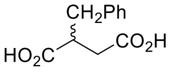 **15a** / 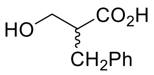 **15b** ~1 : 0	[d]not determined

a) 2OG derivatives were typically prepared from cyanosulfur ylids as racemic mixtures as reported (2). Note, there is a possibility for stereoselective conversion; b) **14** was used as a mixture of diastereomers, dr (*trans*:*cis*) = 5:1 (2); c) the identity of the diacid reaction product was confirmed by spiking the reaction mixture with a synthetic sample of **14a**. Levels of conversion could not be accurately determined by ^{1}H NMR due to overlapping signals of starting material (**14**) and product (**14a**). Spiking with the potential corresponding authentic alcohol product (**14b**) revealed that it is not formed, within limits of detection. **14a** and **14b** were commercially obtained as a mixture of *cis* and *trans* isomers; d) the identity of the diacid reaction product was confirmed by spiking the reaction mixture with a synthetic sample of **15a**. Levels of conversion could not be accurately determined by ^{1}H NMR due to overlapping signals of starting material (**15**) and product (**15a**). Spiking with the potential corresponding authentic alcohol product (**15b**) revealed that it is not formed, within limits of detection. Conditions: 400 µM 2-oxoacid, 500 µM L-arginine, 500 µM L-ascorbate, 50 µM Fe(II), 800 µM TMSP-d_4, 2 µM psEFE (for 2OG) or 10-30 µM psEFE (for 2OA and 2OG derivatives) in buffer (50 mM sodium phosphate, pH 7.4, 10%$_{v/v}$ D_2O) at 25 °C. The results are the mean of independent triplicates (n = 3; mean ± SD).

4. References

1. Gates, E. L., Bradley, J. P., Berry, D. B. G., Nilsson, M., Morris, G. A., Adams, R. W., and Castañar, L. (2024) Solvent suppression in pure shift NMR. *Anal. Chem.* **96**, 3879-3885

2. Brewitz, L., Nakashima, Y., and Schofield, C. J. (2021) Synthesis of 2-oxoglutarate derivatives and their evaluation as cosubstrates and inhibitors of human aspartate/asparagine-β-hydroxylase. *Chem. Sci.* **12**, 1327-1342

3. Adams, R. W., Holroyd, C. M., Aguilar, J. A., Nilsson, M., and Morris, G. A. (2013) "Perfecting" WATERGATE: clean proton NMR spectra from aqueous solution. *Chem. Commun.* **49**, 358-360

4. Pohl, L., and Eckle, M. (1969) Sodium 3-trimethylsilyltetradeuteriopropionate, a new water-soluble standard for ^{1}H-NMR. *Angew. Chem. Int. Ed.* **8**, 381

5. Castañar, L. (2017) Pure shift ^{1}H NMR: what is next? *Magn. Reson. Chem.* **55**, 47-53

6. Foroozandeh, M., Morris, G. A., and Nilsson, M. (2018) PSYCHE pure shift NMR spectroscopy. *Chem. Eur. J.* **24**, 13988-14000

7. Zangger, K. (2015) Pure shift NMR. *Prog. Nucl. Magn. Reson. Spectrosc.* **86-87**, 1-20

8. Wishart, D. S., Knox, C., Guo, A. C., Eisner, R., Young, N., Gautam, B., et al. (2009) HMDB: a knowledgebase for the human metabolome. *Nucleic Acids Res.* **37**, D603-D610
